# Synthesis, Carbonic Anhydrase II/IX/XII Inhibition, DFT, and Molecular Docking Studies of Hydrazide-Sulfonamide Hybrids of 4-Methylsalicyl- and Acyl-Substituted Hydrazide

**DOI:** 10.1155/2022/5293349

**Published:** 2022-02-24

**Authors:** Adil Khushal, Amara Mumtaz, Wamda Ahmed Shadoul, Syeda Huda Mehdi Zaidi, Hummera Rafique, Abida Munir, Aneela Maalik, Syed Jawad Ali Shah, Ayesha Baig, Wajiha Khawaja, Mariya al-Rashida, Muhammad Ali Hashmi, Jamshed Iqbal

**Affiliations:** ^1^Department of Chemistry, COMSATS University Islamabad, Abbottabad Campus, Pakistan; ^2^Center for Advance Drug Research, COMSATS University Islamabad, Abbottabad Campus, Pakistan; ^3^Department of Chemistry, University of Education, Attock Campus Attock 43600, Pakistan; ^4^Department of Chemistry, University of Gujrat, Gujrat, Pakistan; ^5^Department of Chemistry, COMSATS University Islamabad, Islamabad Campus, Pakistan; ^6^Department of Biotechnology, COMSATS University Islamabad, Abbottabad Campus, Pakistan; ^7^Department of Chemistry, Forman Christian College, Lahore, Pakistan

## Abstract

Carbonic anhydrases (CAs and EC 4.2.1.1) are the Zn^2+^ containing enzymes which catalyze the reversible hydration of CO_2_ to carbonate and proton. If they are not functioning properly, it would lead towards many diseases including tumor. Synthesis of hydrazide-sulfonamide hybrids **(19-36)** was carried out by the reaction of aryl **(10-11)** and acyl **(12-13)** hydrazides with substituted sulfonyl chloride **(14-18)**. Final product formation was confirmed by FT-IR, NMR, and EI-MS. Density functional theory (DFT) calculations were performed on all the synthesized compounds to get the ground-state geometries and compute NMR properties. NMR computations were in excellent agreement with the experimental NMR data. All the synthesized hydrazide-sulfonamide hybrids were *in vitro* evaluated against CA II, CA IX, and CA XII isozymes for their carbonic anhydrase inhibition activities. Among the entire series, only compounds **22**, **32**, and **36** were highly selective inhibitors of *h*CA IX and did not inhibit *h*CA XII. To investigate the binding affinity of these compounds, molecular docking studies of compounds **32** and **36** were carried out against both *h*CA IX and *h*CA XII. By using BioSolveIT's SeeSAR software, further studies to provide visual clues to binding affinity indicate that the structural elements that are responsible for this were also studied. The binding of these compounds with *h*CA IX was highly favorable (as expected) and in agreement with the experimental data.

## 1. Introduction

**Figure 1:**
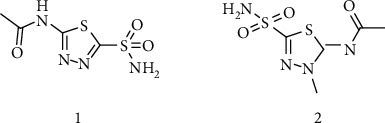


Carbonic anhydrases (CAs and EC 4.2.1.1) are the Zn^2+^ containing metalloenzyme, belong to the superfamily of enzymes, and are found in all life kingdoms. CAs belong to seven different genetic families, sharing the common mission of catalyzing the reversible hydration of CO_2_ to carbonate and proton [1]. In addition to CO_2_ regulation, they are also responsible for lipogenesis, gluconeogenesis, and ureagenesis. Vertebrate carbonic anhydrases belong to the *α*-CA class with 16 isozymes known so far. All these isozymes differ from one another due to their tissue specificity and localization in the cell. Many of them are cytosolic like CA I, CA II, CA III, CA VII, CA VIII, CA X, CA XI, and CA XIII. Some are membrane bound like CA IV, CA IX, CA XII, CA XIV, and CA XV. Some are mitochondrial like CAVA and CAVB, while CA VI is secreted into the cell's cytoplasm [2, 3]. So far, nine CA isozymes were detected in the human central nervous system; it is believed that they are involved in many crucial functions, but the exact mechanism is not yet fully understood. Apart from their vital role in maintaining many important physiological processes, deregulation of carbonic anhydrases is also known to be associated with many pathologies, such as cerebral and retinal edema, glaucoma, epilepsy, stroke, retinitis pigmentosa, and growth of the tumor cells [2]. The overexpression of CA IX is lately been associated with the proliferation of the tumor cells providing a suitable environment for the tumor cells to grow; it has also been related to the poor response of patients to common chemotherapeutic reagents [4]. Acetazolamide **(1)** and methazolamide **(2)** are the clinically recognized carbonic anhydrase inhibitors (CAIs) and were tested on some forms of epilepsy in the 1970s (Figure 1). Lately, CAIs have been used in combination with other medicines in the treatment of obstructive sleep apnea; other CAIs of the brain isozymes are applied in the treatment of idiopathic intracranial hypertension (IIH), cerebral ischemia, neuropathic pain, and migraine [5].

As CAs are the zinc metalloenzymes, so, one of the classic types of carbonic anhydrase inhibitors is the zinc ion (Zn^2+^) binders. These CAIs coordinate to the catalytically crucial Zn^2+^ from the enzyme site. During this inhibition type, Zn^2+^ could also be in tetrahedral or trigonal bipyramidal geometries. Sulfonamides, sulfamides, sulfamates, most anions, dithiocarbamates, carboxylates, and hydroxamates are the known CAIs that bind through this pathway [6]. Presence of the primary amino group make the sulphonamide excellent carbonic anhydrase inhibitor [7] but latest research reveals that secondary and tertiary sulphonamides also possess selective carbonic anhydrase inhibition activities [8–11].

Sulfonamides are considered a very important class of drugs; their major use is as carbonic anhydrase inhibitors and antibacterial agents [12]. The compounds that have another electron-withdrawing group or atom attached to the sulfanoyl group resulting in a compound with the -NH-SO_2_-NH_2_ group were found to be equipotent to free sulfonamides and sometimes stronger inhibitors [13]. This led us to the synthesis of some aryl- and acyl-substituted hydrazide-sulfonamide hybrids and *in vitro* investigation of their carbonic anhydrase inhibition activities against CA II, CA IX, and CA XII. These isozymes were selected because CA II is the most active and abundant isozyme throughout the human body; the overexpression of CA IX and CA XII has always been related to the proliferation of the tumor cells [4]. NMR spectral data was verified by DFT studies. The molecular docking studies of the most potent derivatives and further confirmation by binding affinities studies were also carried out.

## 2. Results and Discussion

### 2.1. Chemistry

**Scheme 1:**
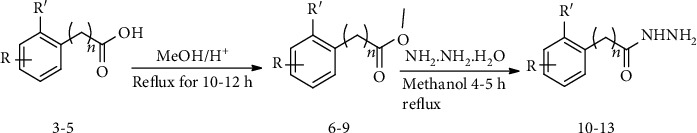
Synthesis of hydrazide (10-13).

Synthesis of hydrazide-sulfonamide hybrids **(19-36)** was carried out starting from the synthesis of substituted hydrazides **(10-13)** [14]. For this reason, the methanolic solution of methyl esters **(6-9)** was refluxed with hydrazine monohydrate 64% to get respective hydrazides **(10-13)**. Physical data of hydrazides **(10-13)** is presented in Table 1. Schematic representation of the reactions is presented in Scheme 1.

Structural confirmation of hydrazide synthesis was done by using FT-IR and ^1^HNMR. In FT-IR spectrum, N-H stretching peak at 3018-3406 cm^−1^ and NH_2_ asymmetric stretch at 3229-3309 cm^−1^ while symmetric stretch at 3212-3329 cm^−1^ confirmed that hydrazide **(10-13)** has been synthesized. Further structural confirmation was done by the appearance of stretching peaks like C=O at 1612 cm^−1^. C-H stretching peak at 3012 cm^−1^ and C=C stretch at 1495 cm^−1^ of benzene ring also support the presence of functional groups of hydrazides. The presence of the methoxy group substitution in hydrazide **10** and **13** at the benzene ring showed asymmetric and symmetric stretching peaks at 1253 and 1155 cm^−1^, respectively. In the ^1^H NMR spectrum of the hydrazides **(10-13)**, a broad singlet of NH appeared at 6.73-9.02 ppm while a broad singlet of the NH_2_ group at 3.23-4.26 ppm confirmed the formation of compound **(10-13)**. Two doublets at 8.07 and 7.96 ppm and a singlet at 6.75 ppm confirmed the presence of all aromatic signals [14].

**Scheme 2:**
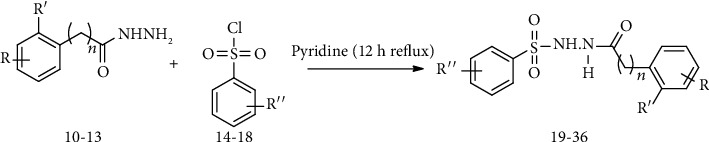
Synthesis of hydrazide sulfonamide (19-36).

For the synthesis of the compounds **(19-36)**, hydrazide **(10-13)** (0.5 mmol) was stirred with substituted benzenesulfonyl chloride **(14-18)** (0.5 mmol) in pyridine (Scheme 2). Physical data of the compounds **(19-36)** is presented in Table 2.

In the FT-IR spectrum, the appearance of NH stretching peak at 3321-3375 cm^−1^ and asymmetric and symmetric stretching peaks of S=O at 1332-1387 cm^−1^ and 1189-1195 cm^−1^, respectively, confirmed the hydrazide-sulfonamide hybrid synthesis **(19-36)**. Moreover, asymmetric and symmetric streching at 1553 cm^−1^ and 1376 cm^−1^ confirmed the nitro group, while C-H stretching peak at 3095-3132 cm^−1^ confirmed the aromatic rings. Stretching peak of C=O at 1622-1670 cm^−1^, stretching peak of aliphatic C-H at 3132 cm^−1^, and C-O stretching peak at 1230-1263 cm^−1^ were observed. The structures of all the synthesized compounds were confirmed by the ^1^HNMR data. In the ^1^H NMR data of hydrazide-sulfonamide hybrids **(19-36)**, two singlets appeared in the range of 10.45-10.61 and 9.56-10.33 ppm for NH groups which confirmed the formation of the product. Four doublets of all the aromatic protons of both benzene rings appeared in the aromatic region. The methyl group showed signal at 2.32-2.28 ppm [15]. To confirm the structures of the hydrazide-sulfonamide hybrids **(19-36)**, the ^13^CNMR spectra of the selected compounds were taken. The appearance of the signal at 164.3-164.8 ppm for S=O and 157.5-157.6 ppm for C=O groups confirmed the synthesis of hydrazide-sulfonamide hybrids. To further justify the structures of hydrazide-sulfonamide hybrids EI-MS of the selected compounds were taken and the appearance of [M + 1] molecular ions, peaks confirmed the structures of our desired products. To check the purity of the synthesized compounds, HPLC spectra of the selected compounds were taken in reverse phase in acetonitrile/water (1 : 1) mixture with a 10-11-minute retention time.

### 2.2. Computational Studies

**Figure 2:**
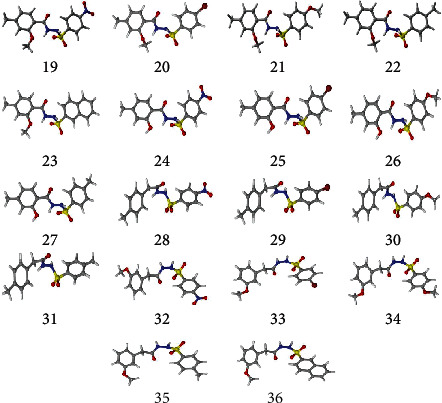
Optimized geometries of all the compounds under study (**19**-**36**) at PBE0-D3BJ/def2-TZVP/SMD_Solvent_ (Solvent = CDCl_3_, DMSO experimental solvent for NMR spectroscopy) level of the theory. In 3D models, grey color represents carbon, white represents hydrogens, yellow is for sulfur, red color is for oxygen, brown represents bromine, and blue color shows nitrogen atoms.

All the synthesized compounds have been studied using the density functional theory (DFT) computations to gain an insight into their electronic structure and compute their NMR chemical shifts. The compounds have been subjected to geometry optimization using the PBE0-D3BJ/def2-TZVP/SMD_Solvent_ (Solvent = DMSO or chloroform) level of theory [16, 17] followed by their frequency calculations to verify that they are true minima on the potential energy surface (PES).  Figure 2 shows their optimized geometries.

For organic chemists, nuclear magnetic resonance (NMR) is of prime importance and can be used as a vital technique to determine and verify the structures of synthesized molecules. DFT computations of NMR chemical shifts can yield an accurate NMR dataset that can be compared with the experimental data. All of the modeled compounds' NMR calculations have been performed on the same theoretical level as the optimizations, and the results are compared with the experimental chemical shifts. Methanol and benzene have been employed as reference standards for sp^3^ and sp^2^ carbons due to their good and effective results, as formerly demonstrated by Perdew et al. [18]. The ^1^H-NMR data of compound **19** is given in Table 3. The supporting information contains a comparison of all of the other compounds. Furthermore, with a mean absolute error (MAE) of 0.19 ppm only, it is evident that the NMR calculation methodology has worked really well. Consequently, some compounds' NMR data could not be obtained in a sufficient yield to get their precise experimental NMR; however, its accurate prediction from the computations can be utilized as a guide for the production of these compounds.

### 2.3. Carbonic Anhydrase Inhibition Studies

All the synthesized compounds were tested for their inhibition activity against the three isozymes of CAs, CA II, CA IX, and CA XII, using the optimized colorimetric method [19], and the results are shown in Table 4.

The synthesized compounds showed results against the three isozymes with relatively less Ki values against CA IX and CA XII. In the case of sulfonamide derivatives synthesized from salicylic acid hydrazide, the presence of the methoxy group at the orthoposition to the carbonyl group decreased the activity of compounds **19**, **20**, **21**, **22**, and **23**, while the unprotected hydroxyl group at the same position has clearly enhanced the activity of the compounds **24**, **25**, **26**, and **27** against CA IX and CA XII [20]. In the case of phenyl-substituted acyl hydrazide derivatives, an enhancement of the activity against the three isozymes was observed in compounds **28**, **29**, **30**, and **31**. While for the compounds having 3-methoxyphenyl acyl derivatives, **32**, **33**, **34**, **35**, and **36** lead to variation between the three isozymes and the enhancement or decrease in the activity of the compound depended on other substituents coming from phenyl sulfonyl chloride part [21].

Substitution of the benzene ring containing the sulfonamide moiety with a methoxy and one more benzene greatly enhances the activity of compounds **21** and **23**, respectively, against CA IX, also seen in **30**. The presence of a nitro group at this position enhances the activity of the compounds against CA II as seen in compounds **19, 24**, **28**, and **32**. Meanwhile, less effect of this group was observed within the activity of the compounds against CA IX and reduction in the activity of these compounds against CA XII. The presence of a bromide at this position showed variation depending of the substituents in the other benzene ring. Studies suggest that these compounds may play an important role as an anticancer agent with less side effect against the major off-target CA II which is readily available in a wide range of tissues and being the most active isozyme of the family. The presence of the specific functional groups enhanced the activity against the enzyme.

### 2.4. Molecular Docking Studies

The carbonic anhydrase isozymes (II, IX, and XII) selected for docking studies contained different sulfonamide inhibitors cocrystallized in the active pocket. These cocrystallized inhibitors were selected as reference for docking, and the docking protocol was validated after successfully reproducing the cocrystallized poses. The calculated RMSD values for reference ligands of CA II, CA IX, and CA XII were 0.90, 0.83, and 1.19 Å, respectively. The HYDE assessment shows a binding-free energy of -41, -36, and -23 KJmol^−1^, respectively.

**Figure 3:**
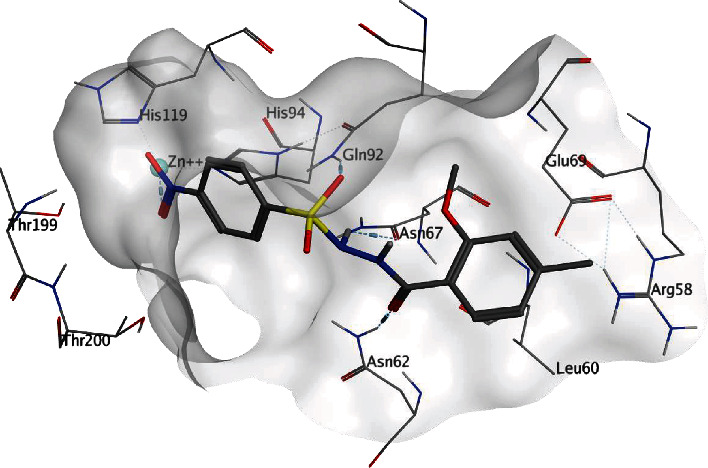
Possible binding mode of inhibitor 24 inside CA II active pocket.

Compound **24** was docked inside the active pocket of CA II with a FlexX score of -23 and HYDE score of -18 KJmol^−1^ (Figure 3). Similar to the reference cocrystallized ligand, inhibitor **24** forms hydrogen bonding interaction with residue Gln92 and a metal ion interaction with Zn^+2^ ion in the active pocket. Additionally, the residue Asn67 was found to form two hydrogen bonds with the carboxyl and amino group of compound **24**. Likewise, the hydrophobic interactions in reference cocrystallized ligand the inhibitor **24** shows hydrophobic interactions with Gln92, His94, and Val121. Additionally, hydrophobic interactions with Leu60 and Asn67 were also observed.

**Figure 4:**
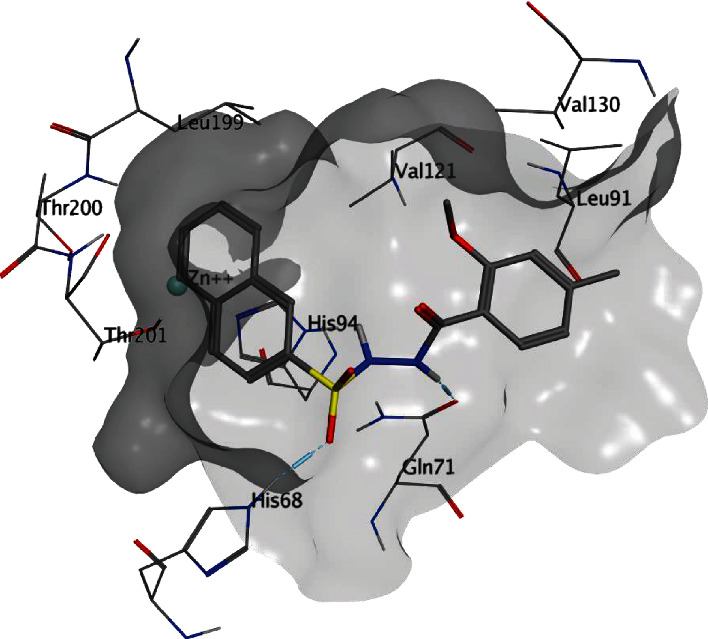
Possible binding mode of the inhibitor 23 inside the CA IX active pocket.

The inhibitor **23** (Figure 4) was found to dock inside the CA IX active pocket with a FlexX score of -24 and binding affinity (HYDE score) of -18 KJmol^−1^. Similar to the reference ligand, inhibitor **23** was found to form hydrogen bonding interaction with the amino group of the residue Gln92. Unlike the reference ligand, no metal ion interaction was found due to the bulky naphthalene group in inhibitor **23**. However, the sulfonamide group of the inhibitor adjacent to the naphthalene group was found to form hydrogen bonding interaction with residue His68. Additionally, the residue Gln71 was also found to form two hydrogen bonding interactions with amino groups of the inhibitor **23**. Similar to the reference ligand, inhibitor **23** was found to form hydrophobic interactions with His94, Val130, Leu134, and Leu199. Additionally, several other hydrophobic interactions of inhibitor **23** with residues Leu91, Gln92, Thr201, and Val121 were also found.

**Figure 5:**
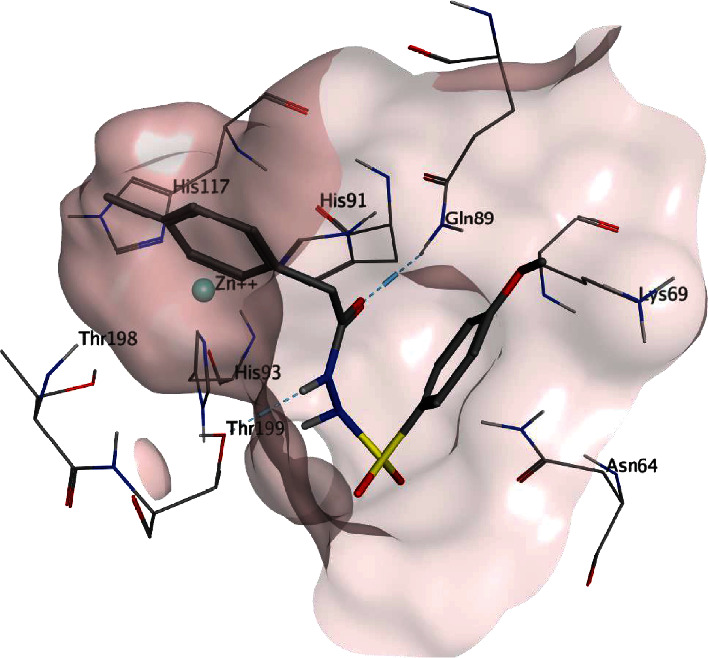
Possible binding mode of the inhibitor 30 inside the CA XII active pocket.

Docking of the inhibitor **30** (Figure 5) inside the CA XII revealed a FlexX docking score of -19 and binding affinity of -18 KJmol^−1^. The inhibitor **30** was observed to form hydrogen bonding interaction with residues Lys69, Gln89, and Thr199. Interaction with residue Thr199 was also found in the case of cocrystallized sulfonamide inhibitor in the active pocket of CA XII. Unlike the cocrystallized inhibitor's sulfonamide group interaction with Zn^+2^ ions in the active site, no such interaction was found in the case of inhibitor **30** due to methoxy substitution adjacent to its sulfonamide group. The residues Asn64, Lys69, Gln89, Val119, Val141, Leu197, Thr199, and Val206 were observed to form hydrophobic interactions and pocket lining of inhibitor **30**.

### 2.5. Investigating Selective Binding of 32 and 36 to hCA IX over hCA XII

Among the entire series, only compounds **22**, **32**, and **36** were highly selective inhibitors of hCA IX and did not inhibit hCA XII (<50% inhibition). We wanted to investigate why binding of these compounds to hCA XII is poor. For this purpose, the compounds **32** and **36** were docked against both hCA IX and hCA XII. Binding of these compounds with hCA IX was highly favorable (as expected) and in agreement with the experimental data.

**Figure 6:**
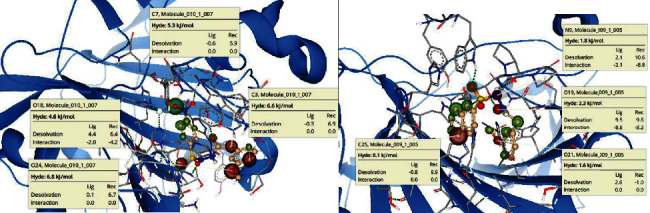
SeeSAR analysis (visual assessment of binding affinity) for compound 32 (a) and compound 36 (b) (selective hCA IX inhibitors over hCA XII) docked against hCA XII. The structural elements responsible for the favorable contribution to the overall binding affinity are shown in green coronas; the greater the sphere of the corona, the greater is the contribution. Similarly, the structural elements contributing unfavorably to the overall binding are shown in red coronas. The structural elements that are not contributing either favorably or unfavorably are not colored.

BioSolveIT's SeeSAR software [22, 23] provides visual clues to binding affinity (whether favorable or unfavorable) and also indicates the structural elements that are responsible for this. The SeeSAR analysis of compounds **32** and **36** are given in Figure 6. For compounds **32** and **36**, most of the binding modes indicated highly unfavorable binding.

**Figure 7:**
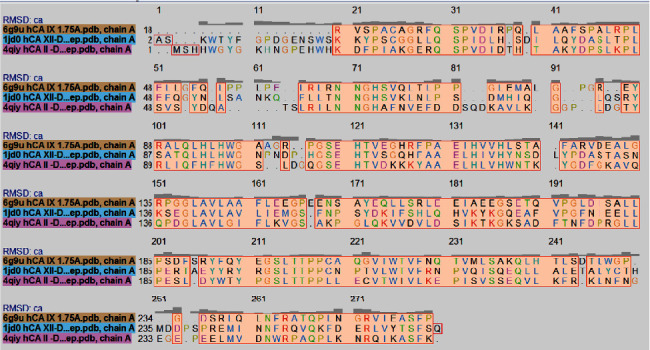
Sequence alignment of hCA II (4qiy), hCA IX (6g9u), and hCA XII (1jd0).

For compound **32** (Figure 6(a)), four structural elements were found to be highly unfavorable. One of oxygen atom of the sulfonamide group, the oxygen atom of the methoxy group, the carbon atom next to the carbon atom to which methoxy group is attached, and the carbon atom adjacent to the carbonyl group. With the exception of sulfonamide oxygen (that is involved in hydrogen bond formation with Asn62), other atoms (mentioned above) had a very high desolvation energy that had not been compensated by hydrogen bond formation. If the orientation of the inhibitor was such that result in some nonbonded interactions, the penalty on high desolvation energy would have been compensated, which is not the case; this may explain why binding of these molecules is inefficient. Similarly, for compound **36** (Figure 6(b)), four structural elements were found to be highly unfavorable. The sulfonamide NH and oxygen atom of the carbonyl group were contributing unfavorably because of high desolvation energy, although some of the unfavorable, high energy is compensated in part by the presence of hydrogen bonds that -NH is making with Pro201 and -C=O with Thr200; it is not enough and the overall contribution is still somewhat unfavorable. The other two unfavorable structural elements are the oxygen atom of the methoxy group and carbon atom (C25) of the naphthyl group. Both have high desolvation energies that has not been compensated by the formation of any nonbonded interaction (Figure 7).

## 3. Conclusions

A series of sulfonamide-hydrazide hybrids of aryl and phenyl acetyl hydrazides (19-36) were synthesized and tested for their role as future anticancer agents with less side effects against CA II (major target), CA IX, and CA XII isozymes of carbonic anhydrase. It was found that substitution of the aromatic group has a significant role in determining the structure activity relationship studies. In the case of aryl substation when the 2-OH group was free, an enhanced activity was observed as compared to the compounds having substituted OH group with OMe. In the case of acetyl-substituted sulfonamide-hydrazide hybrids, the addition of CH_2_ group enhances the activity as compared to the aryl group with one carbon. By using BioSolveIT's SeeSAR software, further studies to provide visual clues to binding affinity indicates that the structural elements that are responsible for this were also studied. Among the entire series, only compounds **22**, **32**, and **36** were highly selective inhibitors of hCA IX and did not inhibit hCA XII (<50% inhibition). We wanted to investigate why binding of these compounds to hCA XII is poor. For this purpose, compounds **32** and **36** were docked against both hCA IX and hCA XII. Binding of these compounds with hCA IX was highly favorable (as expected) and in agreement with the experimental data.

## 4. Methodology

### 4.1. General Procedure for the Synthesis of Ester **(6-9)**

Substituted benzoic acid and phenyl acetic acids were refluxed with excess of methanol in the presence of sulfuric acid as catalyst to get methoxy esters **(6-9)** [14, 24].

- Methyl 2-Methoxy-4-methylbenzoate **6**
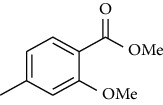
- Yield: 88%; *R*_*f*_  = (*n* − hexane : EtOAc = 6 : 4) 0.6; mp (oil) [24].
- Methyl 2-Hydroxy-4-methylbenzoate **7**
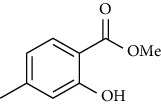
- Yield: 92%; *R*_*f*_  = .(*n* − hexane : EtOAc. = 7 : 3) 0.8; mp (oil) [24].
- Methyl 2-(p-Tolyl)acetate (8)
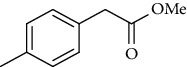
- Yield: 87%; *R*_*f*_ = (*n* − hexane : EtOAc. = 4 : 1) 0.8; bp. (°C); oil [24].
- Methyl 2-(3-Methoxyphenyl)acetate **(9)**
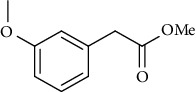
- Yield: 80%; *R*_*f*_ = (*n* − hexane : EtOAc. = 4 : 1) 0.7; bp (°C); oil [24].
- General Procedure for the Synthesis of Hydrazides **(10-13)**

The alcoholic solution of methyl esters **(6-9)** were refluxed with the hydrazine monohydrate for 4-5 hours to get hydrazides **(10-13)**. The solid obtained was purified by recrystallization using appropriate solvent [14, 24].

- 2-Methoxy-4-methylbenzohydrazide **(10)**
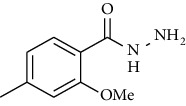
- Yield: 92%; *R*_*f*_  = (*n* − hexane : EtOAc = 3 : 2) 0.2; mp. (109-110°C) [24].
- 2-Hydoxy-4-methylbenzohydrazide **(11)**
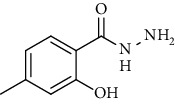
- Yield: 77%; *R*_*f*_  = (*n* − hexane : EtOAc = 3 : 2) 0.2; mp. Melt with decompose [24].
- 2-(*p*-Tolyl)acetohydrazide **(12)**
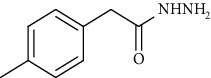
- Yield: 85%; *R*_*f*_ = (*n* − hexane : EtOAc. = 3 : 2); mp. (153-154°C) [24].
- 2-(3-Methoxyphenyl)acetohydrazide **(13)**
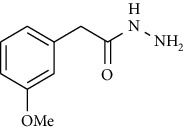
- Yield: 82%; *R*_*f*_ = (*n* − hexane : EtOAc. = 3 : 2) 0.2; mp.(95-96°C) [24].
- General Procedure for the Synthesis of Hydrazide Sulfonamide (**19-36**)

Acyl- and aryl-substituted hydrazides (**10-13**) were stirred at room temperature with substituted sulfonyl chloride (**14-18**) in the presence of pyridine. After overnight stirring, solid product precipitated in the flask which was neutralized with dilute HCl to get rid of pyridine as pyridinium chloride. The solid obtained was filtered, washed with cold water, and recrystallized with appropriate solvent to purify the final product **(19-36)** [15].

- *N*′-(2-Methoxy-4-methylbenzoyl)-4-nitrobenzenesulfonohydrazide **(19)**
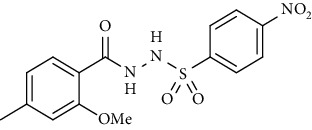
- Yield: 89%; *R*_*f*_ = (*n* − hexane : EtOAc = 3 : 2) 0.5; mp. (220-222°C); HPLC purity = 96.1% (C18 RP, CH_3_CN/H_2_O-1:1), TR = 11.1 min, FT-IR (ῡ, cm^−1^): 3321, 3149, 2817, 1664 (C=O), 1541 (NO_2_), 1403, 1380, 1262, 1181; ^1^H NMR (400 MHz, DMSO-*d_6_*) (*δ*, ppm): 10.46 (s,1H, NH), 10.21 (s, 1H, NH), 8.39 (d, *J* = 8.1 Hz, 2H_Ar_), 8.09 (d, *J* = 8.8 Hz, 2H_Ar_), 7.26 (d, *J* = 7.7 Hz, 1H_Ar_), 6.92 (s, 1H_Ar_), 6.78 (d, *J* = 7.7 Hz, 1H_Ar_), 3.80 (s, 3H, OCH_3_), 2.32 (s, 3H, CH_3_), ^13^C-NMR (100 MHz, DMSO-*d*_6_): *δ* (ppm) 165.0, 156.9, 149.9, 145.2, 143.1, 129.9, 128.9, 125.1, 124.1, 121.0, 118.7, 55.8, 21.3, ESI-MS: C_15_H_15_N_3_O_6_S [M+1] 366.2.
- 4-Bromo-*N*′-(2-methoxy-4-methylbenzoyl)benzenesulfonohydrazide **(20)**
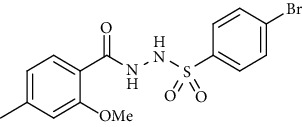
- Yield: 85%; *R*_*f*_  = (*n* − hexane : EtOAc. = 3 : 2) 0.4; mp. (168-169°C); HPLC purity = 91.8% (C18 RP, CH_3_CN/H_2_O-1:1), TR = 10.1 min, FT-IR (ῡ, cm^−1^): 3321, 3095 (aromatic CH), 2887, 1622 (C=O), 1375, 1263, 1198; ^1^H NMR (400 MHz, CDCl_3_) (*δ*, ppm)): 9.56 (s, 2H, NH), 7.77 (dt, *J* = 8.6, 2.3 Hz, 2H_Ar_), 7.72 (d, *J* = 7.9 Hz, 1H_Ar_), 7.59 (dt, *J* = 8.6, 2.3 Hz, 2H_Ar_), 6.86 (d, *J* = 8.0 Hz, 1H_Ar_), 6.81 (s, 1H_Ar_), 4.03 (s, 3H, OCH_3_), 2.41 (s, 3H, CH_3_), ^13^C NMR (100 MHz, CDCl_3_) (*δ*, ppm), 164.3, 157.6, 145.6, 135.7, 132.3, 130.1, 129.0, 122.6, 115.4, 112.2, 56.3, 22.0.
- 4-Methoxy-*N*′-(2-methoxy-4-methylbenzoyl)benzenesulfono hydrazide **(21)**
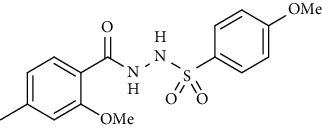
- Yield: 83%; *R*_*f*_ = (*n* − hexane : EtOAc = 3 : 2) 0.3; mp (159-162°C); FT-IR (ῡ, cm^−1^): 3281, 3142 (aromatic CH), 2925, 1696 ((C=O), 1358, 1234, 1184; ^1^H NMR (400 MHz, CDCl_3_) (*δ*, ppm): 9.56 (s, 2H, NH), 7.83 (d, *J* = 8.8 Hz, 2H_Ar_), 7.72 (d, *J* = 7.9 Hz, 1H_Ar_), 6.90 (d, *J* = 8.8 Hz, 2H_Ar_), 6.83 (d, *J* = 8.1 Hz, 1H_Ar_), 6.80 (s, 1H_Ar_), 4.02 (s, 3H, OCH_3_), 3.83 (s, 3H, OCH_3_), 2.40 (s, 3H, CH_3_) ^13^C NMR (100 MHz, CDCl_3_) (*δ*, ppm), 166.1, 156.6, 144.3, 134.8, 133.1, 129.8, 129.5, 121.4, 115.9, 112.8, 56.3, 54.6, 21.9.
- *N*′-(2-Methoxy-4-methylbenzoyl)-4-methylbenzenesulfonohydrazide **(22)**
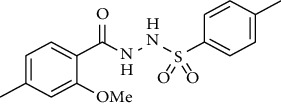
- Yield: 76%; *R*_*f*_ = (*n* − hexane : EtOAc = 3 : 2) 0.4; mp. (161-163°C); FT-IR (ῡ, cm^−1^): 3361, 3137 (aromatic CH), 2861, 1679 (C=O), 1332, 1221, 1178; ^1^H NMR (400 MHz, CDCl_3_) (*δ*, ppm): 7.87 (d, *J* = 8.2 Hz, 2H_Ar_), 7.40 (d, *J* = 7.4 Hz, 2H_Ar_), 7.37 (d, *J* = 8.1 Hz, 1H_Ar_), 6.78 (s, 1H_Ar_), 6.75 (d, *J* = 8.1 Hz, 1H_Ar_), 3.88 (s, 3H, OCH_3_), 2.44 (s, 3H, CH_3_), 2.30 (s, 3H, CH_3_).
- *N*′-(2-Methoxy-4-methylbenzoyl)naphthalene-2-sulfonohydrazide **(23)**
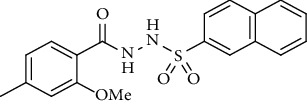
- Yield: 79%; *R*_*f*_ = (*n* − hexane : EtOAc = 3 : 2) 0.4; mp. (186-187°C); HPLC purity = 97.42% (C18 RP, CH_3_CN/H_2_O-1:1), TR = 10.1 min, FT-IR (ῡ, cm^−1^): 3370, 3055 (aromatic CH), 2805, 1640 (C=O), 1360, 1290, 1196, 1187; ^1^H NMR (400 MHz, CDCl_3_) (*δ*, ppm): 9.61 (s, 2H, NH), 8.48 (s, 1H_Ar_), 7.87-7.91 (m, 4H_Ar_), 7.63 (td, *J* = 7.0, 0.9 Hz, 1H_Ar_), 7.54-7.58 (m, 2H_Ar_), 6.78 (s, 1H_Ar_), 6.74 (d, *J* = 8.0 Hz, 1H_Ar_), 4.01 (s, 3H, OCH_3_), 2.37 (s, 3H, CH_3_), ESI-MS: C_19_H_18_N_2_O_4_S [M+1] 371.2.
- *N*′-(2-Hydroxy-4-methylbenzoyl)-4-nitrobenzenesulfonohydrazide **(24)**
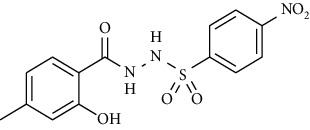
- Yield: 82%; *R*_*f*_ = (*n* − hexane : EtOAc = 3 : 2) 0.2; mp. (211-213°C); FT-IR (ῡ, cm^−1^): 3365, 3132, 2805, 1670 (C=N), 1553 (NO_2_), 1376 (nitro), 1387, 1230, 1189; ^1^H NMR (400 MHz, DMSO-*d_6_*) (*δ*, ppm): 11.29 (s, 1H, OH), 10.61 (s, 1H, NH), 10.52 (s, 1H, NH), 8.39 (d, *J* = 8.8 Hz, 2H_Ar_), 8.08 (d, *J* = 8.8 Hz, 2H_Ar_), 7.58 (d, *J* = 8.2 Hz, 1H_Ar_), 6.71 (d, *J* = 7.6 Hz, 1H_Ar_), 6.71 (s, 1H_Ar_), 2.25 (s, 3H, CH_3_).
- 4-Bromo-*N*′-(2-hydroxy-4-methylbenzoyl)benzenesulfonohydrazide **(25)**
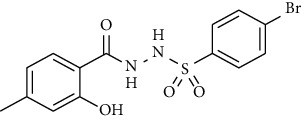
- Yield: 81%; *R*_*f*_ = (*n* − hexane : EtOAc = 3 : 2) 0.3; mp (231-215°C); FT-IR (ῡ, cm^−1^): 3357, 3087, 2809, 1656 (C=O), 1368, 1271, 1192; ^1^H NMR (400 MHz, CDCl_3_) (*δ*, ppm): 10.50 (bs, 1H, OH), 7.78 (d, *J* = 7.6 Hz, 2H_Ar_), 7.62 (d, *J* = 8.5 Hz, 2H_Ar_), 7.33 (d, *J* = 8.1 Hz, 1H_Ar_), 6.78 (s, 1H_Ar_), 6.72 (d, *J* =8.0 Hz, 1H_Ar_), 2.19 (s, 3H, CH_3_).
- *N*′-(2-Hydroxy-4-methylbenzoyl)-4-methoxybenzenesulfono hydrazide **(26)**
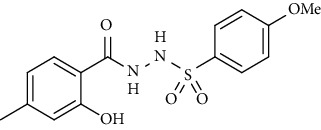
- Yield: 75%; *R*_*f*_ = .(*n* − hexane : EtOAc = 3 : 2) 0.2; mp (168-169°C); FT-IR (ῡ, cm^−1^): 3343, 3028, 2865, 1663 (C=O), 1362, 1199, 1187; ^1^H NMR (400 MHz, CDCl_3_) (*δ*, ppm): 10.55 (bs, 1H, OH), 7.85 (d, *J* = 8.5 Hz, 2H_Ar_), 7.74 (d, *J* = 7.9 Hz, 1H_Ar_), 6.93 (d, *J* = 8.5 Hz, 2H_Ar_), 6.87 (d, *J* = 8.1 Hz, 1H_Ar_), 6.79 (s, 1H_Ar_), 3.86 (s, 3H, OCH_3_), 2.42 (s, 3H, CH_3_).
- *N*′-(2-Hydroxy-4-methylbenzoyl)-4-methylbenzenesulfonohydrazide **(27)**
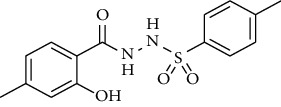
- Yield: 85%; *R*_*f*_  = (*n* − hexane : EtOAc = 3 : 2) 0.3; mp. (183-185°C); FT-IR (ῡ, cm^−1^): 3389, 3012 (aromatic CH), 2896, 1671 (C=O), 1336, 1231, 1182; ^1^H NMR (400 MHz, CDCl_3_) (*δ*, ppm): 10.56 (bs, 1H, OH), 7.81 (d, *J* = 8.2 Hz, 2H_Ar_), 7.43 (d, *J* = 7.4 Hz, 2H_Ar_), 7.32 (d, *J* = 8.1 Hz, 1H_Ar_), 6.76 (s, 1H_Ar_), 6.73 (d, *J* = 8.2 Hz, 1H_Ar_), 2.42 (s, 3H, CH_3_), 2.34 (s, 3H, CH_3_), ESI-MS: C_15_H_16_N_2_O_4_S [M+1] 321.2.
- 4-Nitro*-N*′-(2-(*p*-tolyl)acetyl)benzenesulfonohydrazide **(28)**
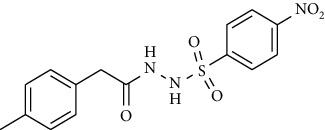
- Yield: 85%; *R*_*f*_. = (*n* − hexane : EtOAc. = 1 : 1) 0.5; mp. (205-206°C); FT-IR (ῡ, cm^−1^): 3375, 3018 (aromatic CH), 2912, 1698 (C=O), 1550 (nitro asymmetric), 1385 (nitro symmetric), 1324, 1195; ^1^H NMR (400 MHz, DMSO-*d_6_*) (*δ*, ppm): 10.45 (s, 1H, NH), 10.33 (s, 1H, NH), 8.18 (d, *J* = 8.8 Hz, 2H_Ar_), 7.90 (d, *J* = 8.8 Hz, 2H_Ar_), 7.06 (d, *J* = 7.6 Hz, 2H_Ar_), 6.97 (d, *J* = 8.0 Hz, 2H_Ar_), 3.22 (s, 2H, CH_2_), 2.28 (s, 3H, CH_3_).
- 4-Bromo-*N*′-(2-(*p*-tolyl)acetyl)benzenesulfonohydrazide **(29)**
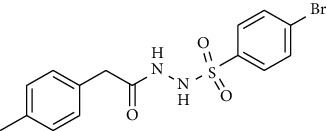
- Yield: 70%; *R*_*f*_. = .(*n* − hexane : EtOAc. = 1 : 1) 0.5; mp. (156-157°C); FT-IR (ῡ, cm^−1^): 3360, 3022 (aromatic CH), 2918, 1696 (C=O), 1328, 1195; ^1^H NMR (400 Hz, CDCl_3_) (*δ*, ppm): 7.99 (d, *J* = 7.8 Hz, 2H_Ar_), 7.62 (d, *J* = 8.4 Hz, 2H_Ar_), 7.50 (d, *J* = 8.4 Hz, 2H_Ar_), 7.17 (d, *J* = 7.7 Hz, 2H_Ar_), 3.37 (s, 2H, CH_2_), 2.40 (s, 3H, CH_3_).
- 4-Methoxy-*N*′-(2-(*p*-tolyl)acetyl)benzenesulfonohydrazide (**30)**
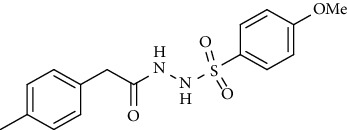
- Yield: 82%; *R*_*f*_. = (*n* − hexane : EtOAc. = 1 : 1) 0.3; mp. (133-134°C); FT-IR (ῡ, cm^−1^): 3354 (NH), 3043, 2919, 1697 (C=O), 1327, 1191; ^1^H NMR (400 MHz, CDCl_3_) (*δ*, ppm): 7.73 (d, *J* = 8.8 Hz, 2H_Ar_), 7.15 (d, *J* = 7.7 Hz, 2H_Ar_), 7.00 (d, *J* = 7.8 Hz, 2H_Ar_), 6.89 (d, *J* = 8.8 Hz, 2H_Ar_), 3.87 (s, 3H, OCH_3_), 3.37 (s, 2H, CH_2_), 2.37(s, 3H, CH_3_).
- 4-Methyl-*N*′-(2-(*p-*tolyl)acetyl)benzenesulfonohydrazide **(31)**
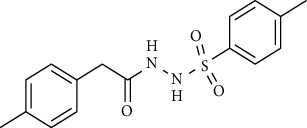
- Yield: 80%; *R*_*f*_. = (*n* − hexane : EtOAc. = 1 : 1) 0.4; mp. (143-144°C); FT-IR (ῡ, cm^−1^): 3343 (NH), 3043, 2965, 1692 (C=O), 1328, 1197; ^1^H NMR (400 MHz, CDCl_3_) (*δ*, ppm): 7.69 (d, *J* = 8.2 Hz, 2H_Ar_), 7.22 (d, *J* = 8.0 Hz, 2H_Ar_), 7.15 (d, *J* = 7.7 Hz, 2H_Ar_), 7.00 (d, *J* = 7. Hz, 2H_Ar_), 3.36 (s, 2H, CH_2_), 2.42 (s, 3H, CH_3_), 2.38 (s, 3H, CH_3_).
- *N*′-(2-(3-Methoxyphenyl)acetyl)-4-nitrobenzenesulfonohydrazide **(32)**
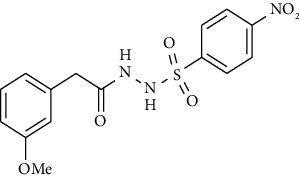
- Yield: 76%; *R*_*f*_. = (*n* − hexane : EtOAc. = 1 : 1) 0.4; mp. (158-159°C); FT-IR (ῡ, cm^−1^): 3360 (NH), 3018, 2912, 1685 (C=O), 1553 (nitro asymmetric), 1390 (nitro symmetric), 1326, 1195, 1282; ^1^H NMR (400 MHz, DMSO-*d_6_*) (*δ*, ppm): 10.47 (d, 1H, NH), 10.36 (d, 1H, NH), 8.21 (d, *J* = 8.8 Hz, 2H_Ar_), 7.90 (d, *J* = 8.8 Hz, 2H_Ar_), 7.18 (t, *J* = 7.8 Hz, 1H_Ar_), 6.80 (dd, *J* = 8.1, 2.2 Hz, 1H_Ar_), 6.69 (d, *J* = 7.5 Hz, 1H_Ar_), 6.64 (s, 1H_Ar_), 3.71 (s, 3H, OCH_3_), 3.26 (s, 2H, CH_2_).
- 4-Bromo-*N*′-(2-(3-methoxyphenyl)acetyl)benzenesulfonohydrazide **(33)**
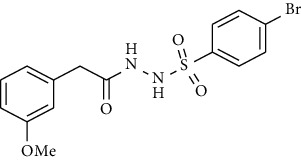
- Yield: 80%; *R*_*f*_. = (*n* − hexane : EtOAc. = 1 : 1) 0.4; mp. (153-154°C); FT-IR (ῡ, cm^−1^): 3345 (NH), 3018, 2932, 1697 (C=O), 1345, 1282, 1195; ^1^H NMR (400 MHz, CDCl_3_) (*δ*, ppm): 7.63 (t, *J* = 8.4, 1H_Ar_) 7.62 (d, *J* = 8.4 Hz, 2H_Ar_), 7.53 (d, *J* = 8.4, 2H_Ar_), 6.90 (dd, *J* = 8.2, 1.8 Hz, 1H_Ar_), 6.70 (d, *J* = 7.16 Hz, 1H_Ar_), 6.66 (s, 1H_Ar_), 3.84 (s, 3H, OCH_3_), 3.38 (s, 2H, CH_2_).
- 4-Methoxy-*N*′-(2-(3-methoxyphenyl)acetyl)benzenesulfonohydrazide **(34)**
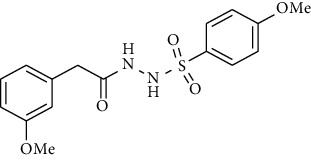
- Yield: 70%; *R*_*f*_. = (*n* − hexane : EtOAc. = 1 : 1) 0.2; mp. (146-147°C); FT-IR (ῡ, cm^−1^): 3364 (NH), 3054, 2910, 1698 (C=O), 1356, 1195; ^1^H NMR (400 MHz, CDCl_3_) (*δ*, ppm): 9.85 (bs, 2H, NH), 7.71-7.75 (m, 2H_Ar_), 6.84-6.90 (m, 4H_Ar_), 6.71 (d, *J* = 6.1 Hz, 1H_Ar_), 6.67 (s, 1H_Ar_), 3.88 (s, 3H, OCH_3_), 3.82 (s, 3H, OCH_3_), 3.38 (s, 2H, CH_2_).
- *N*′-(2-(3-Methoxyphenyl)acetyl)-4-methylbenzenesulfonohydrazide **(35)**
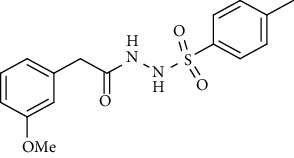
- Yield: 87%; *R*_*f*_. = (*n* − hexane : EtOAc. = 1 : 1) 0.3; mp. (143-144°C); FT-IR (ῡ, cm^−1^): 3342 (NH), 3034, 2912, 1697 (C=O), 1388, 1196, 1282; ^1^H NMR (400 MHz, CDCl_3_) (*δ*, ppm): 7.79 (d, *J* = 7.6 Hz, 2H_Ar_), 7.68 (d, *J* = 7.6 Hz, 2H_Ar_), 7.23 (t, *J* = 7.6 Hz, 1H_Ar_), 6.87 (d, *J* = 7.6 Hz, 2H_Ar_), 6.70 (d, *J* = 6.8 Hz, 2H_Ar_), 6.67 (s, 1H_Ar_), 3.82 (s, 3H, OCH_3_), 3.37 (s, 2H, CH_2_) 2.42 (s, 3H, CH_3_).
- *N*′-(2-(3-Methoxyphenyl)acetyl)naphthalene-2-sulfonohydrazide **(36)**
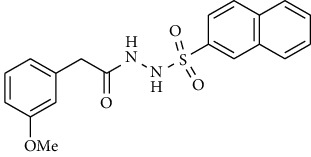
- Yield: 84%; *R*_*f*_. = (*n* − hexane : EtOAc. = 1 : 1) 0.5; mp. (139-141°C); FT-IR (ῡ, cm^−1^): 3358 (NH), 3018, 2912, 1695 (C=O), 1352, 1189, 1282; ^1^H NMR (400 MHz, CDCl_3_) (*δ*, ppm): 8.41 (s, 1H_Ar_), 7.89-7.95 (m, 3H_Ar_), 7.80 (dd, *J* = 8.7, 1.7 Hz, 1H_Ar_), 7.62-7.71 (m, 2H_Ar_), 7.39 (d, *J* = 6.0 Hz, 1H_Ar_), 7.15 (t, *J* = 7.7 Hz, 1H_Ar_), 6.81 (dd, *J* = 8.2, 1.8 Hz, 1H_Ar_), 6.59 (s, 1H_Ar_), 3.75 (s, 3H, OCH_3_), 3.31 (s, 2H, CH_2_).

## 5. Protein Expression

All three isozymes, CA II, CA IX, and CA XII, were produced using the same protocol done for each. Transformation was the first step to be done in which the plasmids and the off-spark plasmids of CA II, CA IX, and CA XII were transformed into competent *E. coli* bacteria using the heat shock method, cells were grown in agar, then a colony was selected for further growth in LB broth, cells were collected by centrifugation according to the methods described in [25], and the plasmid was extracted according to the kit protocol using SanPrep column plasmid miniprep kit obtained from Sangon Biotech. The following step was the growing of the HEK-293 cells in DMEM supplemented with 10% fetal bovine serum (FBS) and 1% pen/strep mixture, the transfection procedure was conducted seeding the cells in a 96-well plate and addition of the mixture of the plasmid (0.1 *μ*g) and Lipofectamine 3000 (0.2 *μ*L), incubating the mixture at 37°C and 5% CO_2_ [26]. Six hours later, the media were aspirated, and the fresh prepared (+10% FBS and 1% pen/strep) media were added to the cells. On the next day, the cells were inspected for their viability, and new media were used for the selection process which were DMEM, FBS, and hygromycin B using a final concentration of 200 *μ*g/mL. The cells were grown in the selective media, and when they reached 70 to 80% confluency, the offs-park plasmid transfected cells were checked under a fluorescent microscope to insure the production of the protein; then, the cells transfected with the His-tagged plasmid were collected for making the cell lysate following the method described by [27] by centrifugation of the cells at 2000 rpm for 5 minutes, washed with PBS, and again centrifuged; the pellet was then suspended in the cell lysis buffer, incubated in ice for 15 minutes, and centrifuged again at 10000 g at 4°C for 15 minutes, and here, the pellet was discarded and the supernatant was collected for purification using Ni-NTA Sepharose column according to the manufacturer's protocol [28]. And the eluting solution was quantified using the protein quantification technique Biuret assay and Bradford reagent for protein quantification.

## 6. Carbonic Anhydrase Inhibition Assay

Carbonic anhydrase inhibition activity was carried out using an already developed method [19] with slight modification. The principle of the current method is centered on that “CA hydrolyses the *p*-nitrophenyl acetate to *p*-nitrophenol” which is determined by spectrophotometrically. Reaction mixture contained 60 *μ*L of 50 mM Tris-sulfate buffer (pH 7.6 containing 0.1 mM ZnCl2) and 10 *μ*L (0.5 mM) test compound in 1% DMSO. All the ingredients were blended and preincubated for 10 min at 25°C. The 96-well plate reader was used to preread the plates at 348 nm. Preparation of *p*-nitrophenyl acetate was done by taking 6 mM stock using <5% acetonitrile in buffer and was used fresh. Each well was filled with 20 *μ*L solution to attain 0.6 mM concentration. The total reaction volume was made to 100 *μ*L. After 30 minutes of incubation at 37°C, all ingredients were blended and reading was taken at 348 nm. Acetazolamide was used as the standard while DMSO was used as positive controls. The results reported are mean of the three independent experiments (±SEM) and expressed as percent inhibitions calculated by the formula,

(1) $$\begin{array}{c} \text{Inhibition }\left(\%\right)=\left[100-\left(\text{Abs of test comp}/\text{Abs of control}\right)\text{x}100\right]. \end{array}$$

The IC_50_ values of selected compounds exhibiting >50% activity at 1.0 mM were calculated after suitable dilutions [19].

## 7. Molecular Docking Studies

Molecular docking study of the most potent inhibitors **24**, **23**, and **30** was carried out in CA II (PDB ID **3k34**), CA IX (PDB ID **5FL4**), and CA XII (PDB ID **5MSA**), respectively [2–29]. FlexX utility of BioSolveIT's LeadIT program was used for molecular docking [30]. Default parameters of the protein preparation were used to prepare the receptor for docking. The cocrystallized inhibitors inside the carbonic anhydrase isozymes obtained from the protein databank were selected as reference ligand, and the protocol validation was carried out by redocking the cocrystallized inhibitors. After validation of the docking protocol, actual docking of the potent inhibitors was carried out. Hybrid enthalpy and entropy approach of FlexX docking was used for scoring and ranking of the docking poses. The top-ranking poses were then subjected to HYDE assessment and selection of the possible binding mode [31–33].

## Figures and Tables

**Table 1:** Physical data of hydrazide **(10-13)**.

Sr. no.	Structure	mp (°C)	*R*_*f*_	Yield (%)
**10**	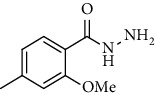	109-110	0.2	92
**11**	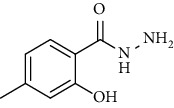	>300	0.2	77
**12**	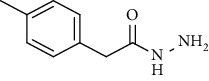	153-154	0.2	85
**13**	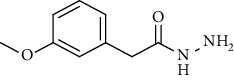	95-96	0.2	82

**Table 2:** Physical data of sulfonamides (19-36).

Sr. no.	Structure	mp (°C)	*R*_*f*_	Yield (%)
**19**	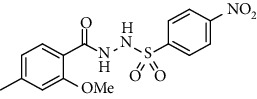	220-222	0.5	89
**20**	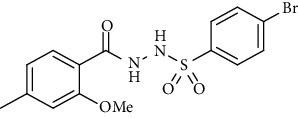	168-169	0.4	85
**21**	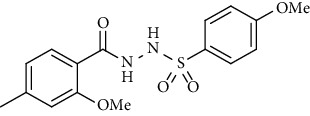	159-162	0.3	83
**22**	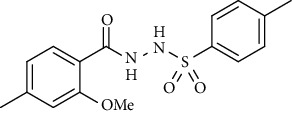	161-163	0.4	76
**23**	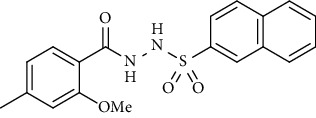	186-187	0.4	79
**24**	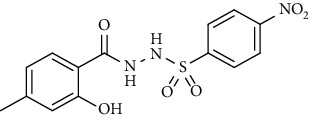	211-213	0.2	82
**25**	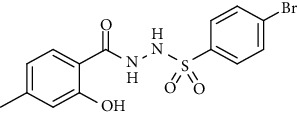	231-215	0.3	81
**26**	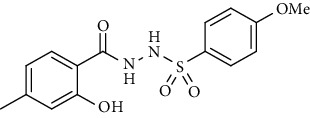	168-169	0.2	75
**27**	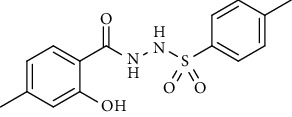	183-185	0.3	85
**28**	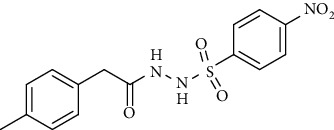	205-206	0.5	85
**29**	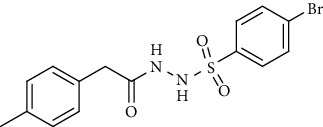	156-157	0.5	70
**30**	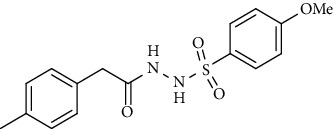	133-134	0.3	82
**31**	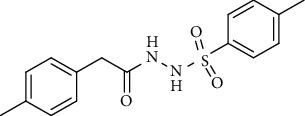	143-144	0.4	80
**32**	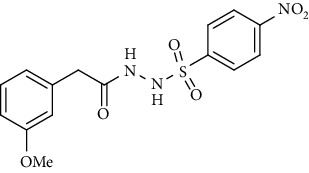	158-159	0.4	76
**33**	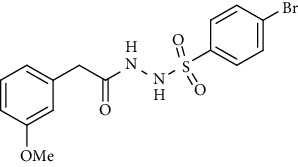	153-154	0.4	80
**34**	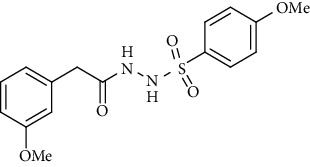	146-147	0.2	70
**35**	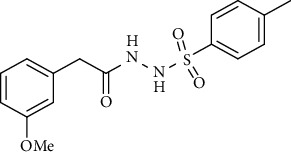	143-144	0.3	87
**36**	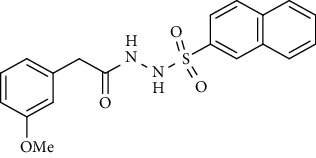	139-141	0.5	84

**Table 3:** Comparison of experimental and computed NMR data for compound 19.

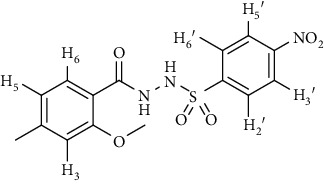				
Compound **19**				
Carbon no.	Carbon type	^1^H-NMR (experimental) *δ* (ppm)	^1^H-NMR (computed) *δ* (ppm)	Δ*δ* (ppm)
3	CH	6.92	7.06	-0.14
5	CH	6.78	7.08	-0.3
6	CH	7.26	8.42	-1.16
2′	CH	8.39	8.22	0.17
3′	CH	8.09	8.56	-0.47
5′	CH	8.09	8.7	-0.61
6′	CH	8.39	10.01	-1.62
4-Me	CH_3_	2.32	2.24	0.08
2-OMe	CH_3_	3.80	3.74	0.06
Mean absolute error (MAE) = 0.19 Root mean square error (RMSE) = 0.44				

**Table 4:** Ki values and inhibition percentages of the synthesized compounds against CA II, CA IX, and CA XII.

Sr no.	CAII	CAIX	CAXII
	Ki (*μ*M ± SEM)/% inhibition		
19	0.68 ± 0.02	0.58 ± 0.04	44.4%
20	0.66 ± 0.04	0.56 ± 0.05	0.99 ± 0.10
**21**	0.61 ± 0.01	0.19 ± 0.02	1.22 ± 0.01
22	0.68 ± 0.01	1.04 ± 0.38	42.89%
**23**	0.94 ± 0.02	0.19 ± 0.03	0.75 ± 0.07
24	0.34 ± 0.01	0.54 ± 0.06	0.52 ± 0.04
25	0.91 ± 0.02	0.33 ± 0.03	0.35 ± 0.04
26	0.96 ± 0.09	0.91 ± 0.09	0.58 ± 0.04
27	1.11 ± 0.04	0.51 ± 0.04	0.43 ± 0.08
28	0.46 ± 0.01	0.55 ± 0.01	1.02 ± 0.08
29	0.73 ± 0.02	0.33 ± 0.02	0.55 ± 0.01
**30**	0.74 ± 0.04	0.33 ± 0.03	0.13 ± 0.01
**31**	0.67 ± 0.01	0.29 ± 0.02	0.40 ± 0.09
**32**	0.67 ± 0.06	0.32 ± 0.01	49.25%
**33**	0.68 ± 0.05	0.39 ± 0.03	0.17 ± 0.01
**34**	1.81 ± 0.03	0.65 ± 0.03	0.22 ± 0.06
35	3.20 ± 0.14	1.18 ± 0.04	0.34 ± 0.01
36	0.92 ± 0.10	0.60 ± 0.01	48.88%
Acetazolamide	0.31 ± 0.03	0.30 ± 0.01	0.20 ± 0.02

## Data Availability

The data is already entered in the manuscript. A series of sulfonamide-hydrazide hybrids of aryl and phenyl acetyl hydrazides (19-36) were synthesized and tested for their role as future anticancer agents with less side effects against CA II (major target), CA IX, and CA XII isozymes of the carbonic anhydrase. It was found that substitution of the aromatic group has a significant role in determining the structure activity relationship studies. In the case of aryl substation, when 2 OH group was free, an enhanced activity was observed as compared to the compounds having substituted OH group with OMe. In the case of acetyl-substituted sulfonamide-hydrazide hybrids, the addition of CH2 group enhances the activity as compared to the aryl group with one carbon. By using BioSolveIT's SeeSAR software, further studies to provide visual clues to binding affinity indicates that the structural elements that are responsible for this were also studied. Among the entire series, only the compounds 22, 32, and 36 were highly selective inhibitors of hCA IX and did not inhibit hCA XII. We wanted to investigate why binding of these compounds to hCA XII is poor. For this purpose, the compounds 32 and 36 were docked against both hCA IX and hCA XII. Binding of these compounds with hCA IX was highly favorable (as expected) and in agreement with the experimental data.

## Supplementary Materials

Supplementary Materials The supplementary material files contain the ^1^HNMR spectra and DFT tables of all the hydrazide-sulfonamide hybrids while ^13^CNMR, EIMS spectra, and HPLC graphs of the selected compounds.
