# Supplementary material for: Synthesis, Carbonic Anhydrase II/IX/XII Inhibition, DFT, and Molecular Docking Studies of Hydrazide-Sulfonamide Hybrids of 4-Methylsalicyl- and Acyl-Substituted Hydrazide
Source: Biomed Res Int. 2022 Feb 24;2022:5293349. doi: 10.1155/2022/5293349 (PMC8894010; doi:10.1155/2022/5293349)
Supplement: Supplementary Materials — The supplementary material files contain the 1HNMR spectra and DFT tables of all the hydrazide-sulfonamide hybrids while 13CNMR, EIMS spectra, and HPLC graphs of the selected compounds. [file 5293349.f1.zip › All Spectras in one file.pdf]

AKC-25/Adil Khushal/Dr Amara/CDCl3

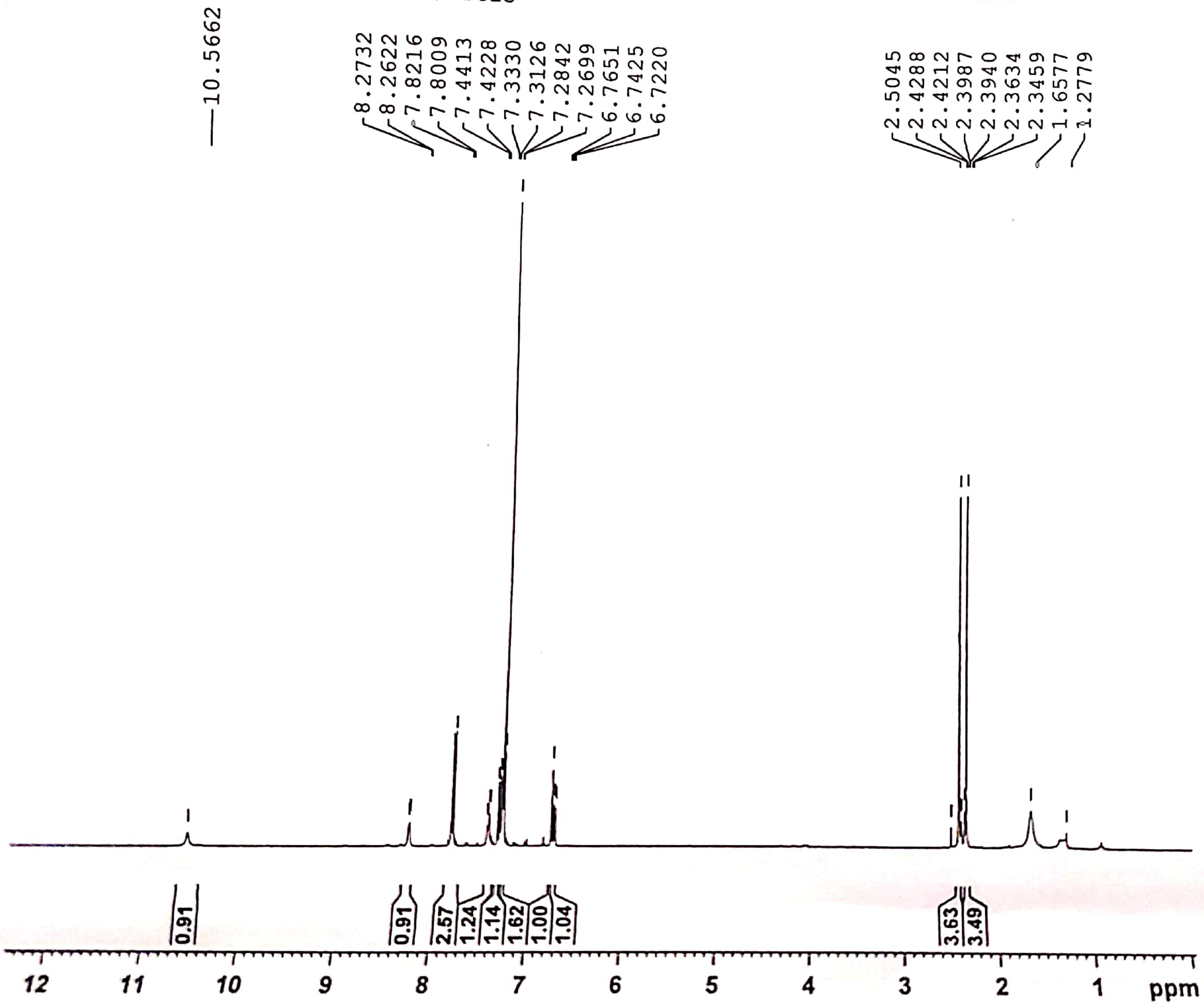

BRUKER

Current Data Parameters	
NAME	Dr. Amara
EXPNO	61
PROCNO	1
F2 - Acquisition Parameters	
Date_	20190615
Time_	11.34 h
INSTRUM	spect
PROBHD	Z116098_0621 (
PULPROG	zg30
TD	65536
SOLVENT	CDCl3
NS	16
DS	2
SWH	8012.820 Hz
FIDRES	0.244532 Hz
AQ	4.0894465 sec
RG	143.62
DW	62.400 usec
DE	6.50 usec
TE	298.0 K
D1	1.00000000 sec
TD0	1
SFO1	400.1324708 MHz
NUC1	1H
P1	10.00 usec
PLW1	16.68099976 W
F2 - Processing parameters	
SI	65536
SF	400.1300000 MHz
WDW	EM
SSB	0
LB	0.30 Hz
GB	0
PC	1.00

Scanned with CamScanner

AKC-25/Adil Khushal/Dr Amara/CDCl3

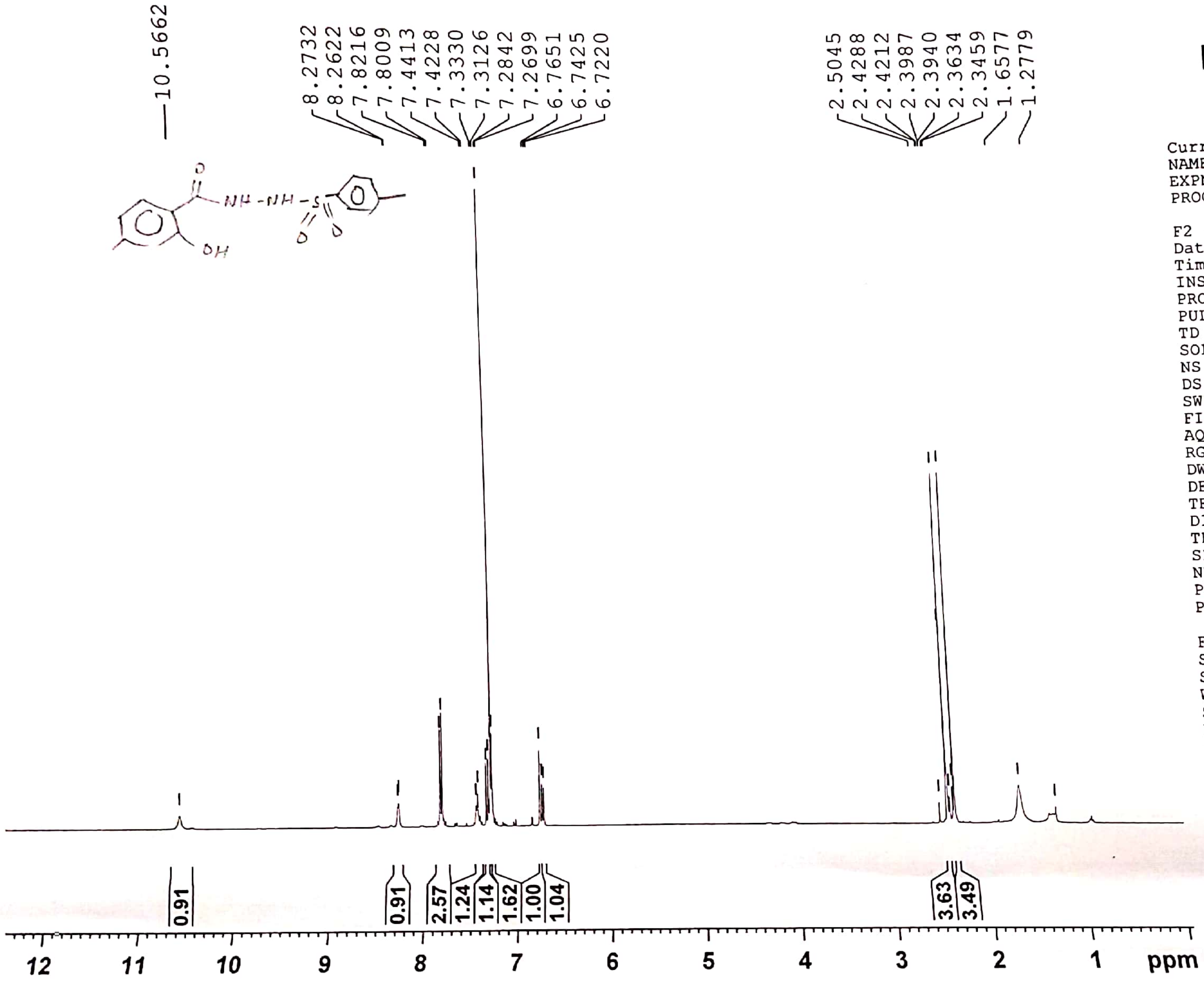

BRUKER

Current Data Parameters

NAME	Dr. Amara
EXPNO	61
PROCNO	1

F2 - Acquisition Parameters

Date_	20190615
Time	11.34 h
INSTRUM	spect
PROBHD	Z116098_0621 (
PULPROG	zg30
TD	65536
SOLVENT	CDCl3
NS	16
DS	2
SWH	8012.820 Hz
FIDRES	0.244532 Hz
AQ	4.0894465 sec
RG	143.62
DW	62.400 usec
DE	6.50 usec
TE	298.0 K
D1	1.00000000 sec
TD0	1
SFO1	400.1324708 MHz
NUC1	1H
P1	10.00 usec
PLW1	16.68099976 W

F2 - Processing parameters

SI	65536
SF	400.1300000 MHz
WDW	EM
SSB	0
LB	0.30 Hz
GB	0
PC	1.00

Scanned with CamScanner

AKC-19/Adil Khushal/Dr. Amara/CDCl3

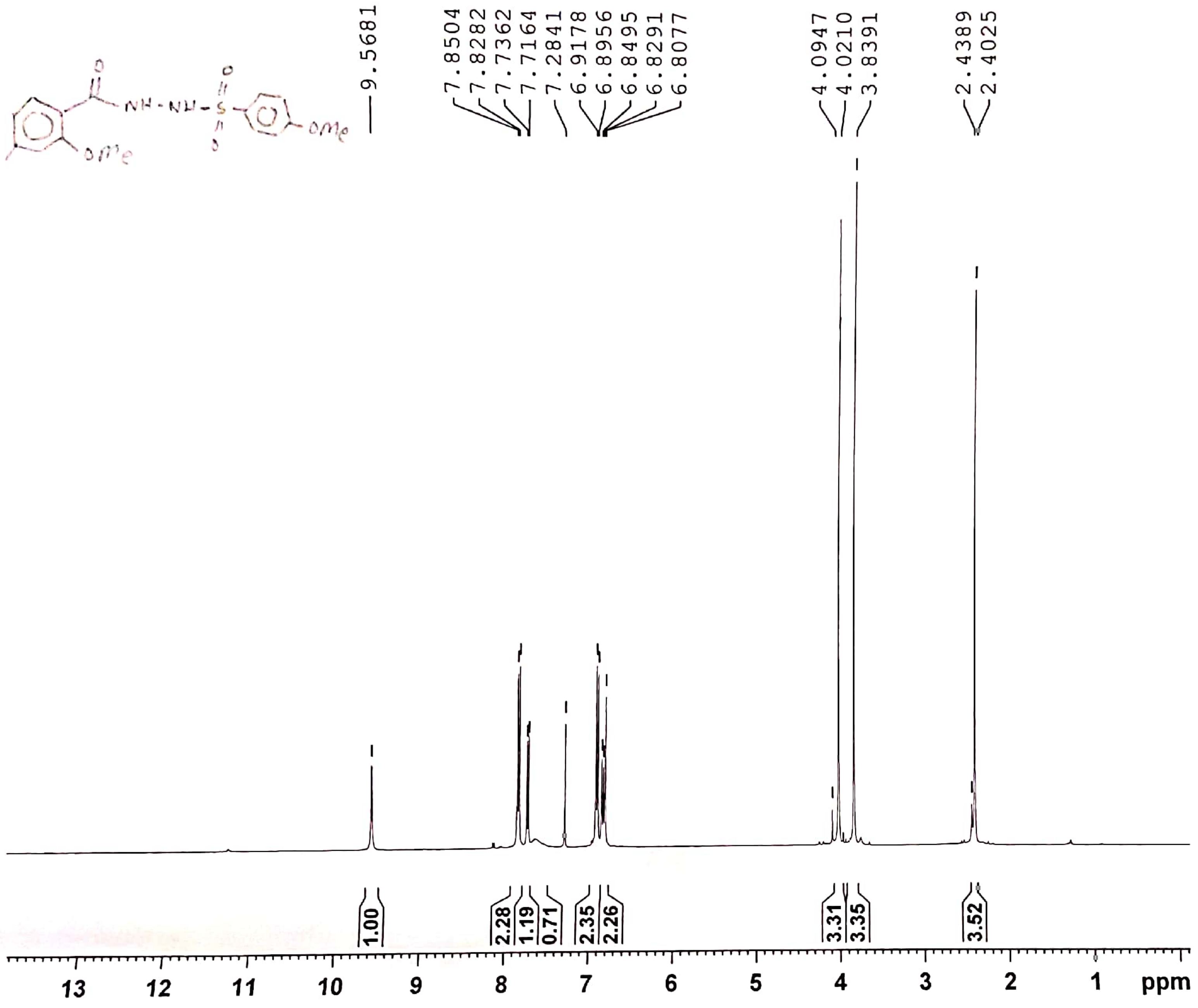

BRUKER

Current Data Parameters

NAME	Dr. Amara	
EXPNO	65	
PROCNO	1	

F2 - Acquisition Parameters

Date_	20190621	
Time	18.27	h
INSTRUM	spect	
PROBHD	Z116098_0621 (	
PULPROG	zg30	
TD	65536	
SOLVENT	CDCl3	
NS	16	
DS	2	
SWH	8012.820	Hz
FIDRES	0.244532	Hz
AQ	4.0894465	sec
RG	88.48	
DW	62.400	usec
DE	6.50	usec
TE	298.0	K
D1	1.00000000	sec
TD0	1	
SFO1	400.1324708	MHz
NUC1	1H	
P1	10.00	usec
PLW1	16.68099976	W

F2 - Processing parameters

SI	65536	
SF	400.1300000	MHz
WDW	EM	
SSB	0	
LB	0.30	Hz
GB	0	
PC	1.00	

Scanned with CamScanner

AKC-23/Adil Khushal/Dr. Amara/CDCl3

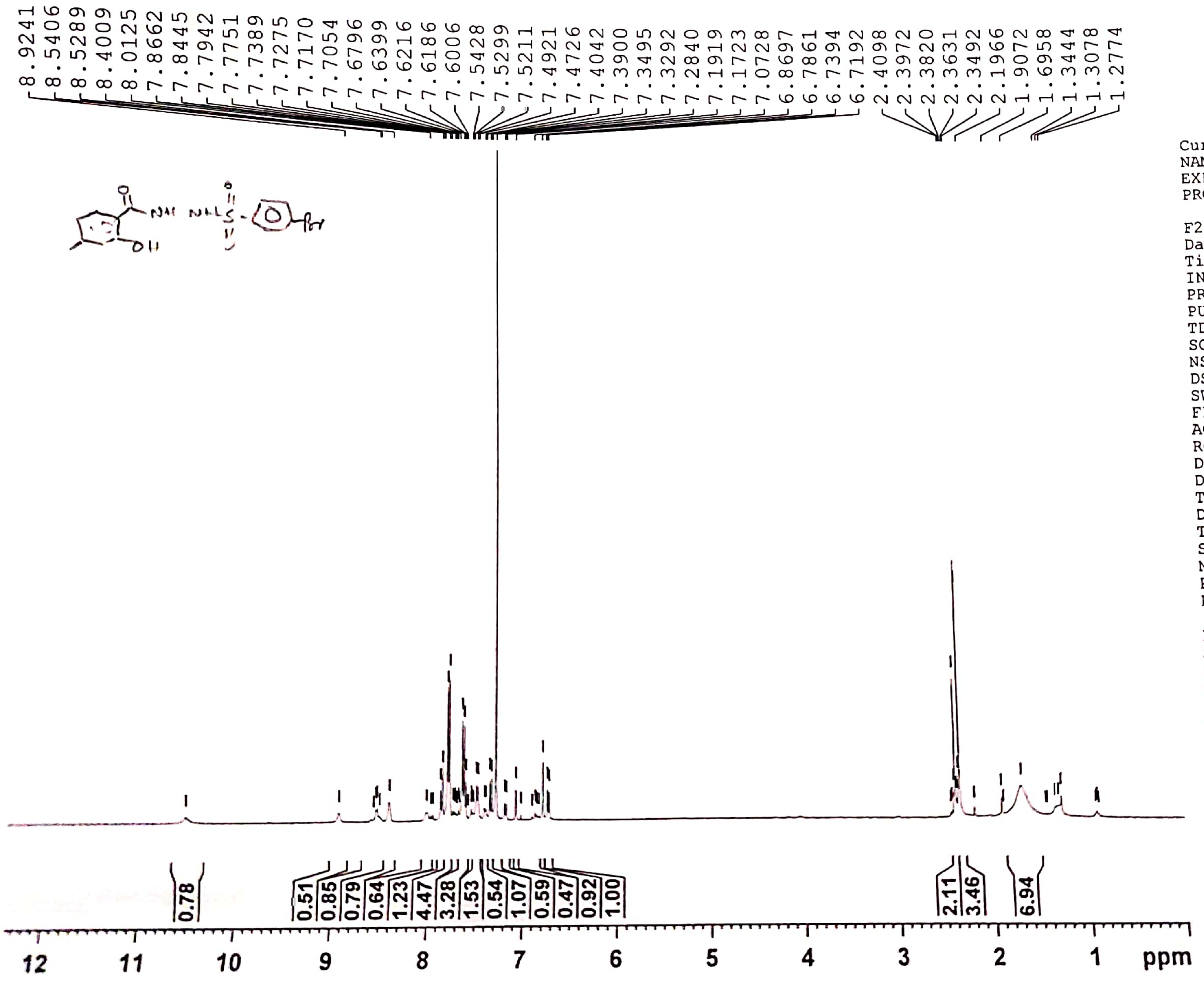

BRUKER

Current Data Parameters
NAME Dr. Amara
EXPNO 66
PROCNO 1

F2 - Acquisition Parameters
Date_ 20190621
Time_ 18.32 h
INSTRUM spect
PROBHD Z116098_0621 (
PULPROG zg30
TD 65536
SOLVENT CDCl3
NS 16
DS 2
SWH 8012.820 Hz
FIDRES 0.244532 Hz
AQ 4.0894465 sec
RG 127.9
DW 62.400 usec
DE 6.50 usec
TE 298.0 K
D1 1.00000000 sec
TD0 1
SFO1 400.1324708 MHz
NUC1 1H
P1 10.00 usec
PLW1 16.68099976 W

F2 - Processing parameters
SI 65536
SF 400.1300000 MHz
WDW EM
SSB 0
LB 0.30 Hz
GB 0
PC 1.00

Scanned with CamScanner

AKC-20/Adil Khushal/Dr. Amara/DMSO

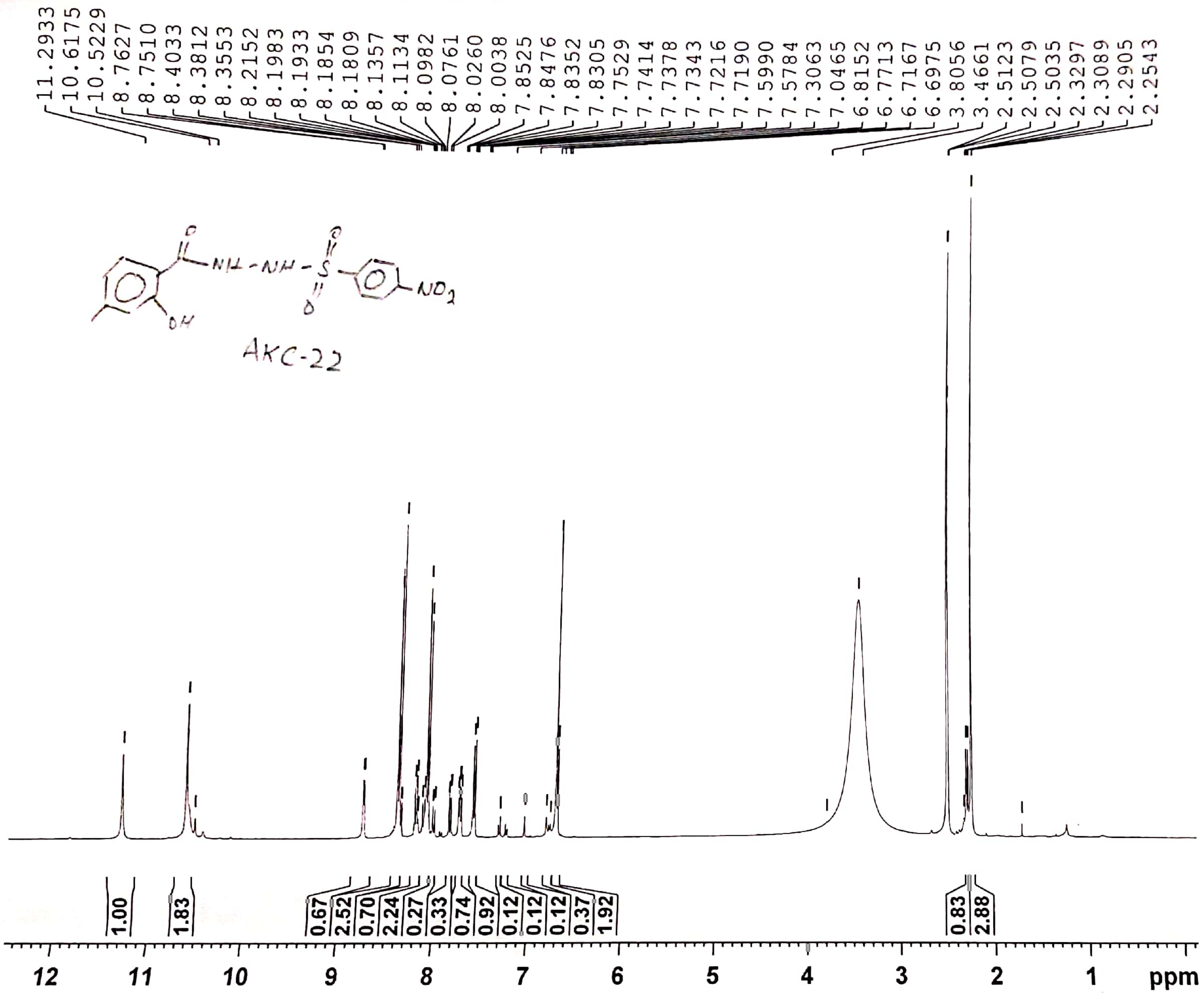

BRUKER

Current Data Parameters	
NAME	Dr. Amara
EXPNO	62
PROCNO	1

F2 - Acquisition Parameters	
Date_	20190621
Time	18.51 h
INSTRUM	spect
PROBHD	Z116098_0621 (
PULPROG	zg30
TD	65536
SOLVENT	DMSO
NS	16
DS	2
SWH	8012.820 Hz
FIDRES	0.244532 Hz
AQ	4.0894465 sec
RG	78.01
DW	62.400 usec
DE	6.50 usec
TE	298.0 K
D1	1.00000000 sec
TD0	1
SFO1	400.1324708 MHz
NUC1	1H
P1	10.00 usec
PLW1	16.68099976 W

F2 - Processing parameters		
SI		65536
SF		400.1300000 MHz
WDW		EM
SSB	0	
LB		0.30 Hz
GB	0	
PC		1.00

Scanned with CamScanner

AKC-21/Adil Khushal/Dr Amara/CDCl3

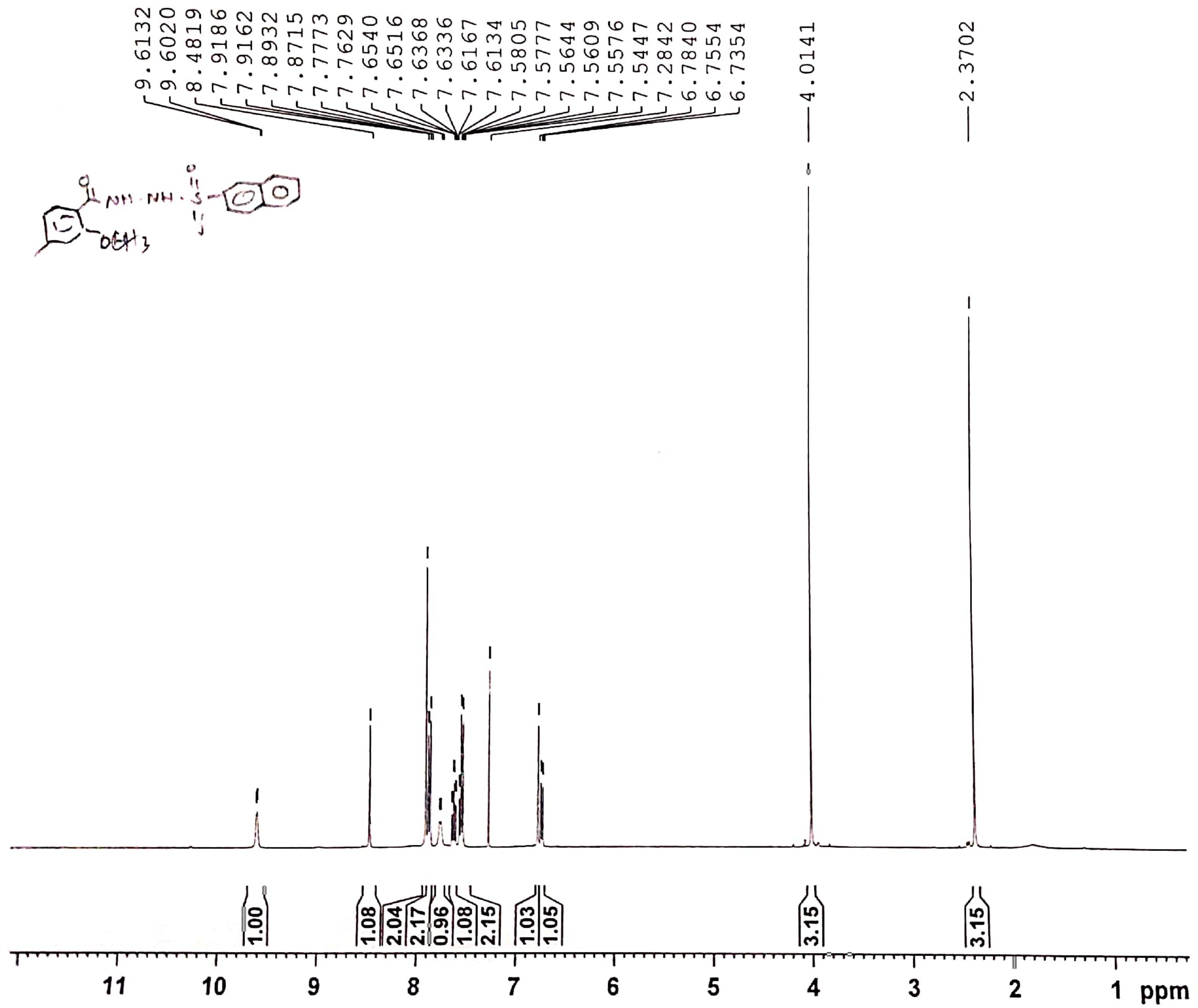

BRUKER

Current Data Parameters

NAME	Dr. Amara
EXPNO	60
PROCNO	1

F2 - Acquisition Parameters

Date_	20190615
Time	11.29 h
INSTRUM	spect
PROBHD	Z116098_0621 (
PULPROG	zg30
TD	65536
SOLVENT	CDCl3
NS	16
DS	2
SWH	8012.820 Hz
FIDRES	0.244532 Hz
AQ	4.0894465 sec
RG	98.53
DW	62.400 usec
DE	6.50 usec
TE	298.0 K
D1	1.00000000 sec
TD0	1
SFO1	400.1324708 MHz
NUC1	1H
P1	10.00 usec
PLW1	16.68099976 W

F2 - Processing parameters

SI		65536
SF		400.1300000 MHz
WDW		EM
SSB	0	
LB		0.30 Hz
GB	0	
PC		1.00

Scanned with CamScanner

AKC-18/Adil Khushal/Dr Amara/CDCl3

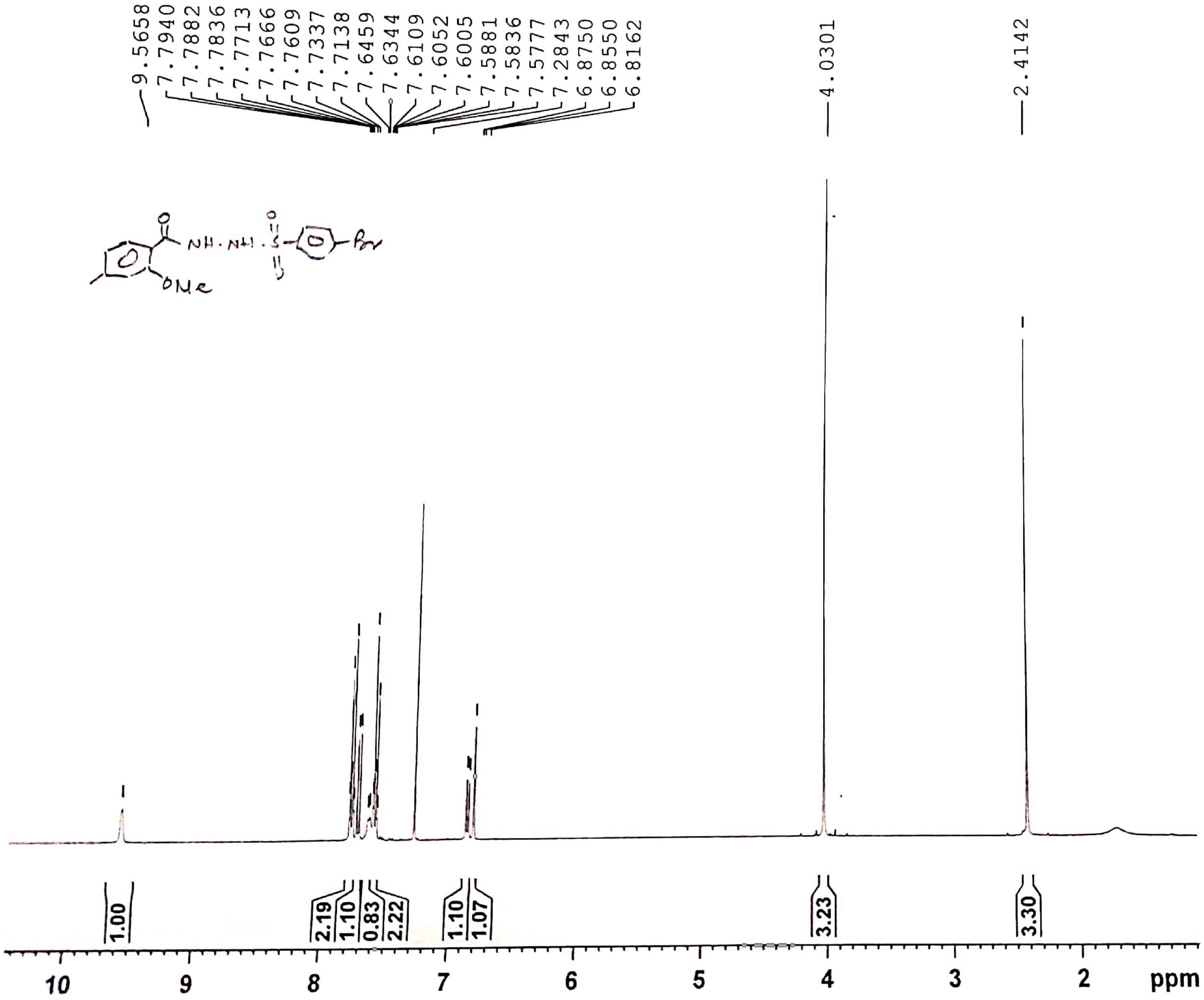

Current Data Parameters

NAME	Dr. Amara
EXPNO	59
PROCNO	1

F2 - Acquisition Parameters

Date_	20190615
Time	11.40 h
INSTRUM	spect
PROBHD	Z116098_0621 (
PULPROG	zg30
TD	65536
SOLVENT	CDCl3
NS	16
DS	2
SWH	8012.820 Hz
FIDRES	0.244532 Hz
AQ	4.0894465 sec
RG	127.9
DW	62.400 usec
DE	6.50 usec
TE	298.0 K
D1	1.00000000 sec
TD0	1
SFO1	400.1324708 MHz
NUC1	1H
P1	10.00 usec
PLW1	16.68099976 W

F2 - Processing parameters

SI		65536
SF		400.1300000 MHz
WDW		EM
SSB	0	
LB		0.30 Hz
GB	0	
PC		1.00

Scanned with CamScanner

AKC-17/Adil Khushal/Dr Amara/DMSO

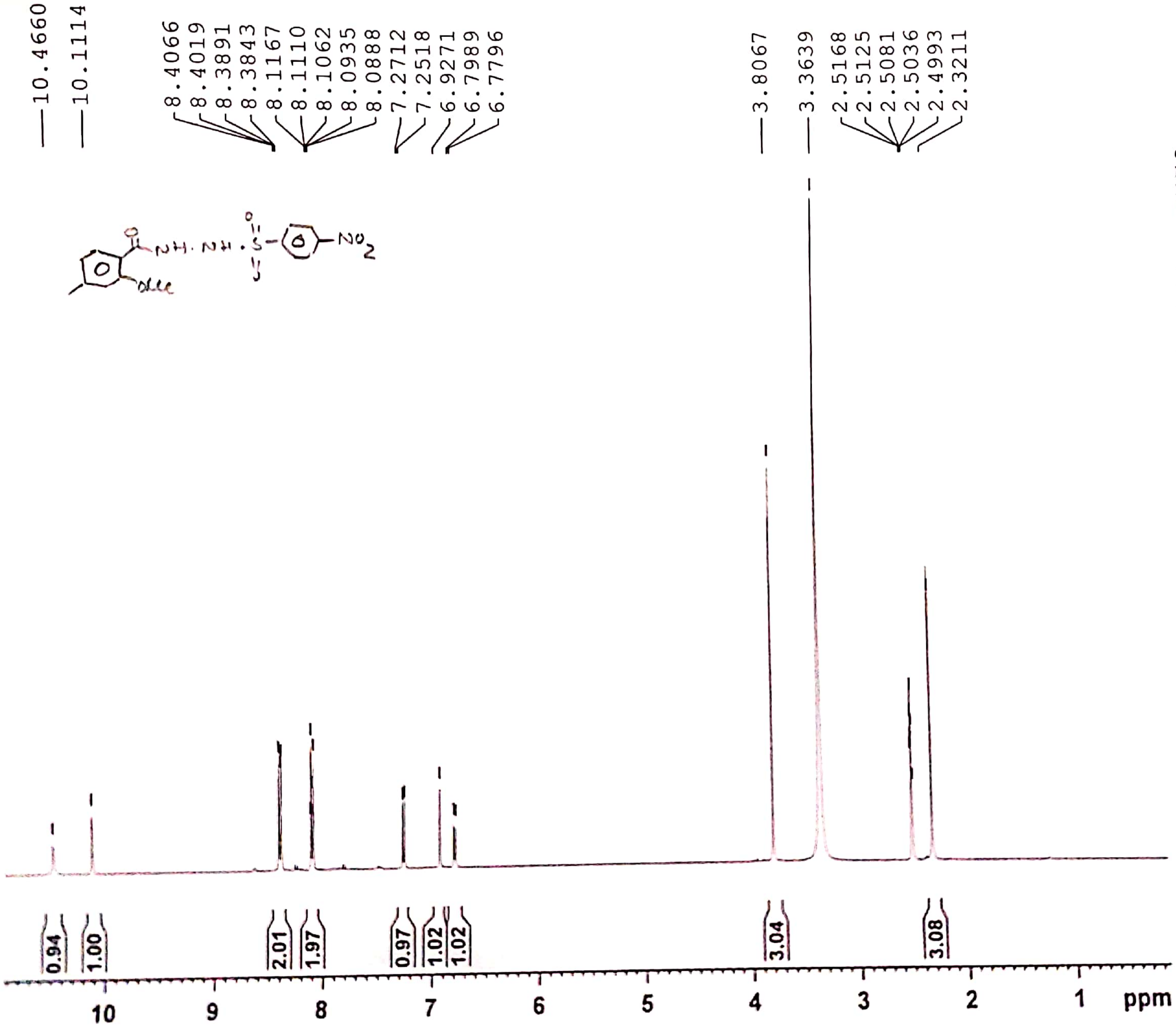

Current Data Parameters

NAME	Dr. Amara
EXPNO	53
PROCNO	1

F2 - Acquisition Parameters

Date_	20190615
Time	9.58 h
INSTRUM	spect
PROBHD	Z116098_0621 (
PULPROG	zg30
TD	65536
SOLVENT	DMSO
NS	16
DS	2
SWH	8012.820 Hz
FIDRES	0.244532 Hz
AQ	4.0894465 sec
RG	78.01
DW	62.400 usec
DE	6.50 usec
TE	298.0 K
D1	1.00000000 sec
TD0	1
SFO1	400.1324708 MHz
NUC1	1H
P1	10.00 usec
PLW1	16.68099976 W

F2 - Processing parameters

SI	65536
SF	400.1300000 MHz
WDW	EM
SSB	0
LB	0.30 Hz
GB	0
PC	1.00

Scanned with CamScanner

AKC-8/Adil Khushal/Dr Amara/CDCl3

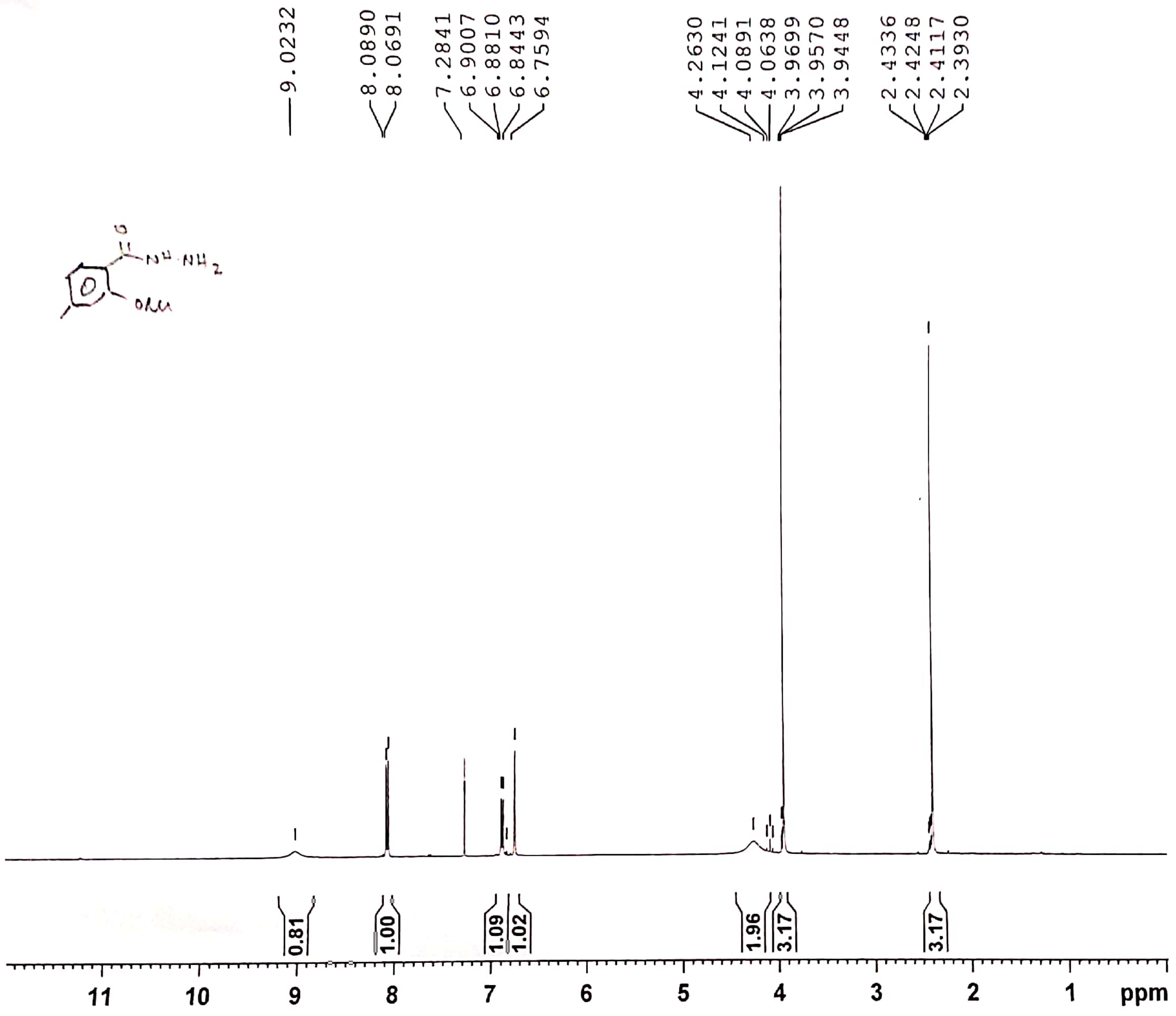

Current Data Parameters

NAME	Dr. Amara	
EXPNO	58	
PROCNO	1	

F2 - Acquisition Parameters

Date_	20190615	
Time	10.22	h
INSTRUM	spect	
PROBHD	Z116098_0621 (	
PULPROG	zg30	
TD	65536	
SOLVENT	CDCl3	
NS	16	
DS	2	
SWH	8012.820	Hz
FIDRES	0.244532	Hz
AQ	4.0894465	sec
RG	88.48	
DW	62.400	usec
DE	6.50	usec
TE	298.0	K
D1	1.00000000	sec
TD0	1	
SFO1	400.1324708	MHz
NUC1	1H	
P1	10.00	usec
PLW1	16.68099976	W

F2 - Processing parameters

SI	65536	
SF	400.1300000	MHz
WDW	EM	
SSB	0	
LB	0.30	Hz
GB	0	
PC	1.00	

Scanned with CamScanner

AMC-2/Abida Muneer/Dr. Amara/CDCl3

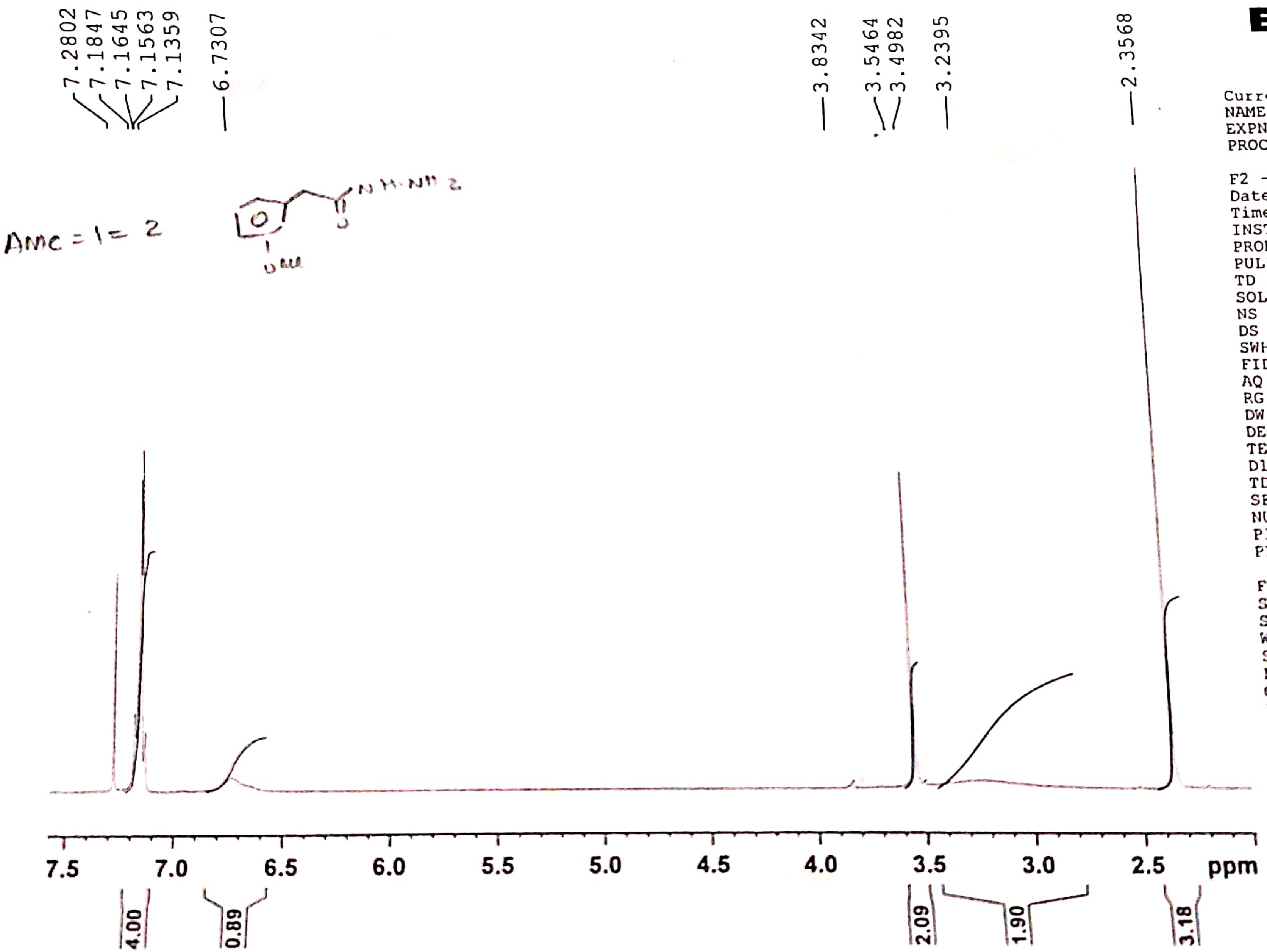

BRUKER

Current Data Parameters
NAME Dr. Amara
EXPNO 12
PROCNO 1

F2 - Acquisition Parameters
Date_ 20181015
Time 15.17 h
INSTRUM spect
PROBHD Z116098_0621 (
PULPROG zg30
TD 65536
SOLVENT CDCl3
NS 16
DS 2
SWH 8012.820 Hz
FIDRES 0.244532 Hz
AQ 4.0894465 sec
RG 112.2
DW 62.400 usec
DE 6.50 usec
TE 298.0 K
D1 1.00000000 sec
TD0 1
SFO1 400.1324708 MHz
NUC1 1H
P1 10.00 usec
PLW1 16.68099976 W

F2 - Processing parameters
SI 65536
SF 400.1300000 MHz
WDW EM
SSB 0
LB 0.30 Hz
GB 0
PC 1.00

Scanned with CamScanner

AMC-18/DMSO-d6

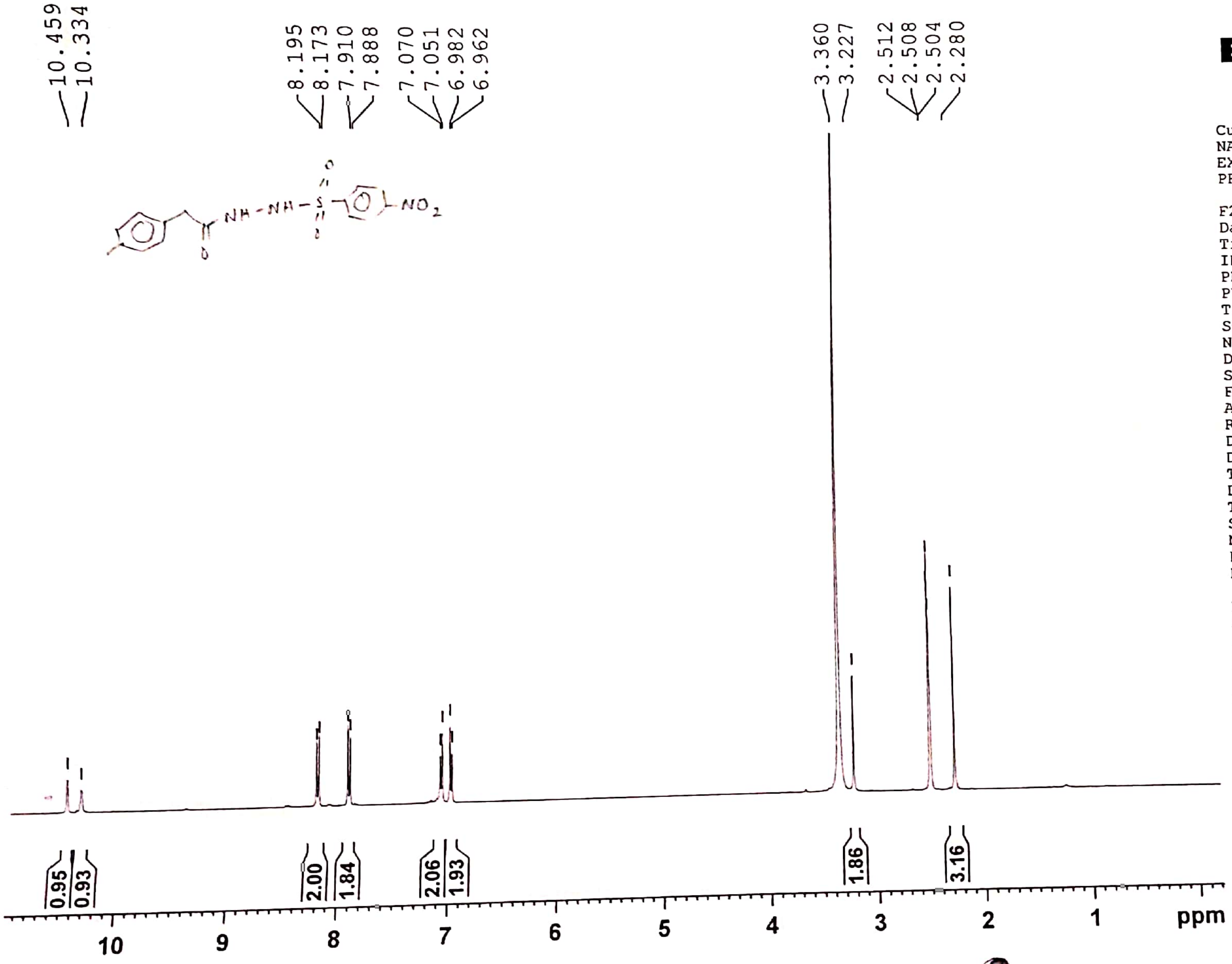

BRUKER

Current Data Parameters

NAME	Dr. Amara
EXPNO	52
PROCNO	1

F2 - Acquisition Parameters

Date_	20190516
Time	13.30 h
INSTRUM	spect
PROBHD	Z116098_0621 (
PULPROG	zg30
TD	65536
SOLVENT	DMSO
NS	16
DS	2
SWH	8012.820 Hz
FIDRES	0.244532 Hz
AQ	4.0894465 sec
RG	88.48
DW	62.400 usec
DE	6.50 usec
TE	298.0 K
D1	1.00000000 sec
TD0	1
SFO1	400.1324708 MHz
NUC1	1H
P1	10.00 usec
PLW1	16.68099976 W

F2 - Processing parameters

SI		65536
SF		400.1300000 MHz
WDW		EM
SSB	0	
LB		0.30 Hz
GB	0	
PC		1.00

Scanned with CamScanner

AMC-19/Abida Muneer/Dr Amara/CDCl3

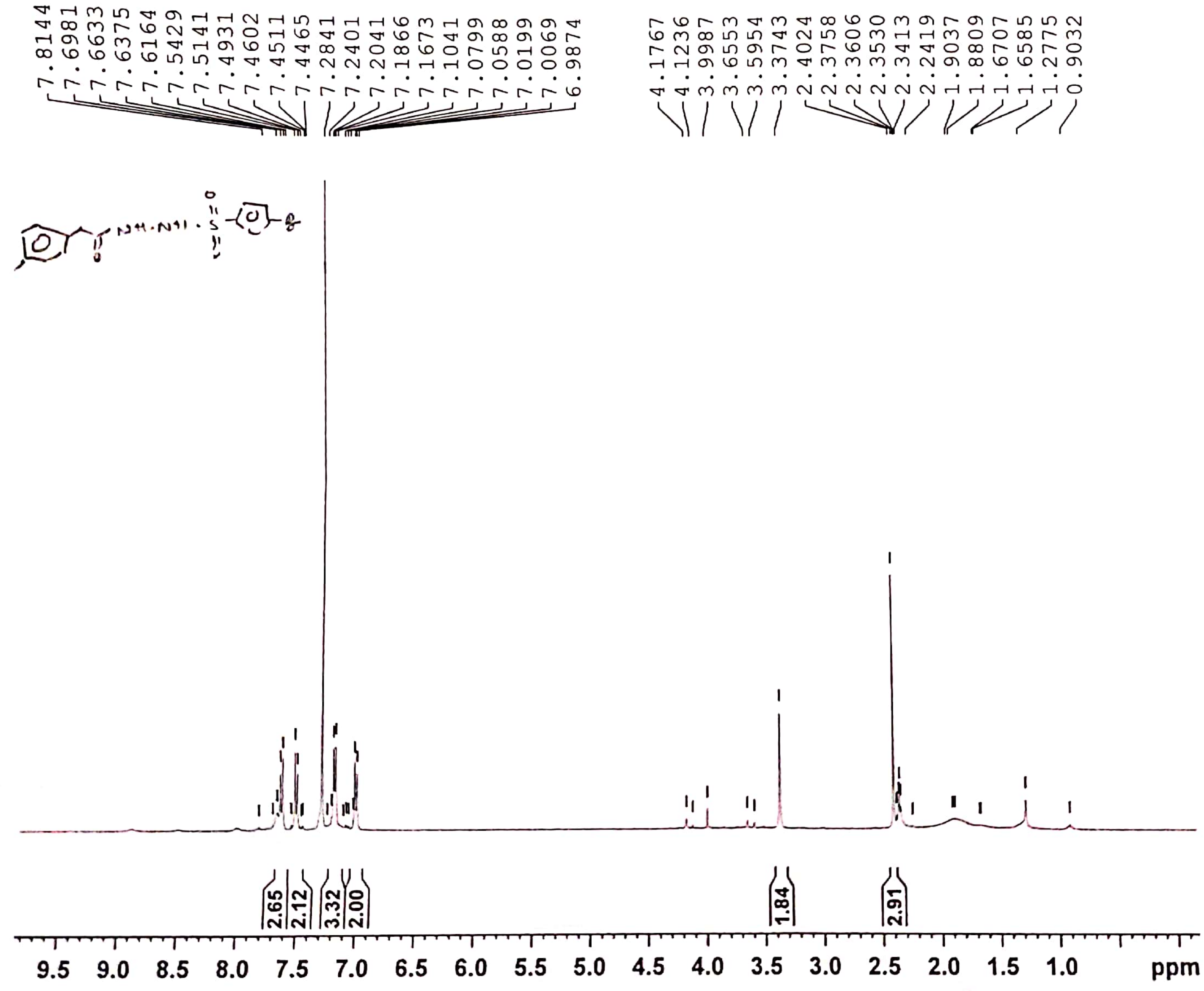

BRUKER

Current Data Parameters

NAME	Dr. Amara
EXPNO	55
PROCNO	1

F2 - Acquisition Parameters

Date_	20190615
Time	10.07 h
INSTRUM	spect
PROBHD	Z116098_0621 (
PULPROG	zg30
TD	65536
SOLVENT	CDCl3
NS	16
DS	2
SWH	8012.820 Hz
FIDRES	0.244532 Hz
AQ	4.0894465 sec
RG	143.62
DW	62.400 usec
DE	6.50 usec
TE	298.0 K
D1	1.00000000 sec
TD0	1
SFO1	400.1324708 MHz
NUC1	1H
P1	10.00 usec
PLW1	16.68099976 W

F2 - Processing parameters

SI		65536
SF		400.1300000 MHz
WDW		EM
SSB	0	
LB		0.30 Hz
GB	0	
PC		1.00

Scanned with CamScanner

AMC-22/Abida Muneer/Dr Amara/DMSO

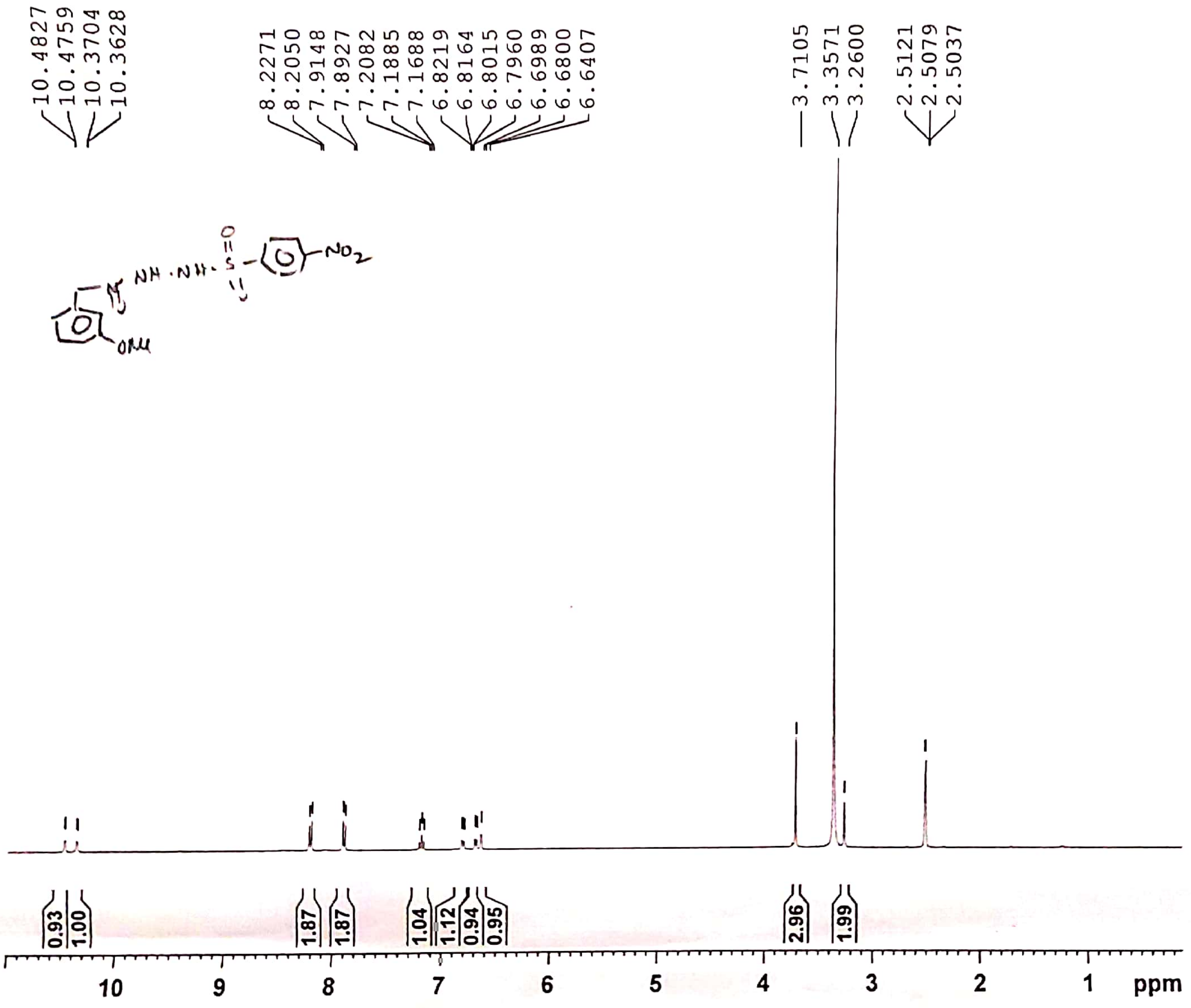

BRUKER

Current Data Parameters

NAME	Dr. Amara
EXPNO	54
PROCNO	1

F2 - Acquisition Parameters

Date_	20190615
Time	10.03 h
INSTRUM	spect
PROBHD	Z116098_0621 (
PULPROG	zg30
TD	65536
SOLVENT	DMSO
NS	16
DS	2
SWH	8012.820 Hz
FIDRES	0.244532 Hz
AQ	4.0894465 sec
RG	88.48
DW	62.400 usec
DE	6.50 usec
TE	298.0 K
D1	1.00000000 sec
TD0	1
SFO1	400.1324708 MHz
NUC1	1H
P1	10.00 usec
PLW1	16.68099976 W

F2 - Processing parameters

SI	65536
SF	400.1300000 MHz
WDW	EM
SSB	0
LB	0.30 Hz
GB	0
PC	1.00

Scanned with CamScanner

AMC-24/Abida Muneer/Dr. Amara/CDCl3

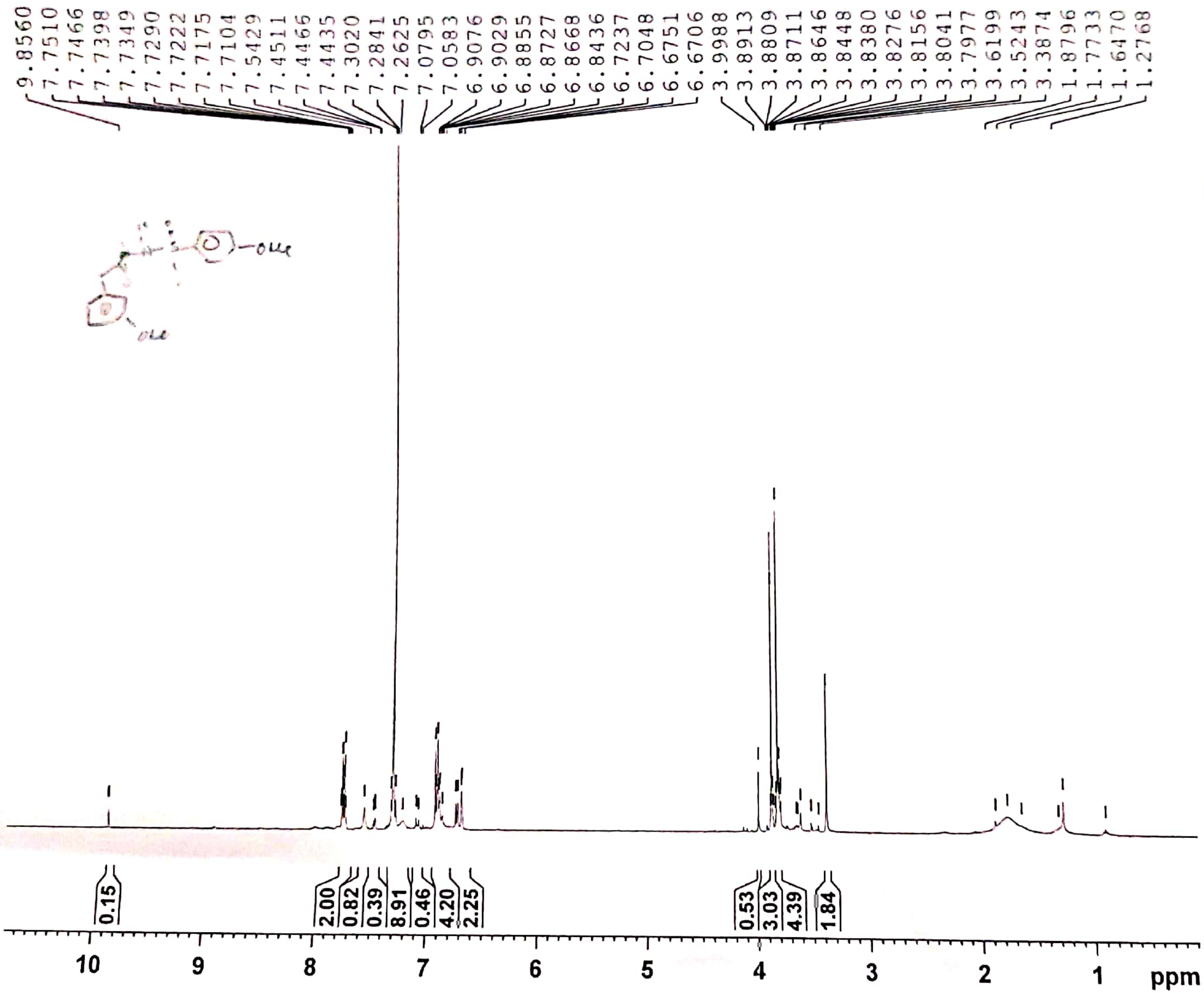

BRUKER

Current Data Parameters

NAME	Dr. Amara
EXPNO	69
PROCNO	1

F2 - Acquisition Parameters

Date_	20190621
Time	18.46 h
INSTRUM	spect
PROBHD	Z116098_0621 (
PULPROG	zg30
TD	65536
SOLVENT	CDCl3
NS	16
DS	2
SWH	8012.820 Hz
FIDRES	0.244532 Hz
AQ	4.0894465 sec
RG	143.62
DW	62.400 usec
DE	6.50 usec
TE	298.0 K
D1	1.00000000 sec
TD0	1
SFO1	400.1324708 MHz
NUC1	1H
P1	10.00 usec
PLW1	16.68099976 W

F2 - Processing parameters

SI	65536
SF	400.1300000 MHz
WDW	EM
SSB	0
LB	0.30 Hz
GB	0
PC	1.00

Scanned with CamScanner

AMC-23/Abida Muneer/Dr. Amara/CDCl3

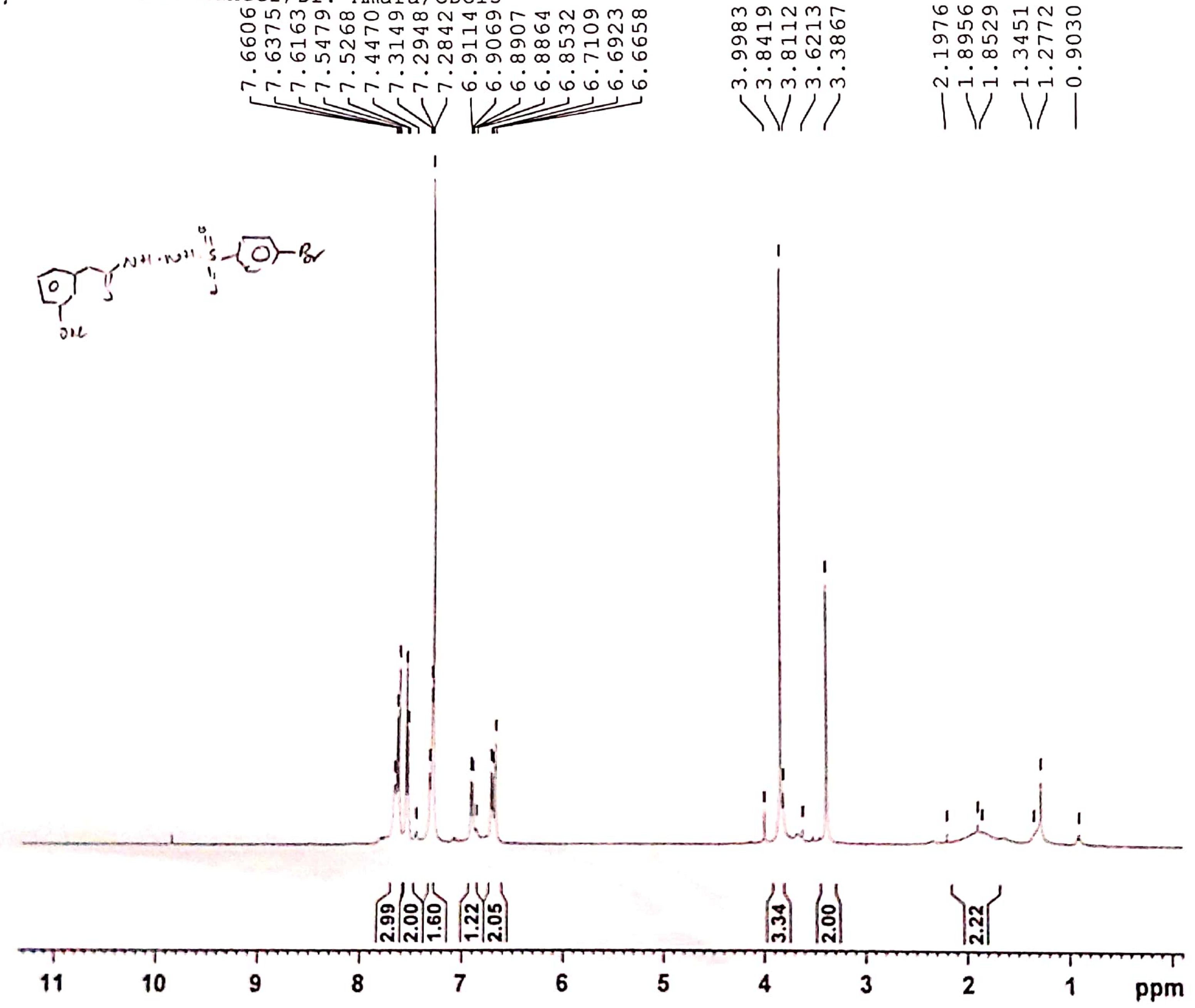

BRUKER

Current Data Parameters
NAME Dr. Amara
EXPNO 68
PROCNO 1

F2 - Acquisition Parameters

Date_	20190621
Time	18.41 h
INSTRUM	spect
PROBHD	Z116098_0621 (
PULPROG	zg30
TD	65536
SOLVENT	CDCl3
NS	16
DS	2
SWH	8012.820 Hz
FIDRES	0.244532 Hz
AQ	4.0894465 sec
RG	127.9
DW	62.400 usec
DE	6.50 usec
TE	298.0 K
D1	1.00000000 sec
TD0	1
SFO1	400.1324708 MHz
NUC1	1H
P1	10.00 usec
PLW1	16.68099976 W

F2 - Processing parameters

SI		65536
SF		400.1300000 MHz
WDW		EM
SSB	0	
LB		0.30 Hz
GB	0	
PC		1.00

Scanned with CamScanner

AMC-21/Abida Muneer/Dr Amara/CDCl3

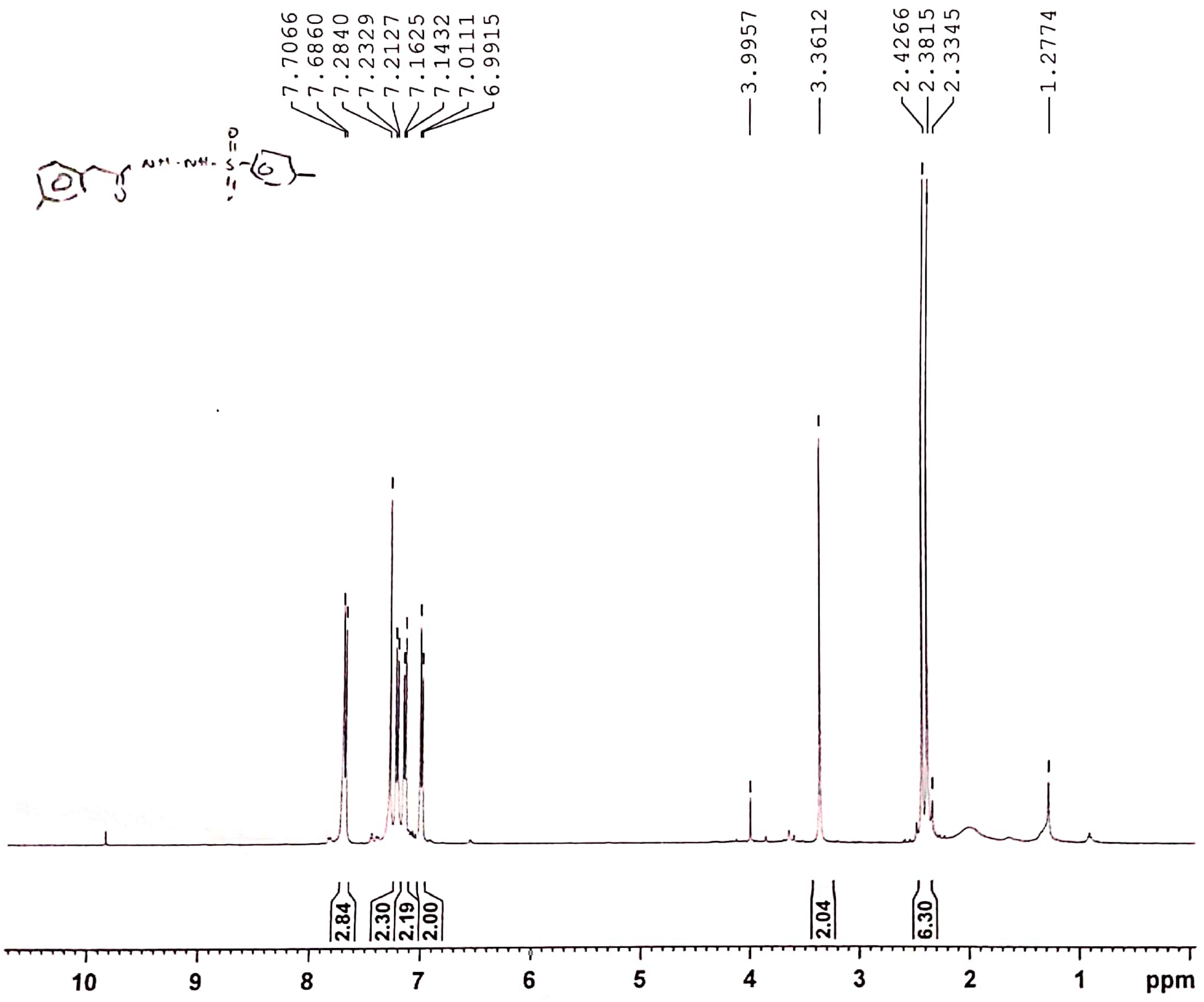

BRUKER

```
Current Data Parameters
NAME              Dr. Amara
EXPNO                    56
PROCNO                    1

F2 - Acquisition Parameters
Date_              20190615
Time                10.12 h
INSTRUM               spect
PROBHD     Z116098_0621 (
PULPROG                zg30
TD                    65536
SOLVENT               CDCl3
NS                       16
DS                        2
SWH              8012.820 Hz
FIDRES           0.244532 Hz
AQ              4.0894465 sec
RG                    112.2
DW                 62.400 usec
DE                   6.50 usec
TE                  298.0 K
D1             1.00000000 sec
TD0                       1
SFO1          400.1324708 MHz
NUC1                     1H
P1                  10.00 usec
PLW1          16.68099976 W

F2 - Processing parameters
SI                    65536
SF            400.1300000 MHz
WDW                      EM
SSB          0
LB                     0.30 Hz
GB           0
PC                     1.00
```

Scanned with CamScanner

AMC-20/Abida Muneer/Dr. Amara/CDCl3

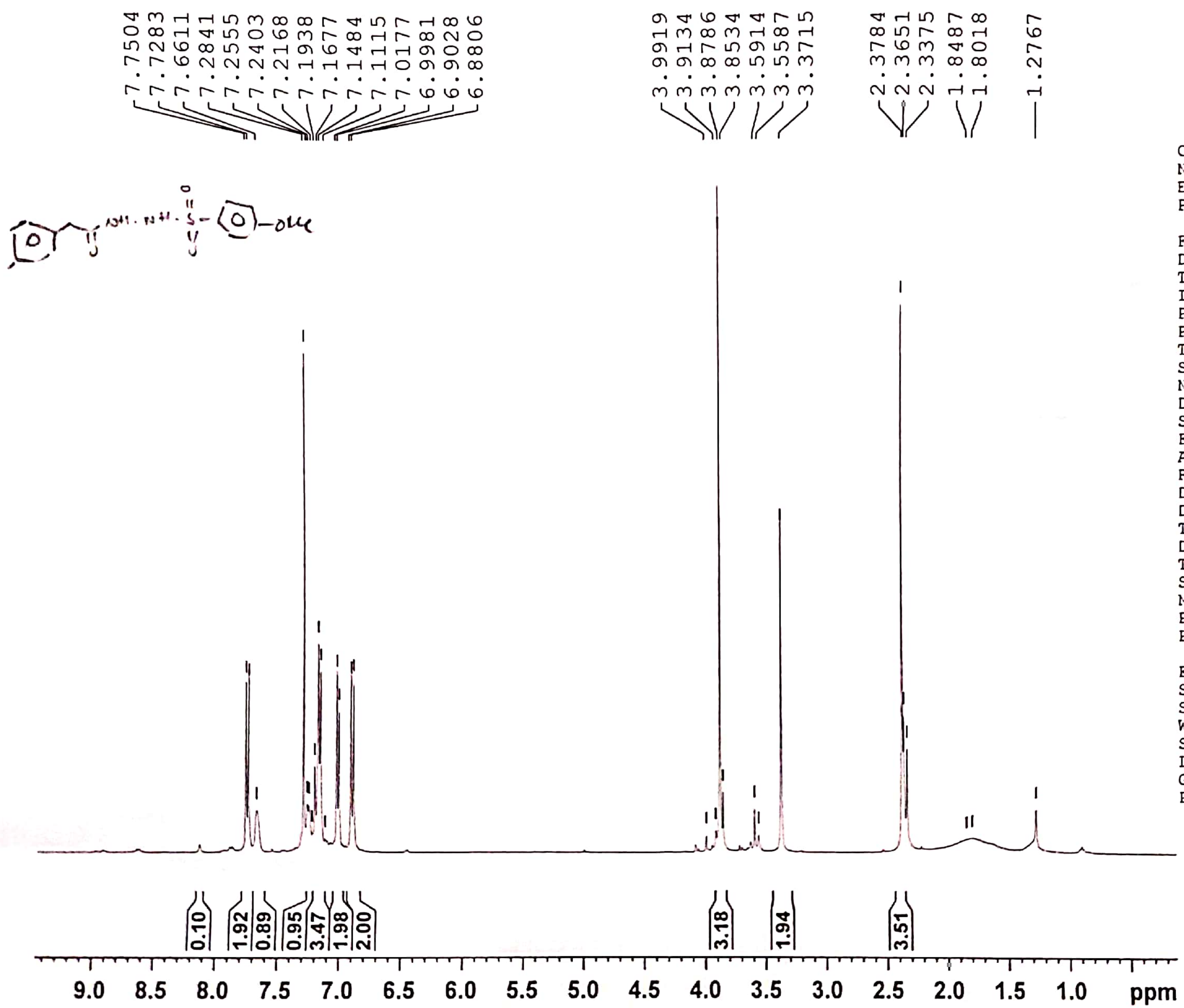

BRUKER

Current Data Parameters

NAME	Dr. Amara
EXPNO	67
PROCNO	1

F2 - Acquisition Parameters

Date_	20190621
Time_	18.37 h
INSTRUM	spect
PROBHD	Z116098_0621 (
PULPROG	zg30
TD	65536
SOLVENT	CDCl3
NS	16
DS	2
SWH	8012.820 Hz
FIDRES	0.244532 Hz
AQ	4.0894465 sec
RG	127.9
DW	62.400 usec
DE	6.50 usec
TE	298.0 K
D1	1.00000000 sec
TD0	1
SFO1	400.1324708 MHz
NUC1	1H
P1	10.00 usec
PLW1	16.68099976 W

F2 - Processing parameters

SI	65536
SF	400.1300000 MHz
WDW	EM
SSB	0
LB	0.30 Hz
GB	0
PC	1.00

Scanned with CamScanner

AMC-25/CDCl3

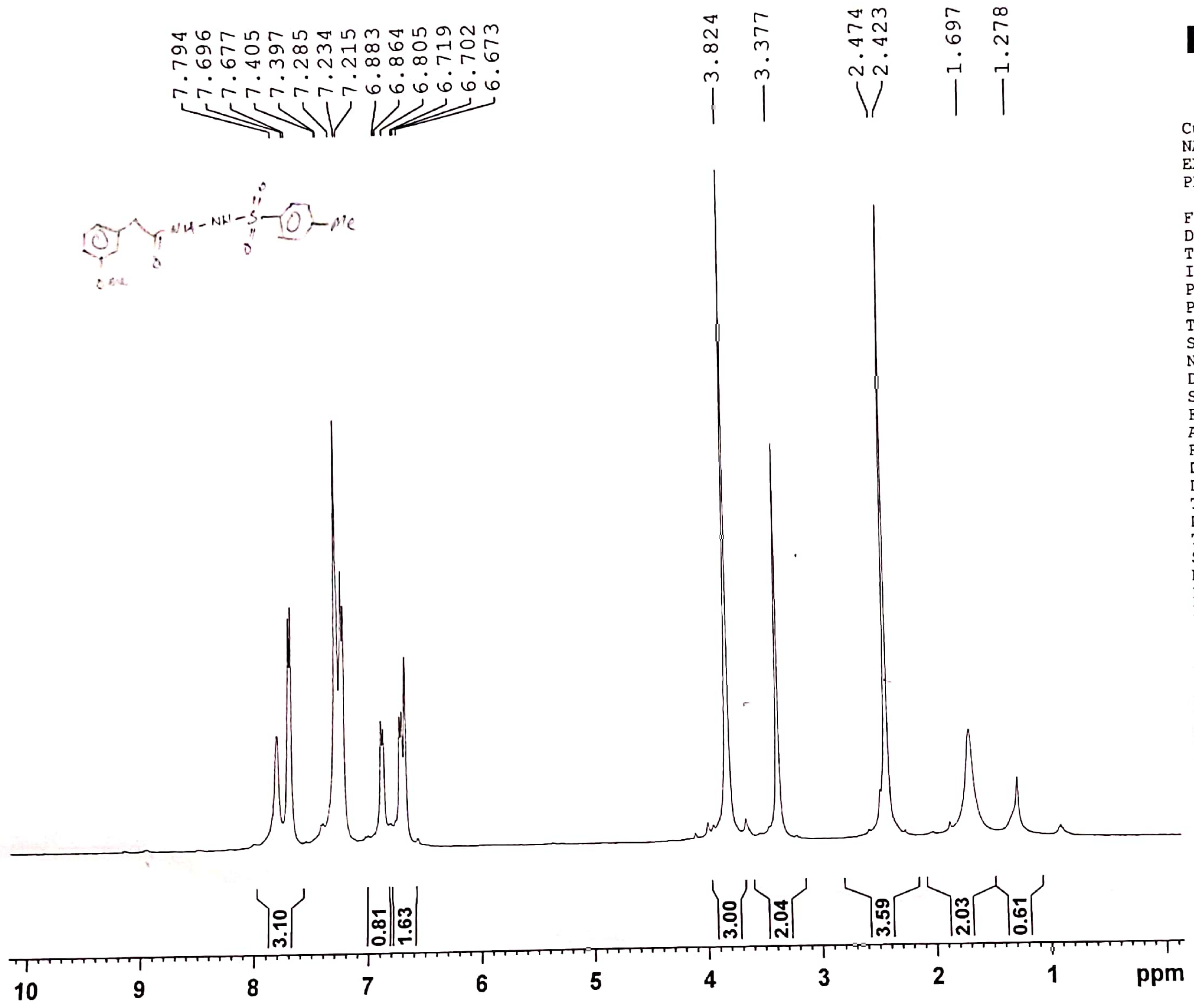

BRUKER

Current Data Parameters

NAME	Dr. Amara
EXPNO	50
PROCNO	1

F2 - Acquisition Parameters

Date_	20190516
Time	12.29 h
INSTRUM	spect
PROBHD	Z116098_0621 (
PULPROG	zg30
TD	65536
SOLVENT	CDCl3
NS	16
DS	2
SWH	8012.820 Hz
FIDRES	0.244532 Hz
AQ	4.0894465 sec
RG	98.53
DW	62.400 usec
DE	6.50 usec
TE	298.0 K
D1	1.00000000 sec
TD0	1
SFO1	400.1324708 MHz
NUC1	1H
P1	10.00 usec
PLW1	16.68099976 W

F2 - Processing parameters

SI	65536
SF	400.1300000 MHz
WDW	EM
SSB	0
LB	0.30 Hz
GB	0
PC	1.00

Scanned with CamScanner

AMC-27/Abida Muneer/Dr Amara/CDCl3

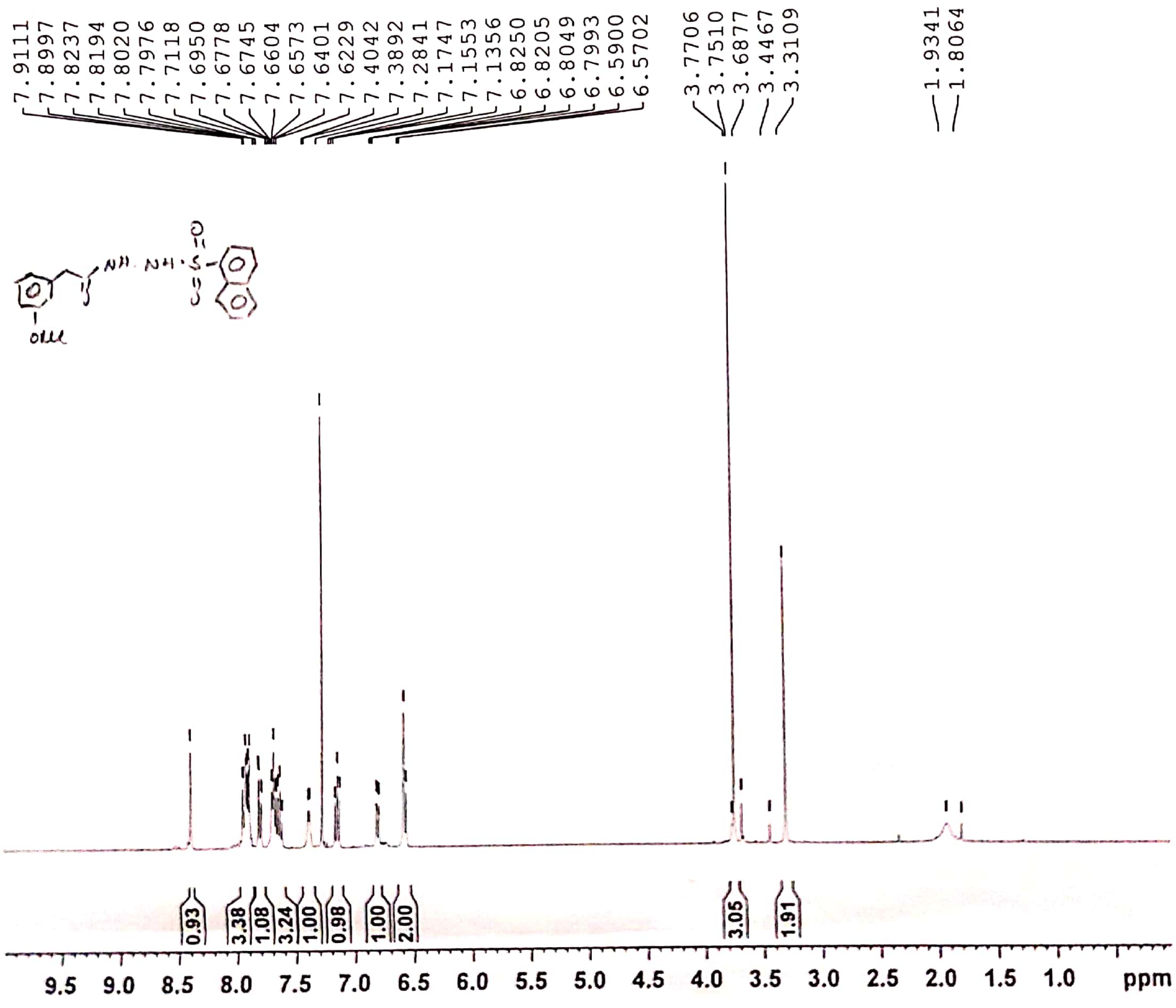

BRUKER

Current Data Parameters	
NAME	Dr. Amara
EXPNO	57
PROCNO	1
F2 - Acquisition Parameters	
Date_	20190615
Time	10.17 h
INSTRUM	spect
PROBHD	Z116098_0621 (
PULPROG	zg30
TD	65536
SOLVENT	CDCl3
NS	16
DS	2
SWH	8012.820 Hz
FIDRES	0.244532 Hz
AQ	4.0894465 sec
RG	127.9
DW	62.400 usec
DE	6.50 usec
TE	298.0 K
D1	1.00000000 sec
TD0	1
SFO1	400.1324708 MHz
NUC1	1H
P1	10.00 usec
PLW1	16.68099976 W
F2 - Processing parameters	
SI	65536
SF	400.1300000 MHz
WDW	EM
SSB	0
LB	0.30 Hz
GB	0
PC	1.00

Scanned with CamScanner

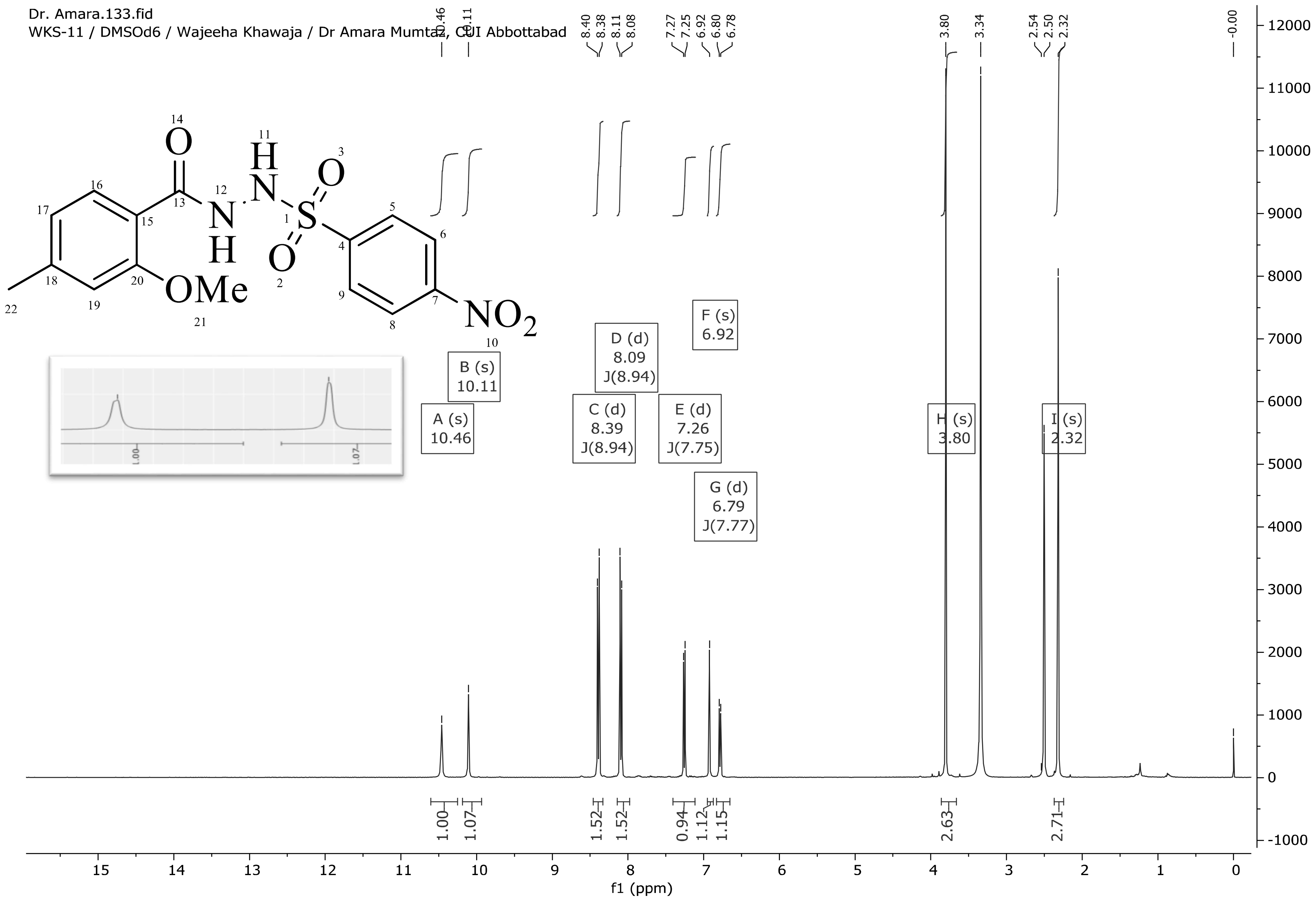

Dr. Amara.133.fid
WKS-11 / DMSOd6 / Wajeeha Khawaja / Dr Amara Mumtaz, CUI Abbottabad
A (s) 10.46
B (s) 10.11
C (d) 8.39 J(8.94)
D (d) 8.09 J(8.94)
E (d) 7.26 J(7.75)
F (s) 6.92
G (d) 6.79 J(7.77)
H (s) 3.80
I (s) 2.32
1.00
1.07
1.52
1.52
0.94
1.12
1.15
2.63
2.71
f1 (ppm)

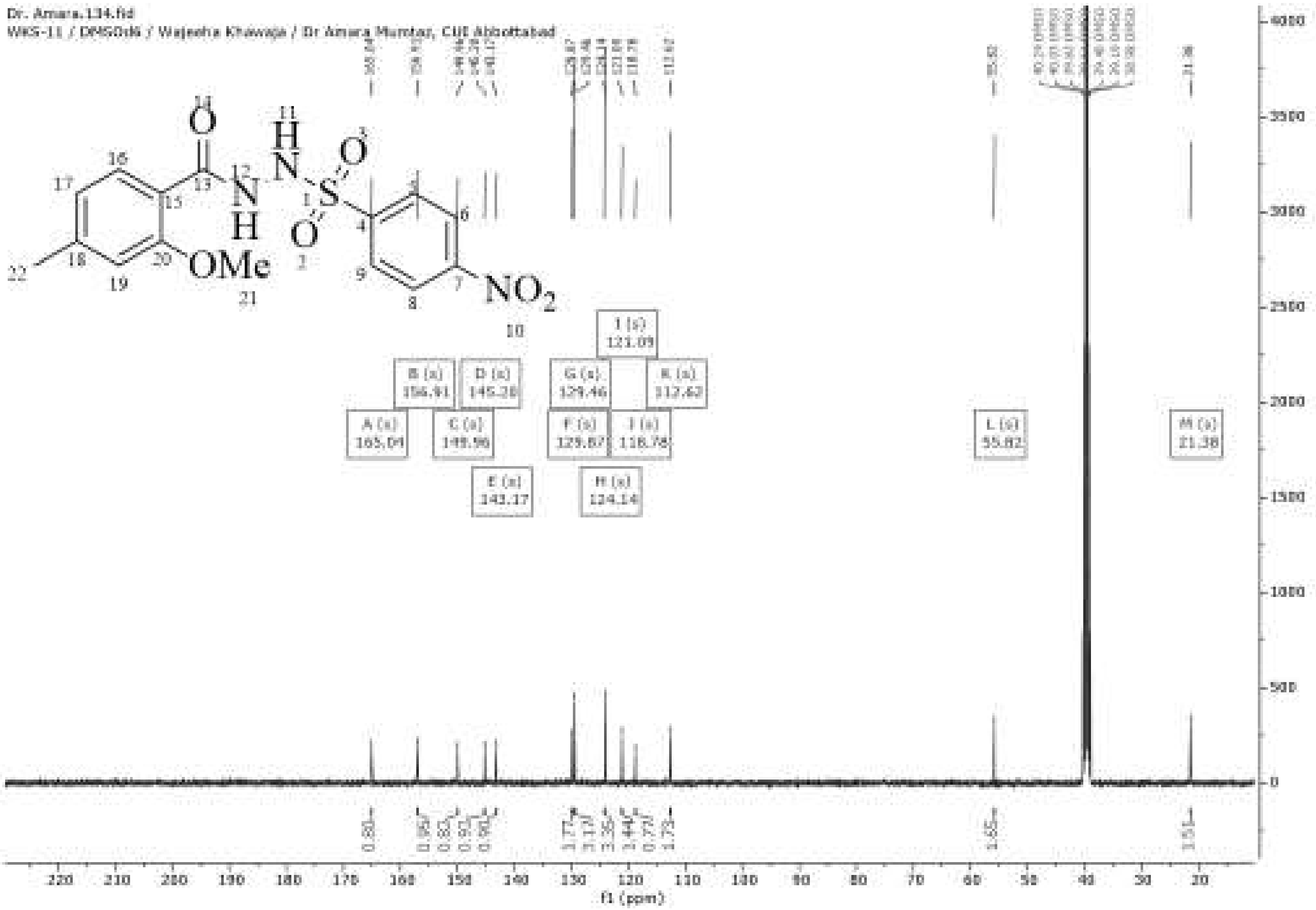

Dr. Amara.134.fid
WKS-11 / DMSOd6 / Wajeeha Khawaja / Dr Amara Mumtaz, CUI Abbottabad
OMe
NO2
A (s) 165.04
B (s) 156.91
C (s) 149.96
D (s) 145.28
E (s) 143.17
F (s) 129.87
G (s) 129.46
H (s) 124.14
I (s) 121.09
J (s) 118.78
K (s) 112.62
L (s) 55.82
M (s) 21.38
f1 (ppm)

Window Display Report

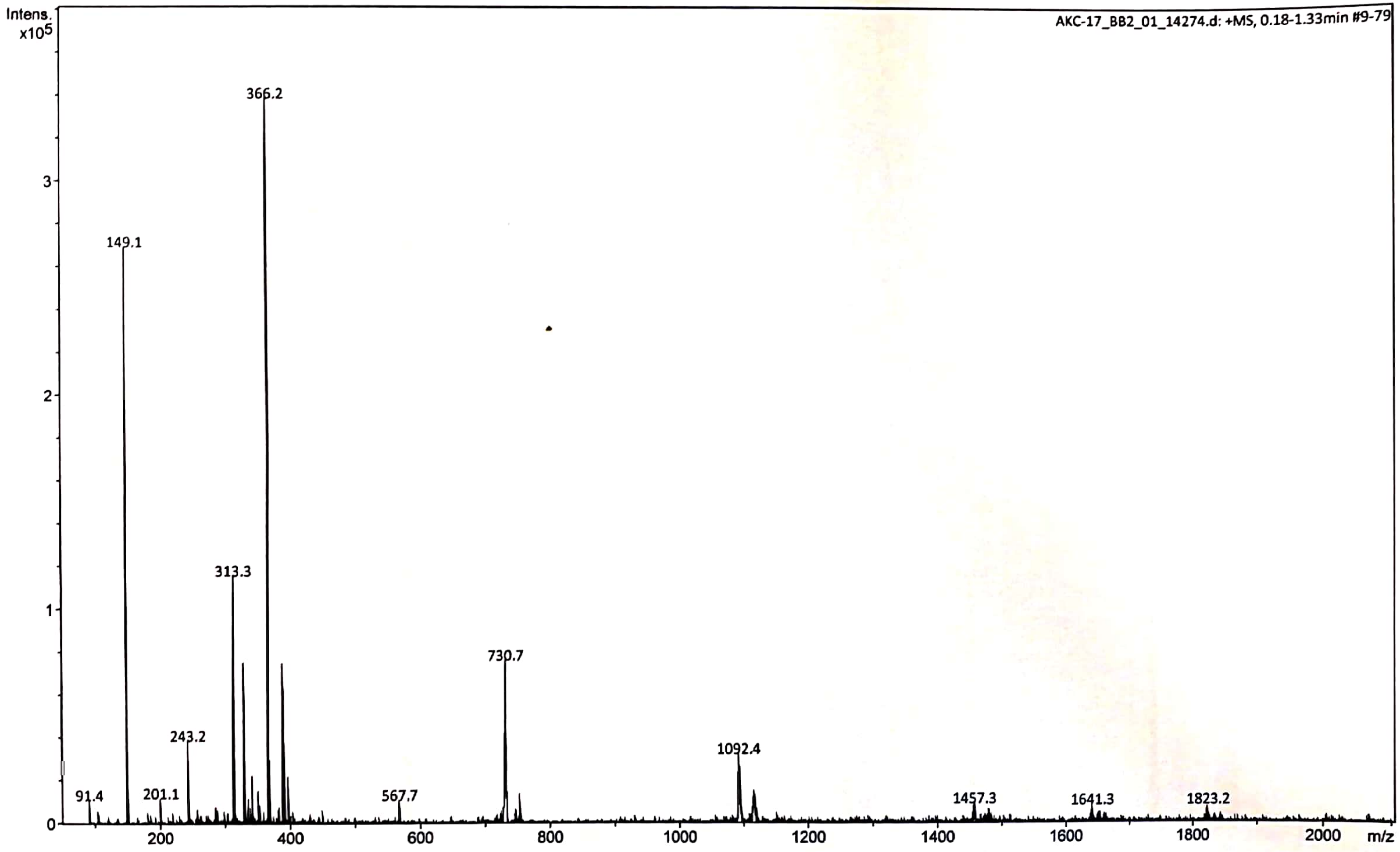

Scanned with CamScanner

Window Display Report

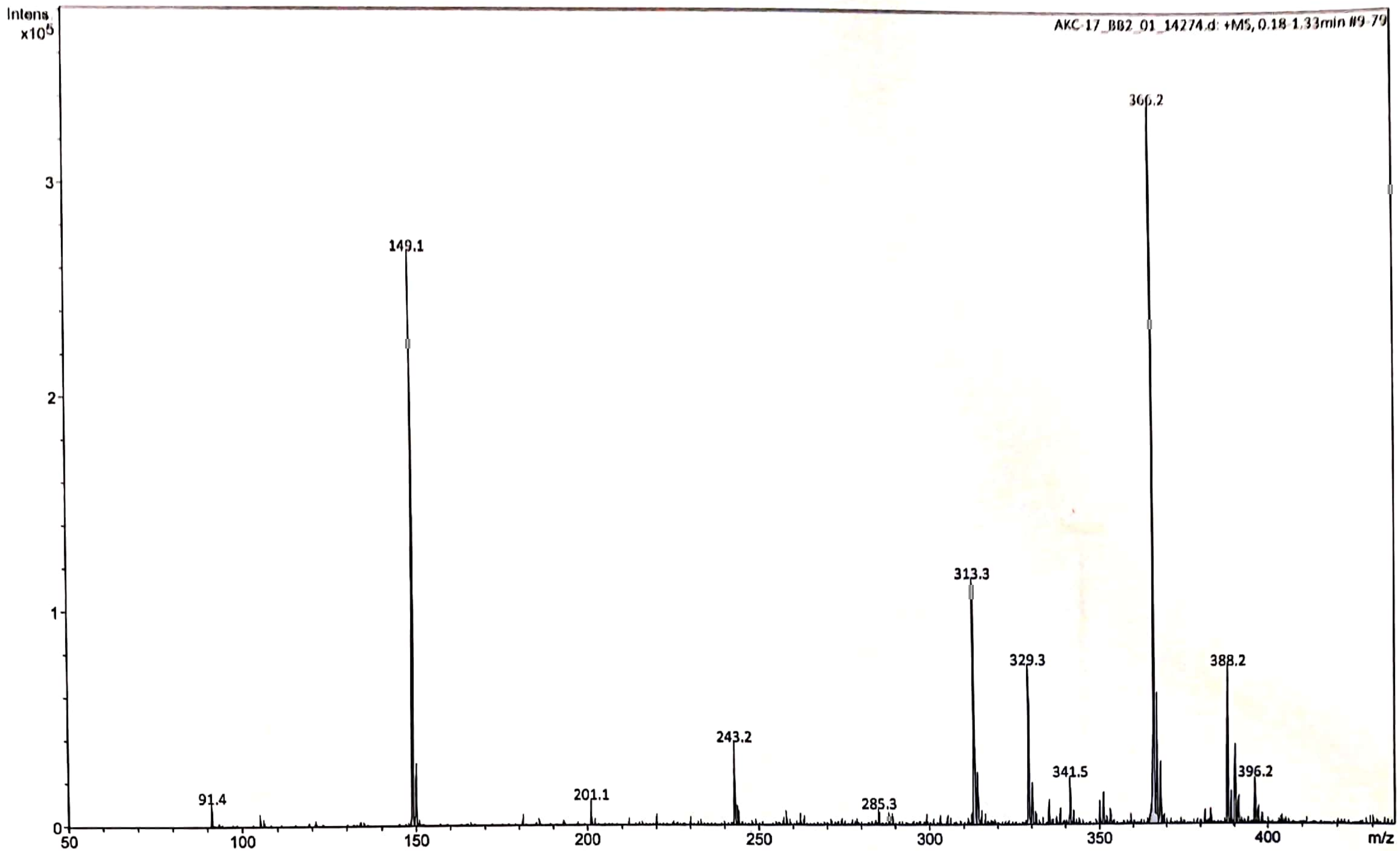

Scanned with CamScanner

Window Display Report

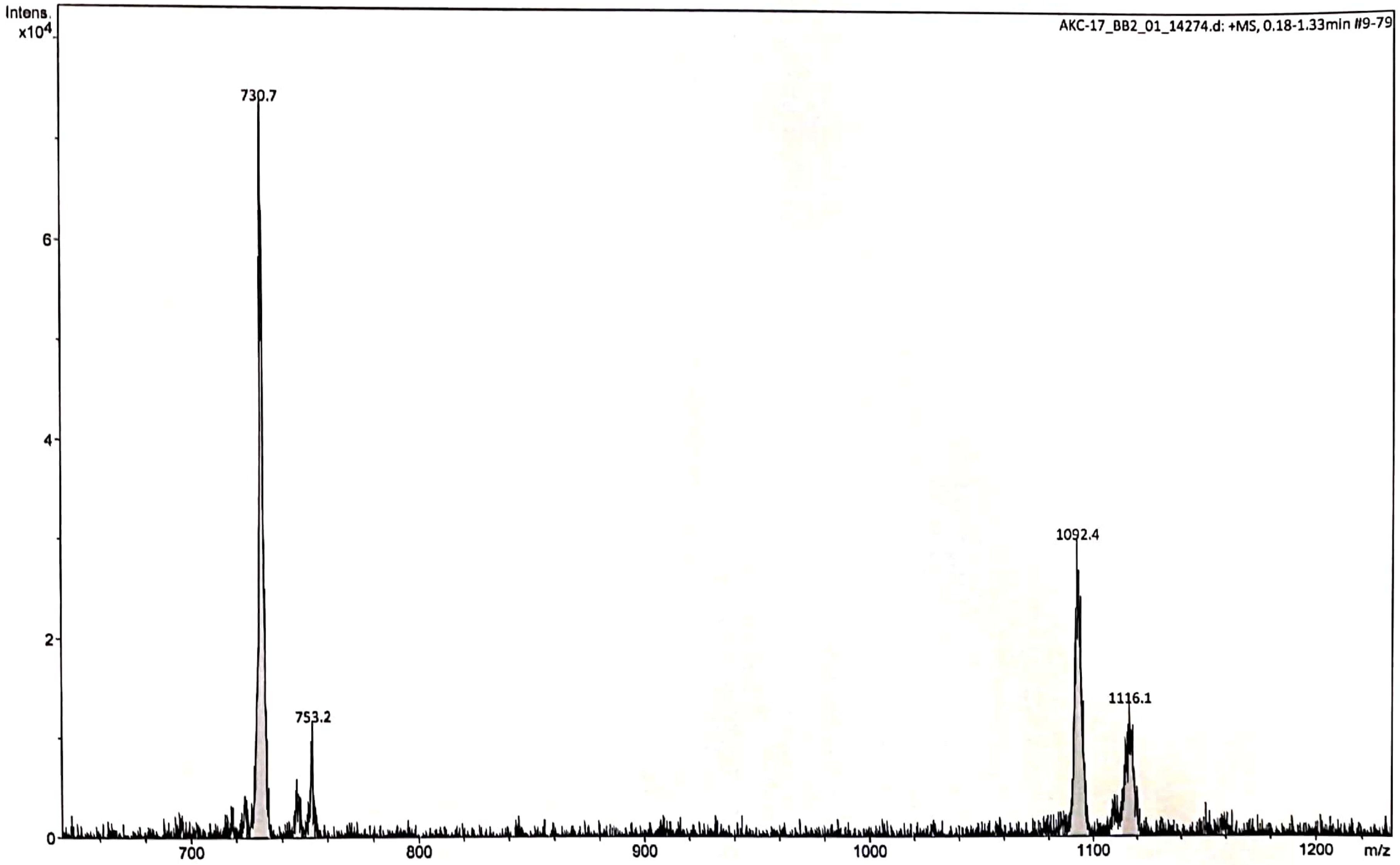

Scanned with CamScanner

Window Display Report

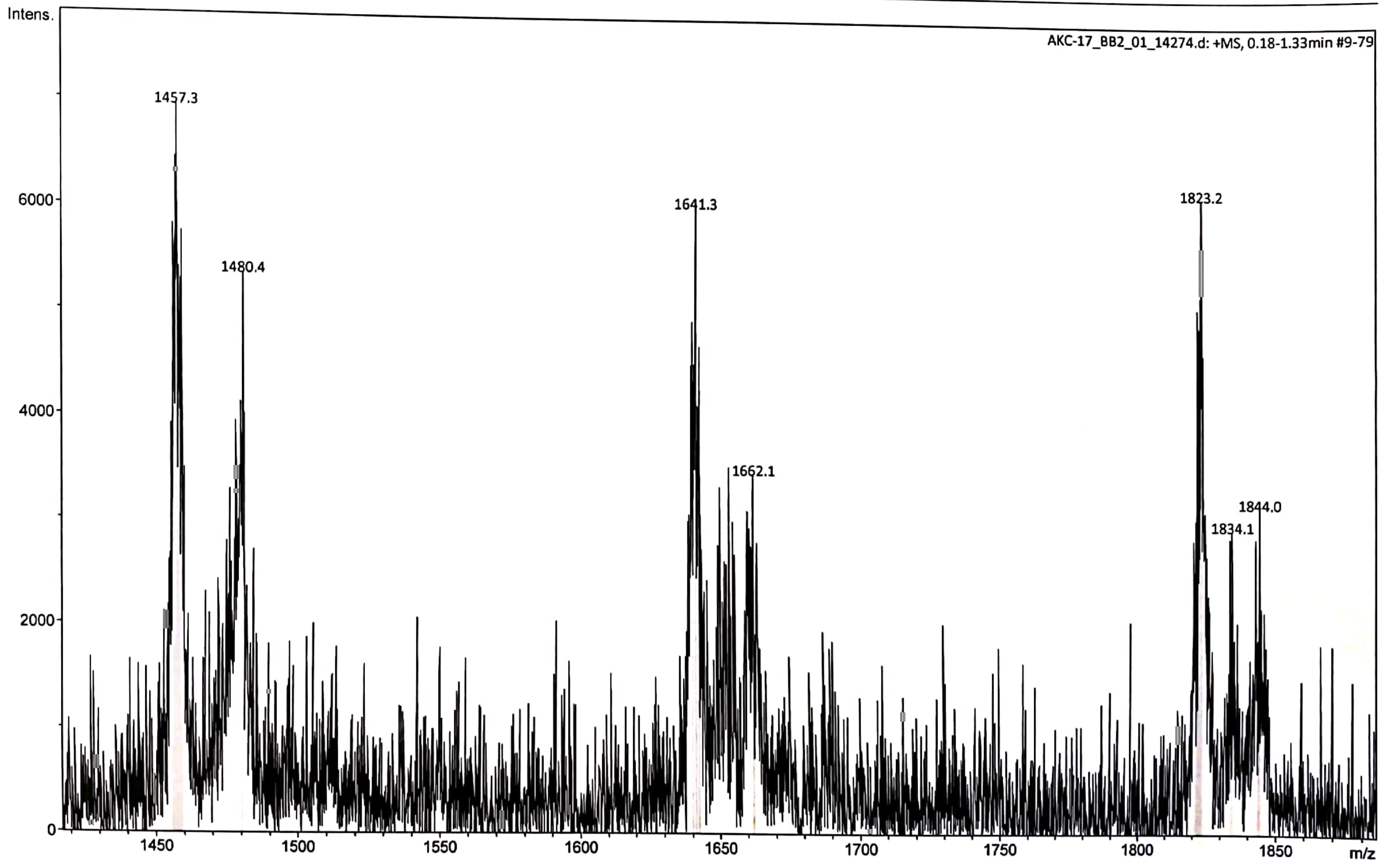

Scanned with CamScanner

Window Display Report

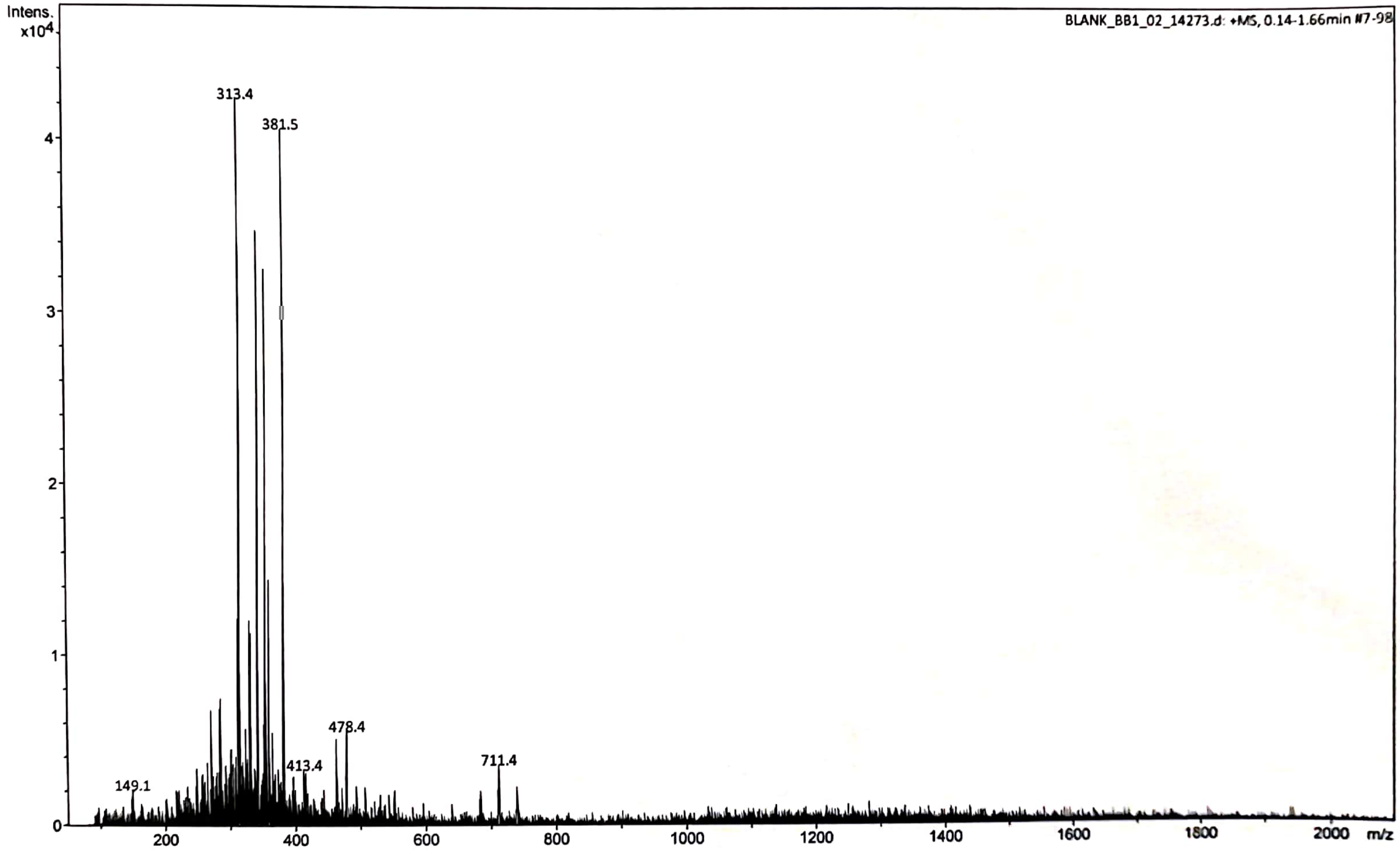

Scanned with CamScanner

Window Display Report

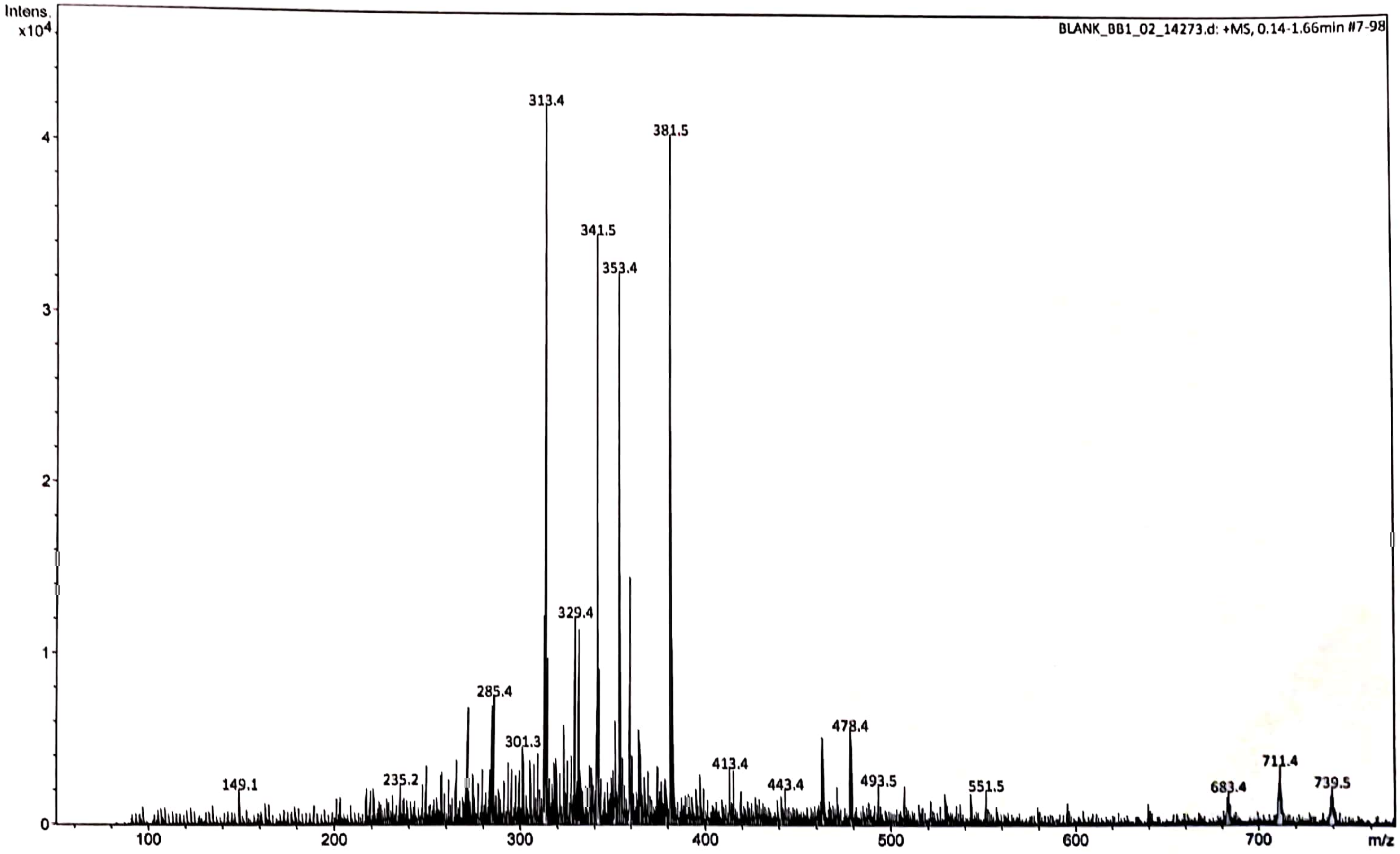

Scanned with CamScanner

Dr. Amara.184.fid — AKC-18/CDCl3/Adil/Dr. Amara Mumtaz/CUI-ATD

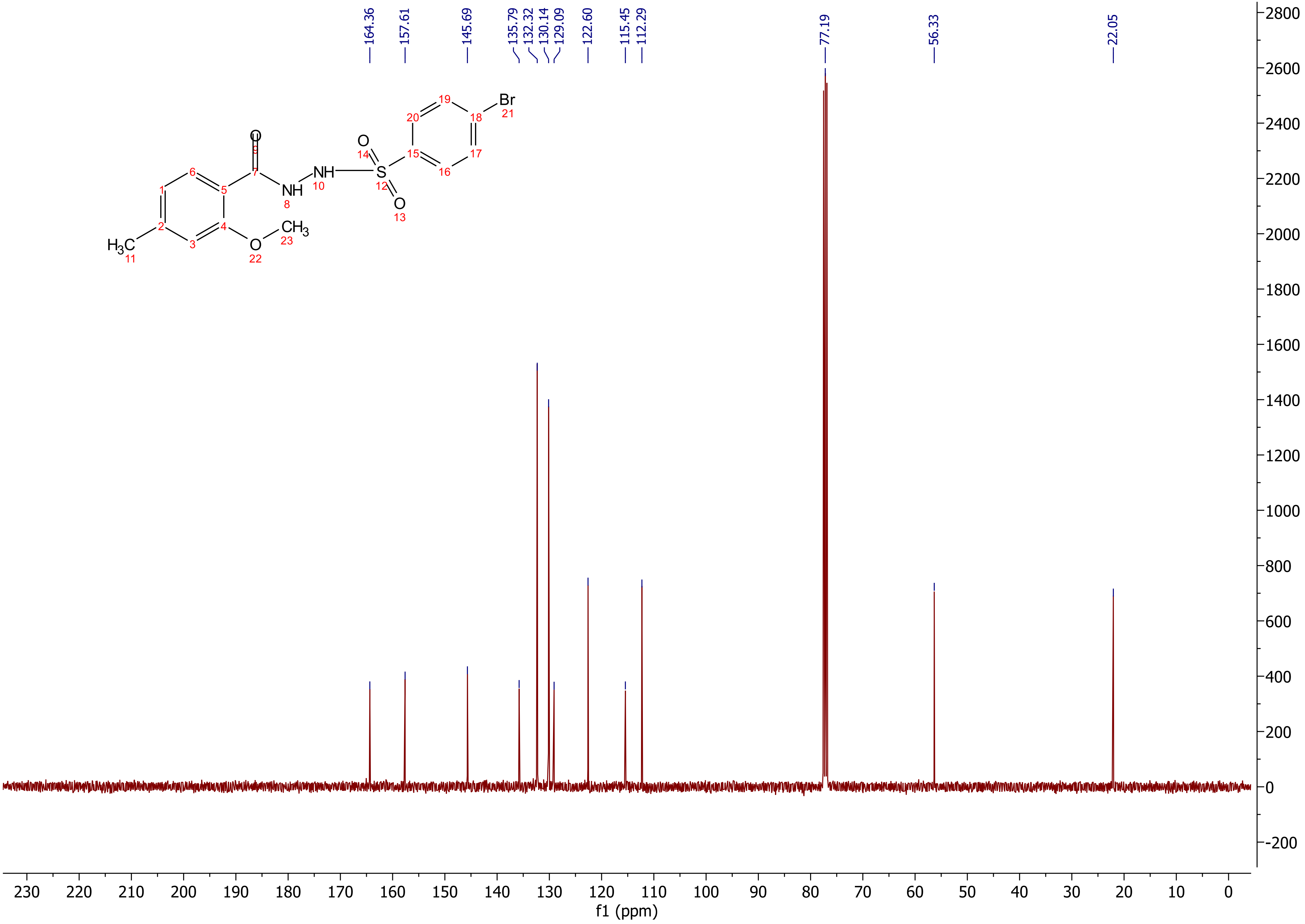

Window Display Report

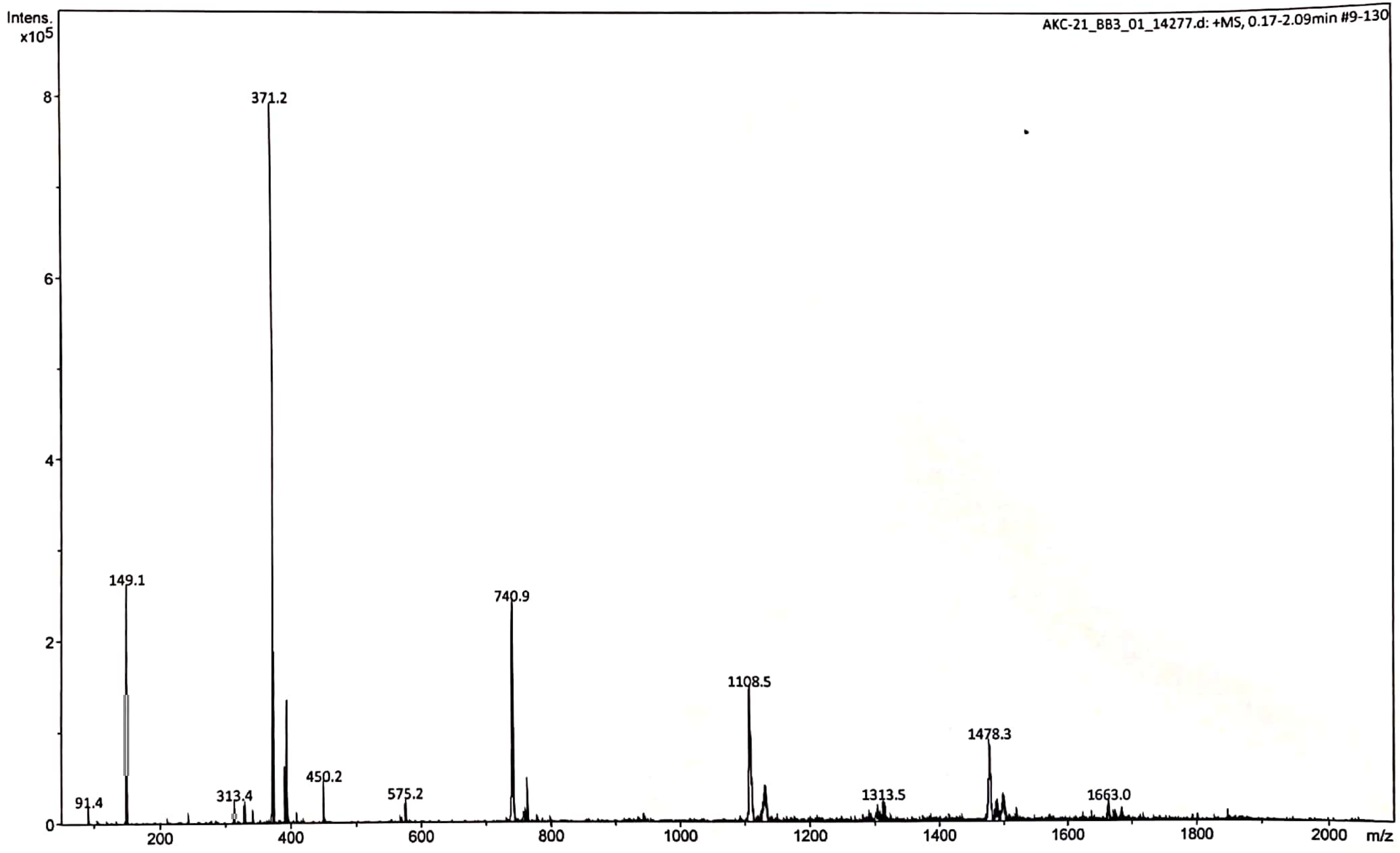

Scanned with CamScanner

Window Display Report

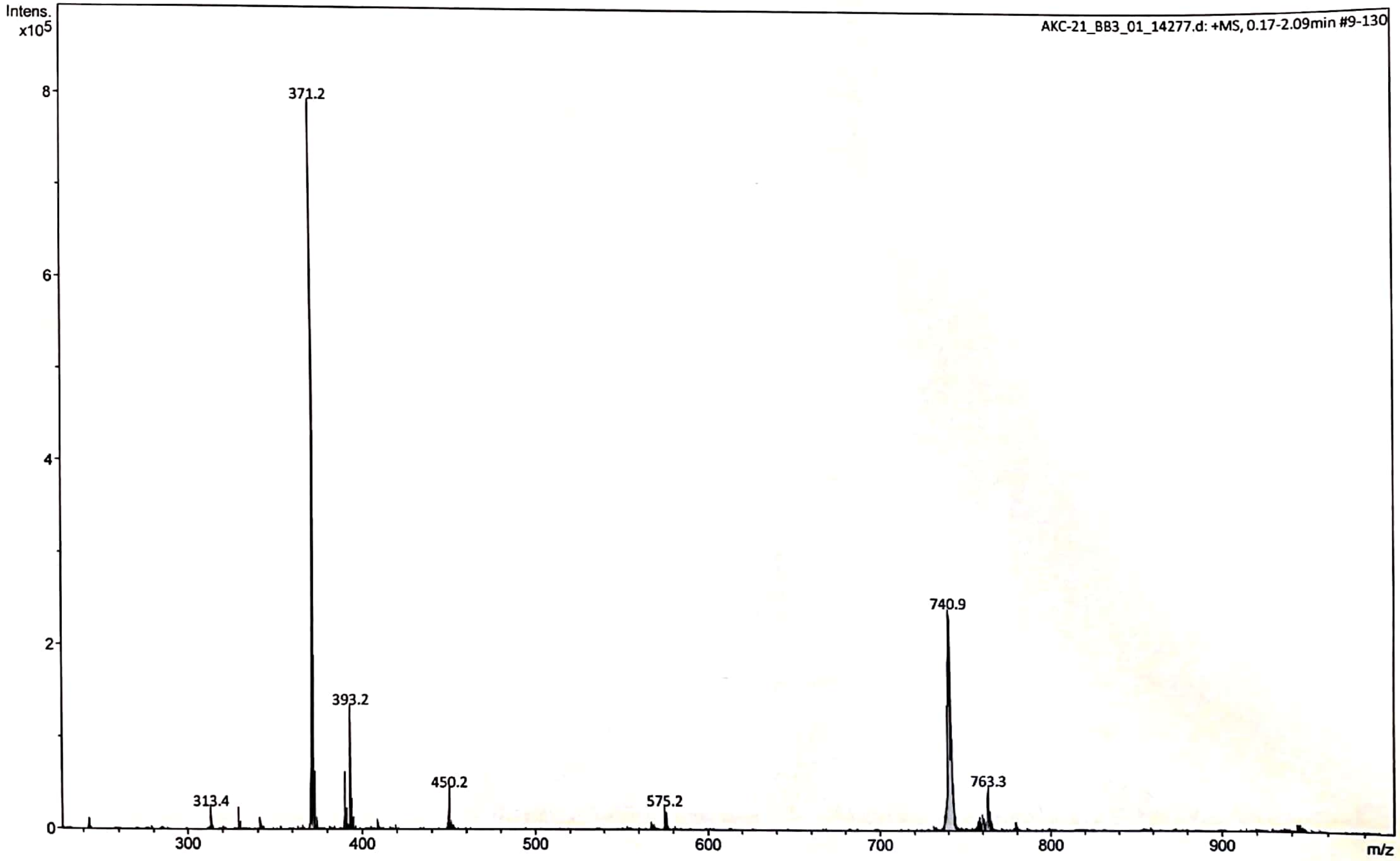

Scanned with CamScanner

Window Display Report

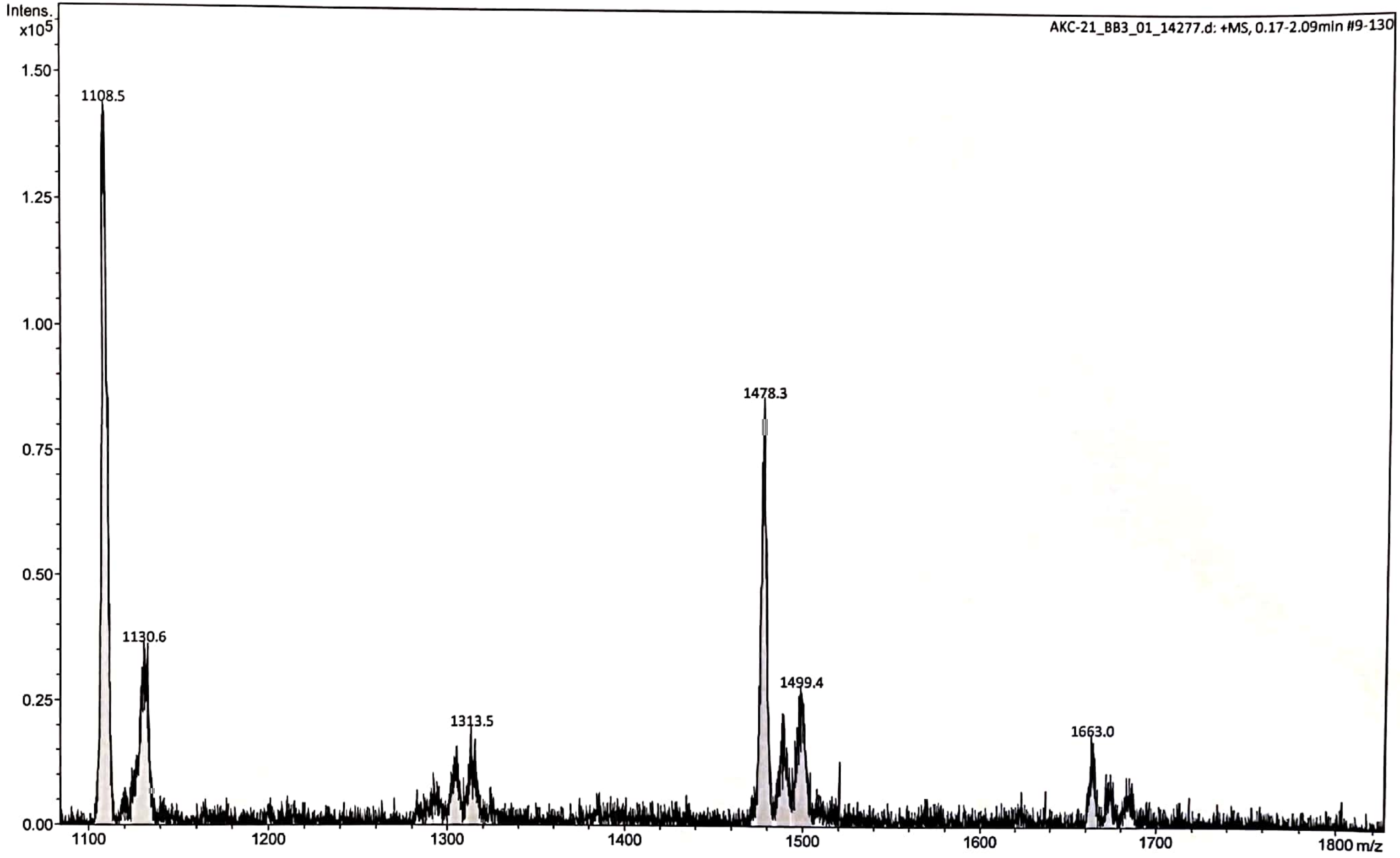

Scanned with CamScanner

Window Display Report

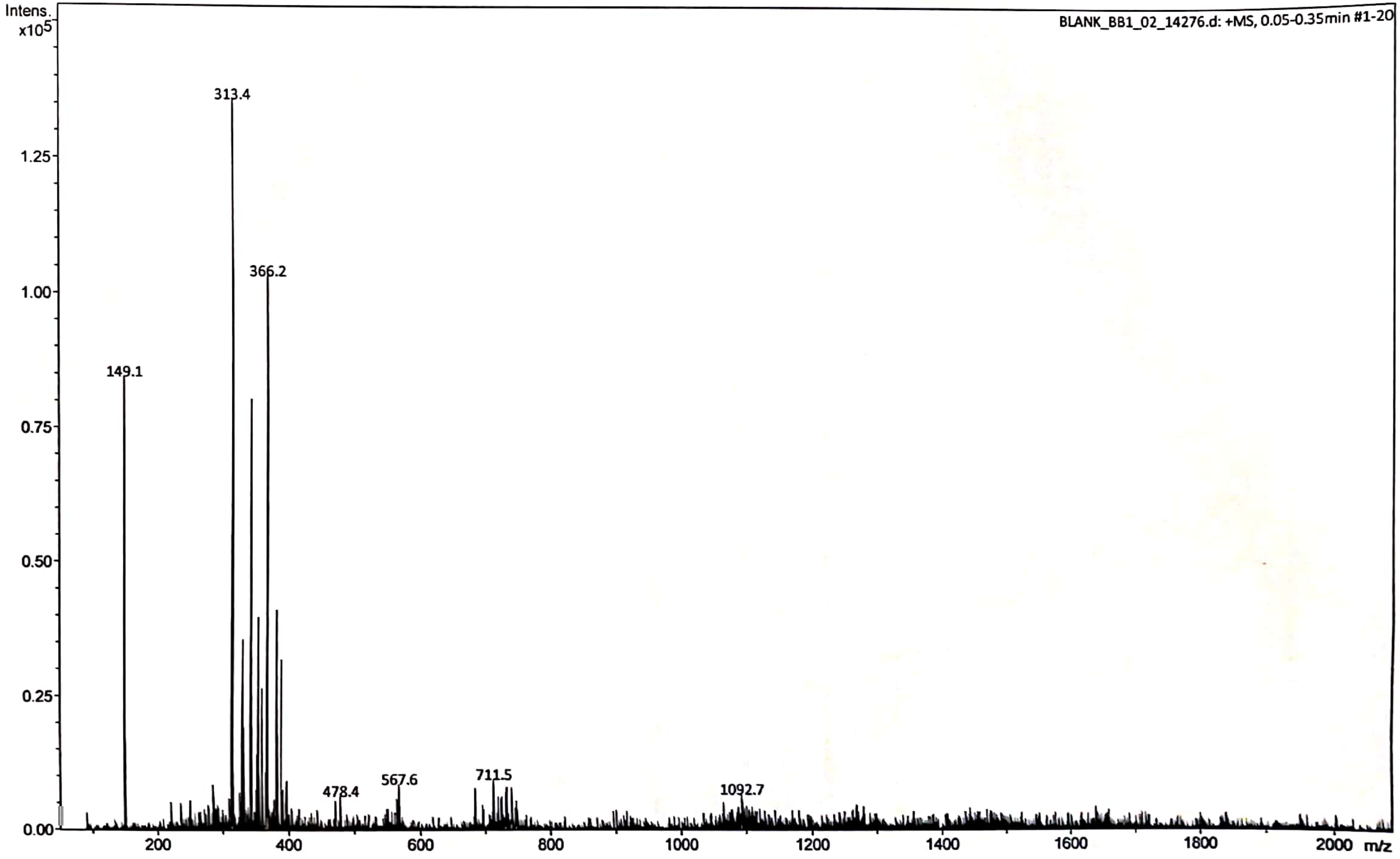

Scanned with CamScanner

Window Display Report

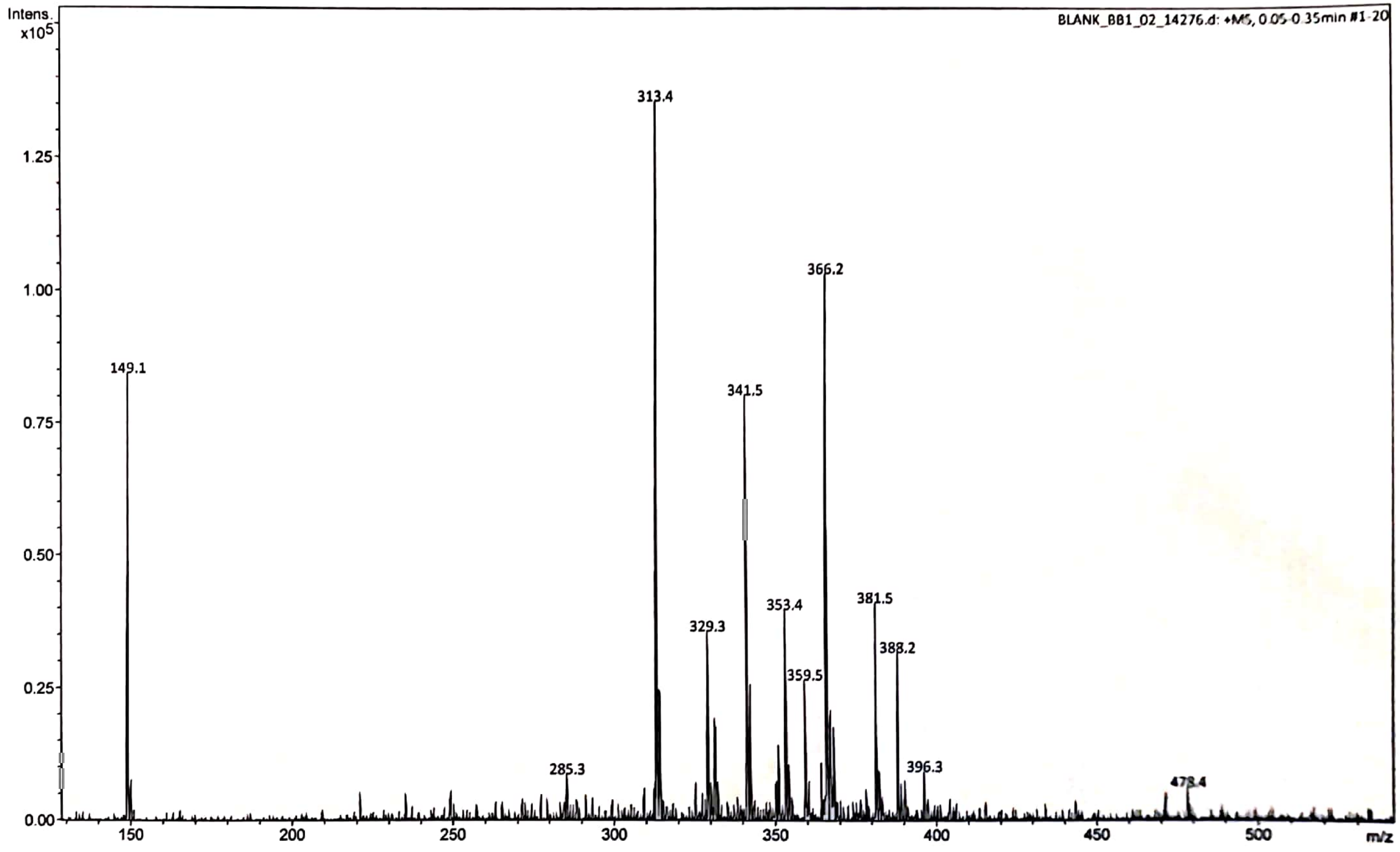

Scanned with CamScanner

Window Display Report

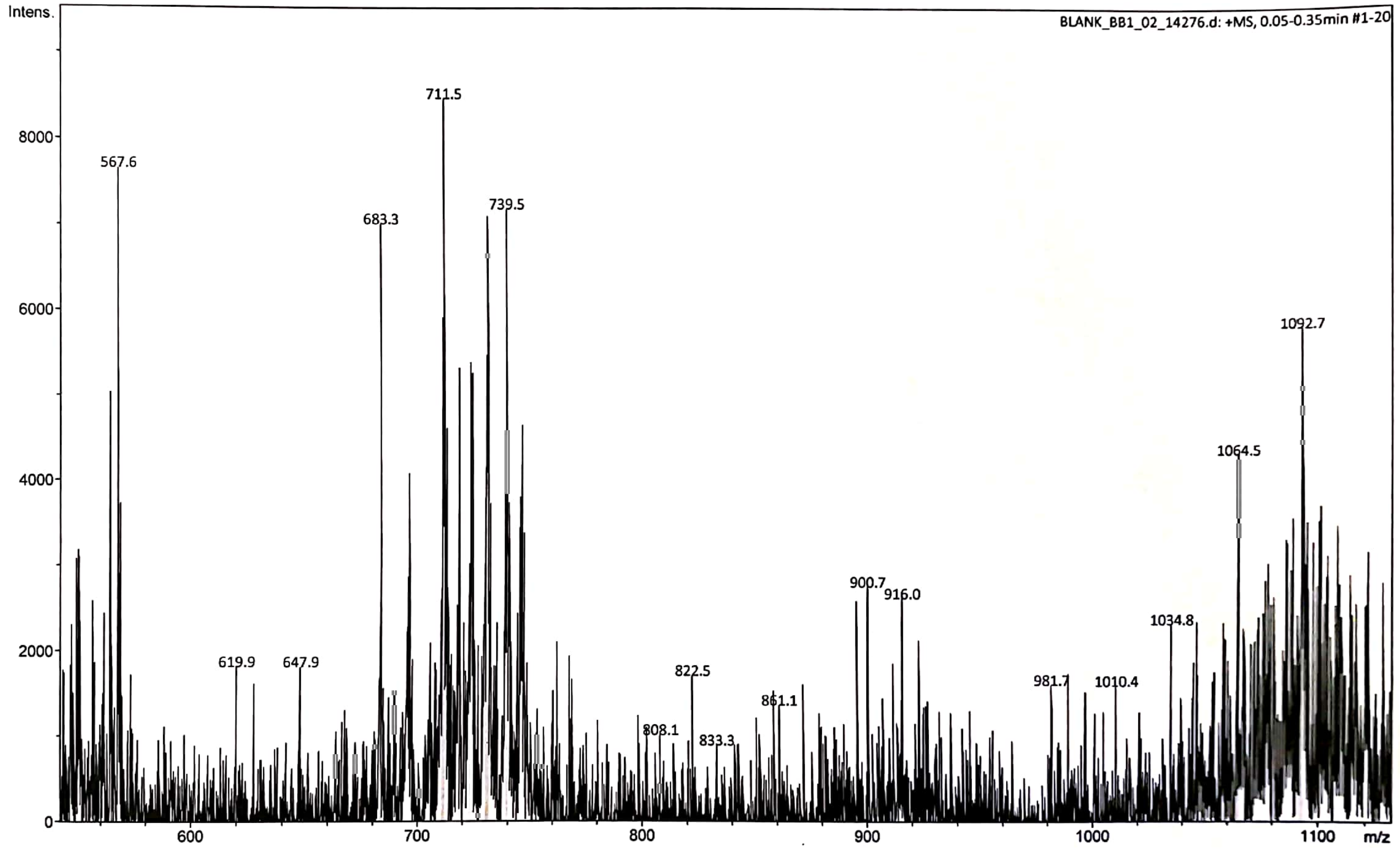

Scanned with CamScanner

Dr. Amara.185.fid — AKC-21/CDCl3/Adil/Dr. Amara Mumtaz/CUI-ATD

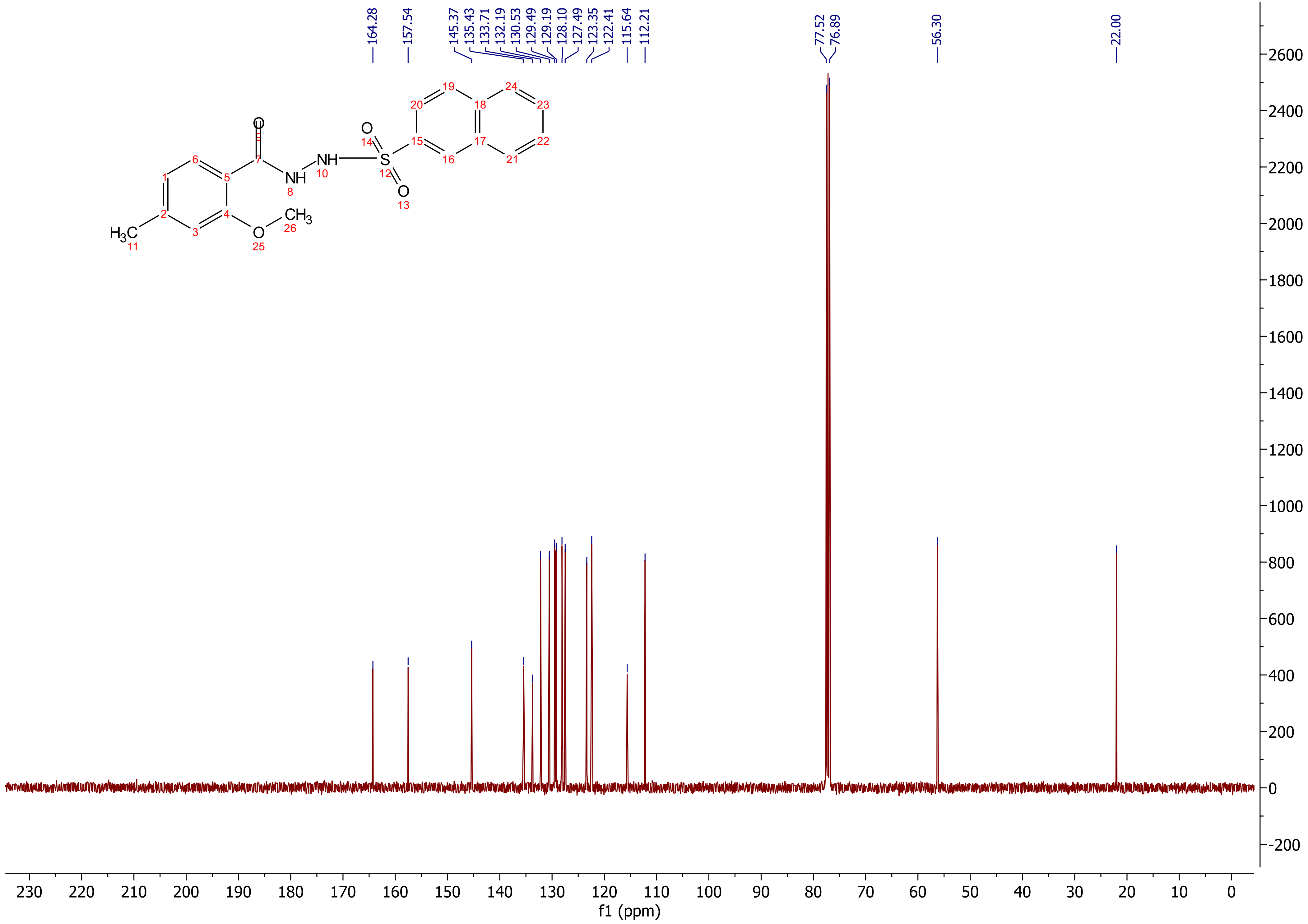

Window Display Report

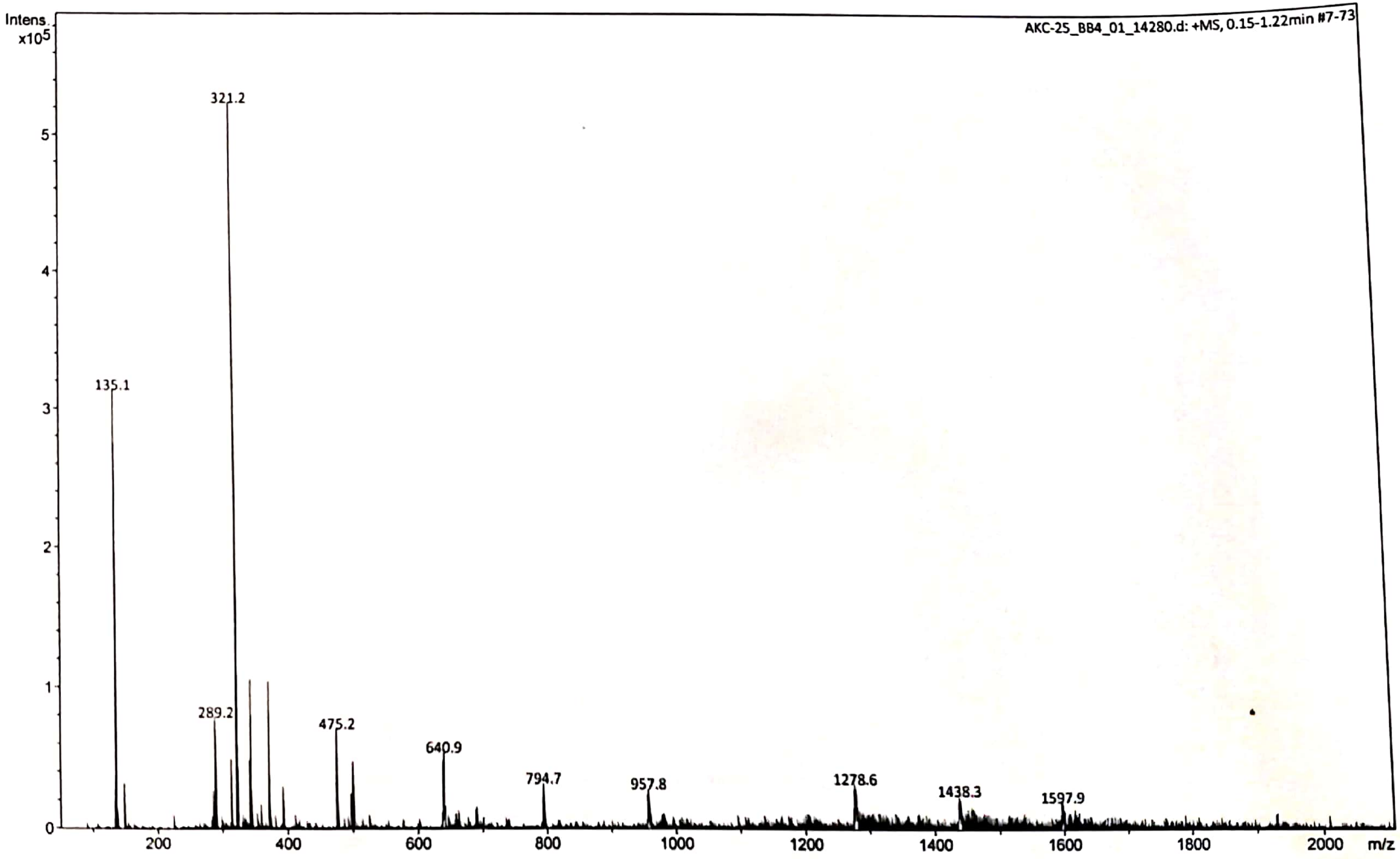

Scanned with CamScanner

Window Display Report

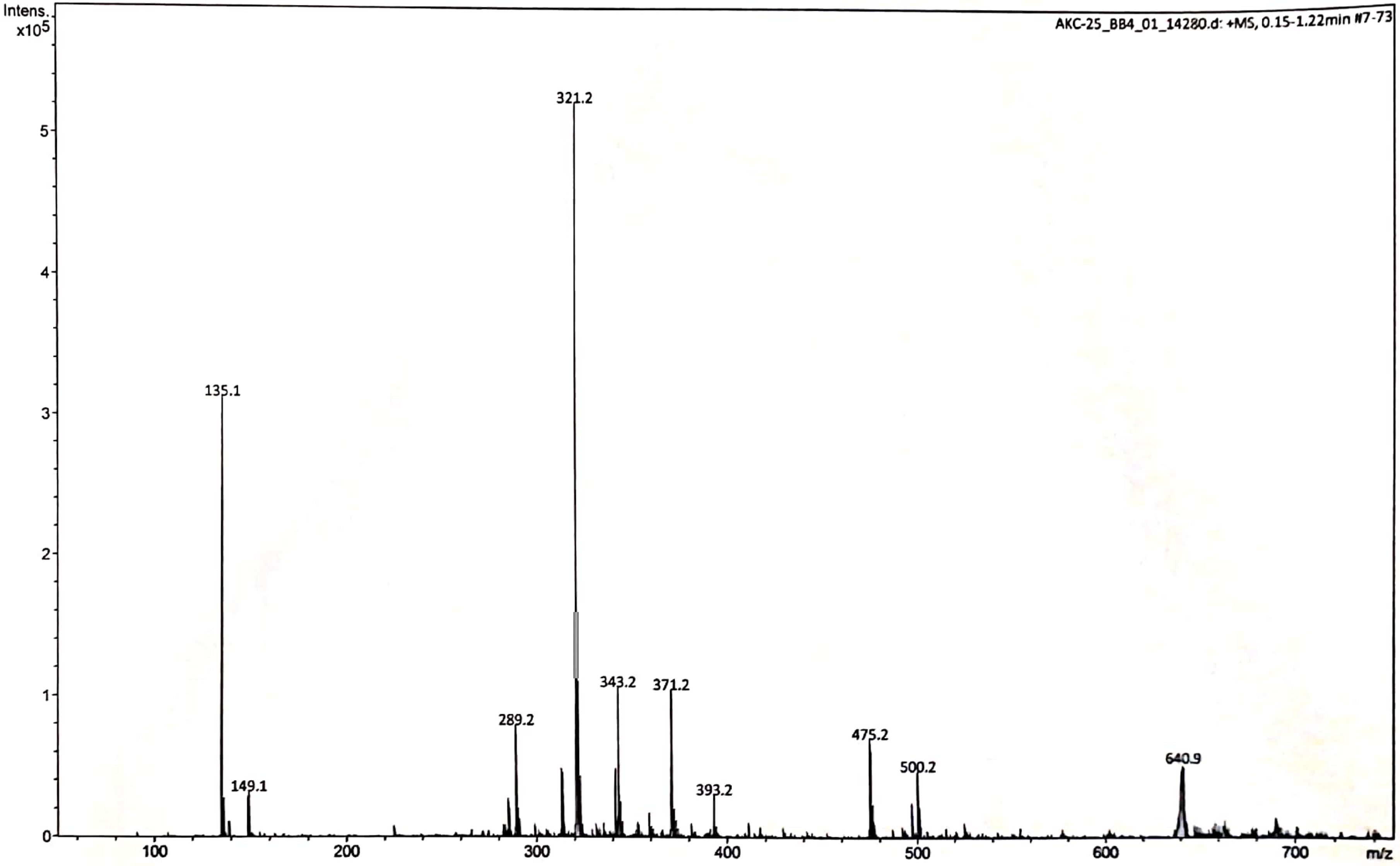

Scanned with CamScanner

Window Display Report

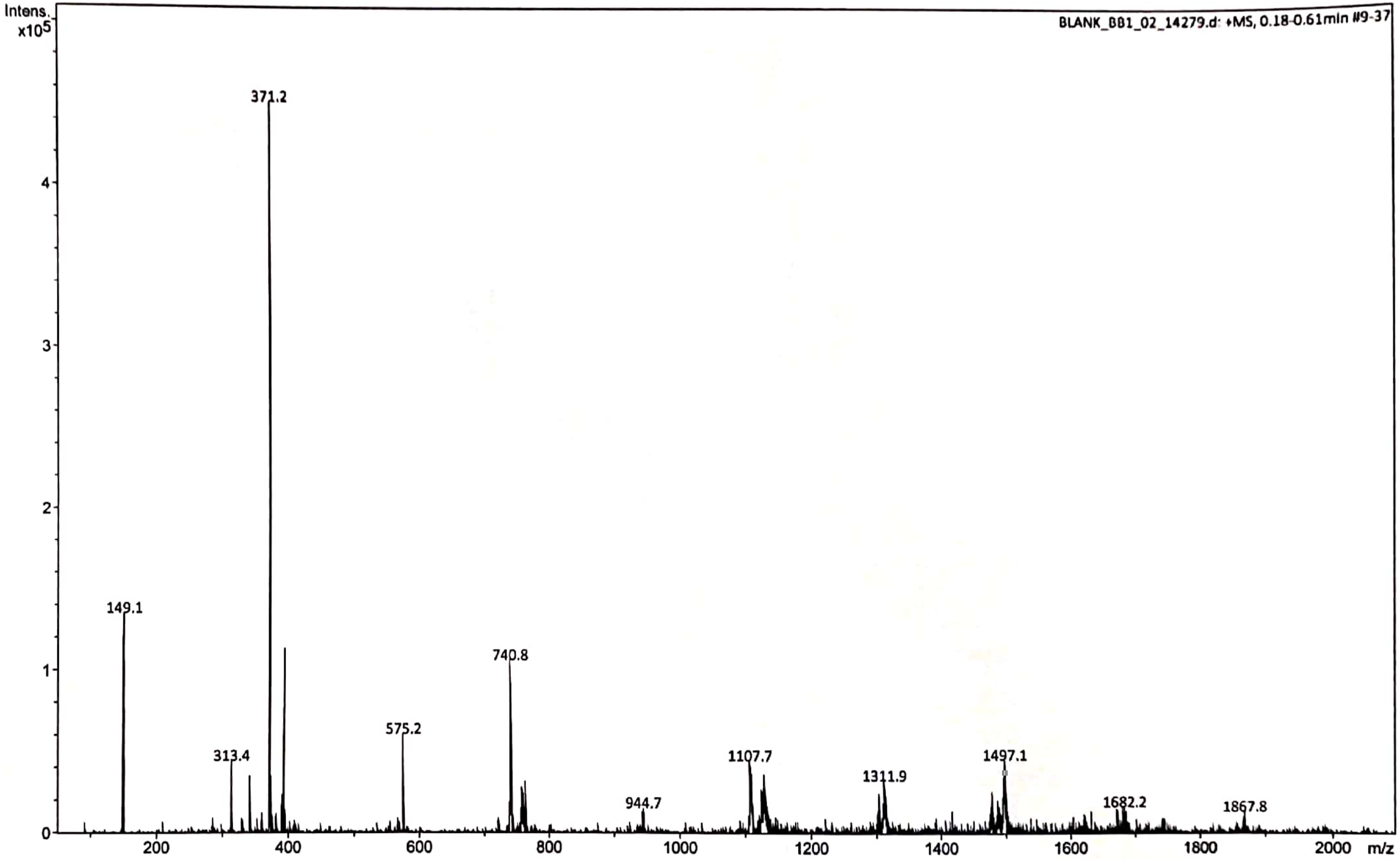

Scanned with CamScanner

Window Display Report

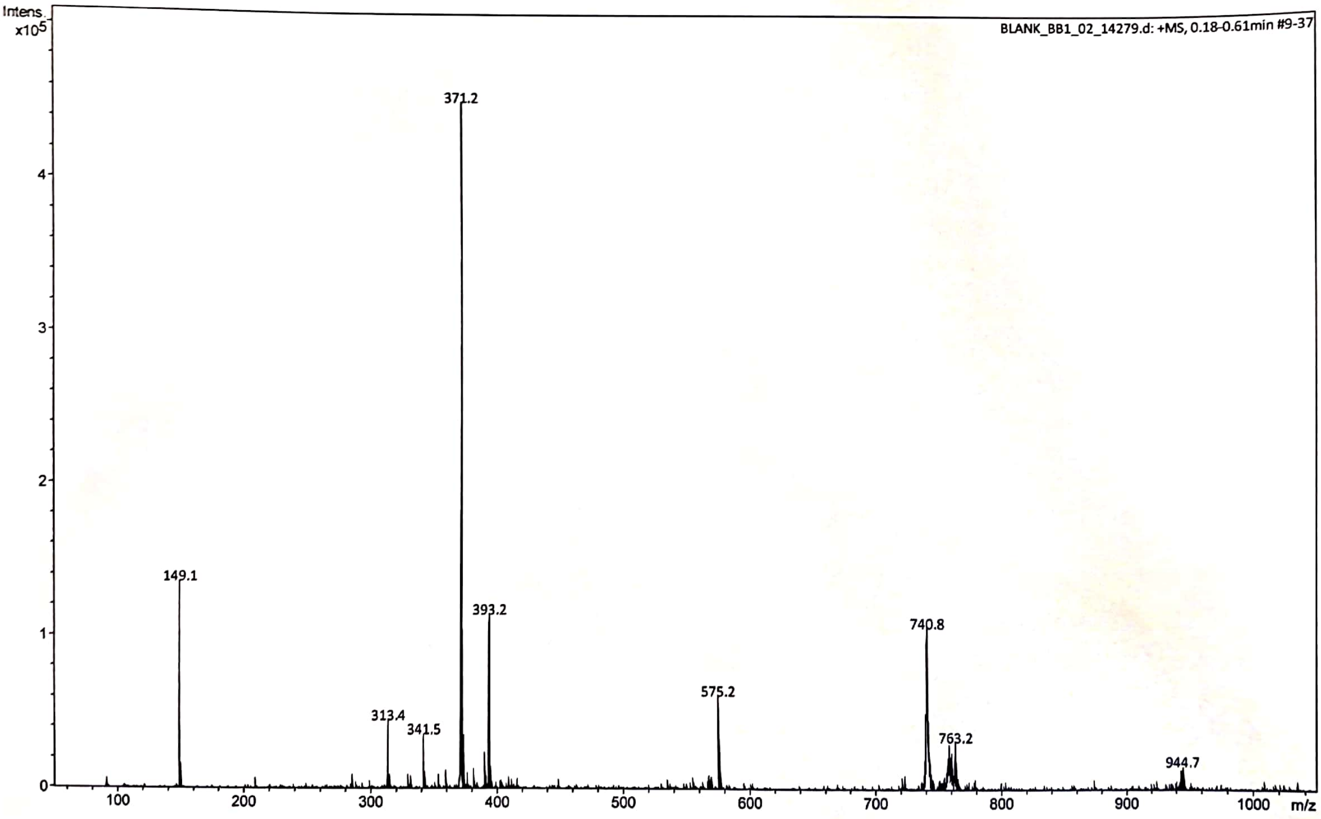

Scanned with CamScanner

Peak Summary Report

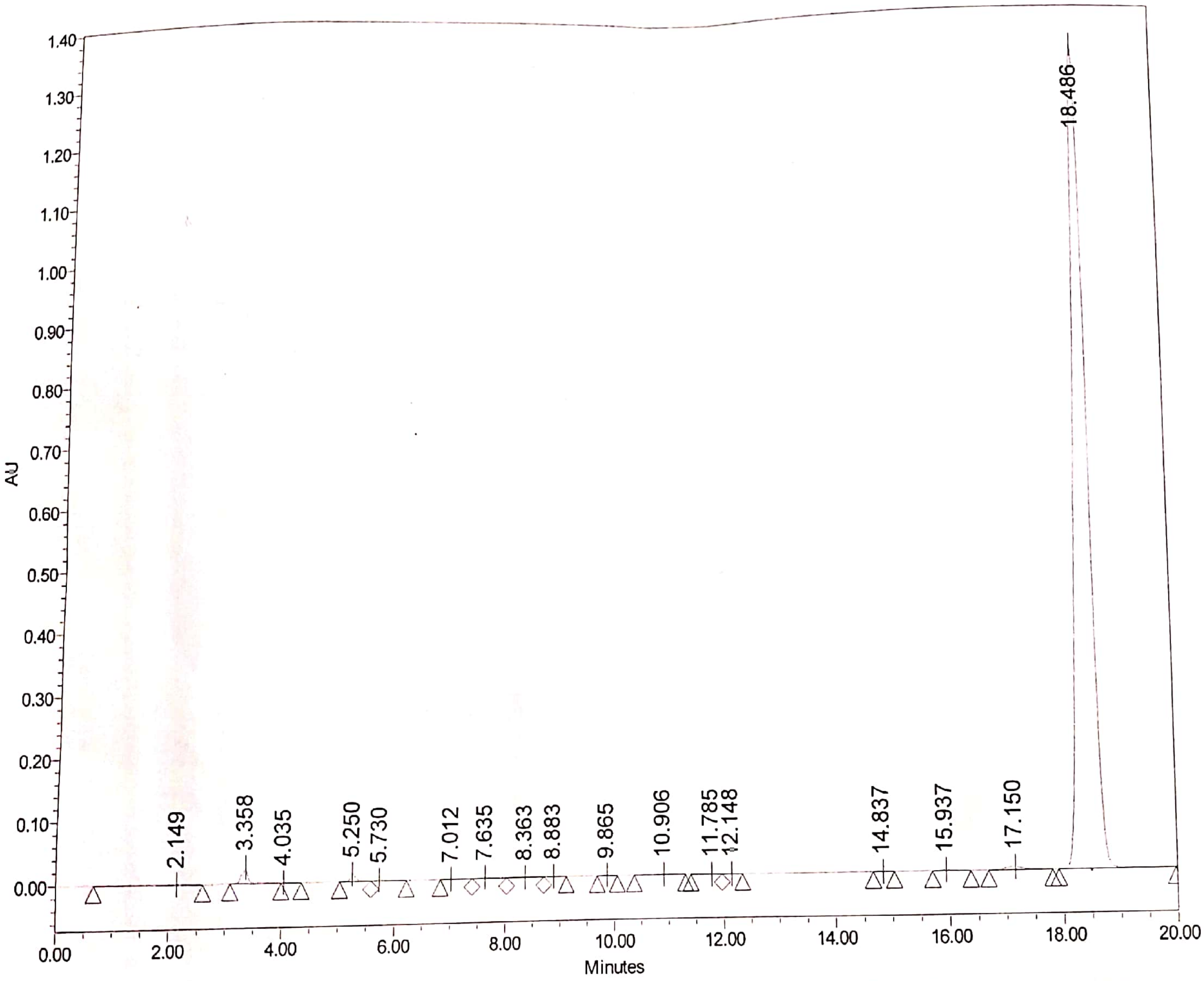

Sample Name: AKC 21; Date Acquired: 20-Dec-21 5:55:52 PM PKT; Vial: 12; Injection: 1

Peak Summary with Statistics

Name:

	Sample Name	Vial	Inj	Retention Time (min)	Area	% Area	Height
1	AKC 21	12	1	2.149	51896	0.22	1200
2	AKC 21	12	1	18.486	22668377	97.42	1339714
3	AKC 21	12	1	4.035	12326	0.05	1269
4	AKC 21	12	1	5.250	87971	0.38	12083

Reported by User: System
Report Method: Peak Summary Report
Report Method ID14557
Page: 1 of 2

Project Name: Neurotransmiter\NT Analysis
Date Printed:
21-Dec-21
3:11:02 PM Asia/Karachi

Scanned with CamScanner

Peak Summary Report

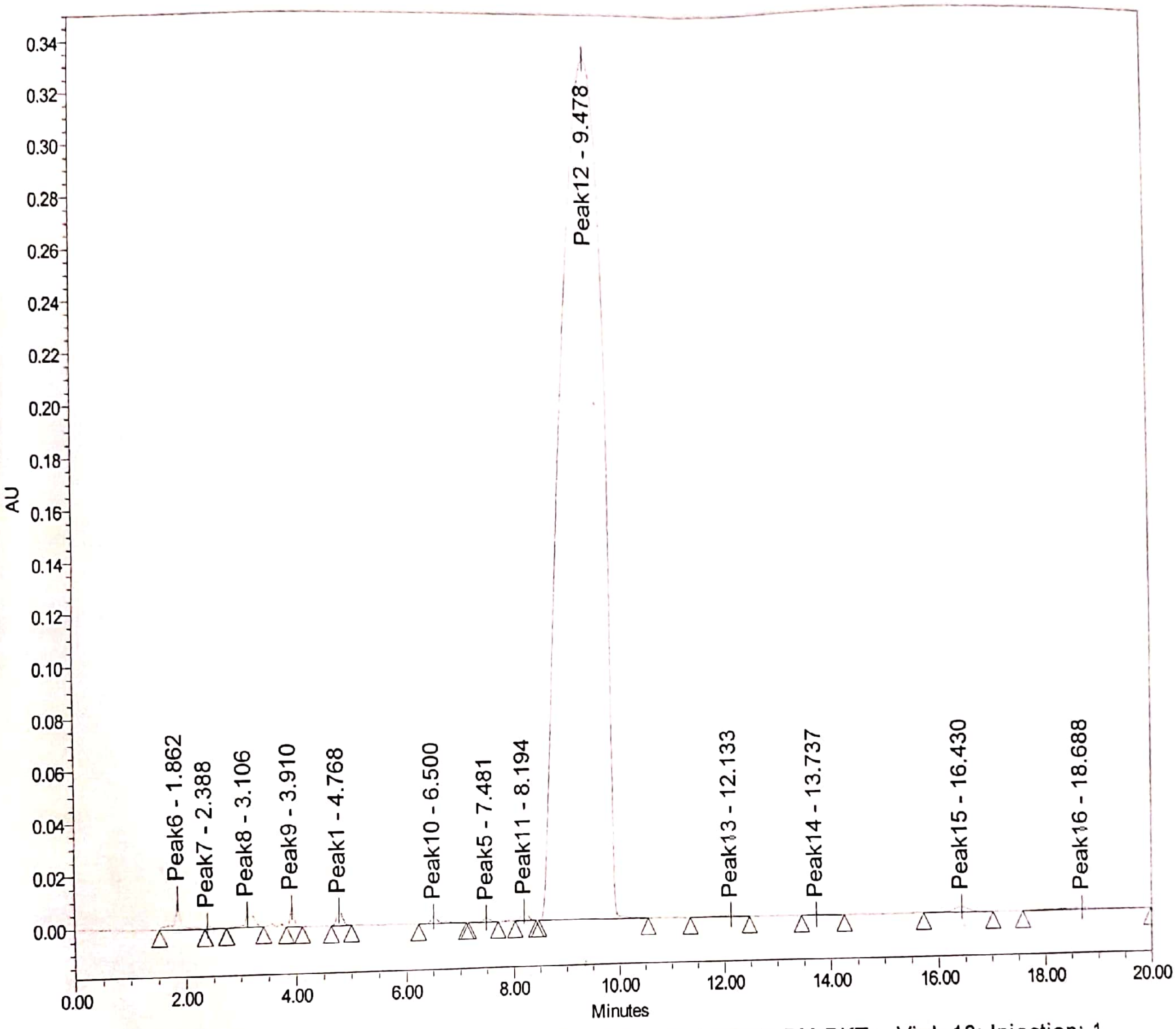

Sample Name: WKS11; Date Acquired: 20-Dec-21 6:16:38 PM PKT; Vial: 13; Injection: 1

Peak Summary with Statistics
Name: Peak1

	Sample Name	Vial	Inj	Name	Retention Time (min)	Area	% Area	Height
1	WKS11	13	1	Peak1	4.768	43153	0.24	5808
Mean					4.768			
Std. Dev.								
% RSD								

Scanned with CamScanner

Empower™ 3 SOFTWARE

Peak Summary Report

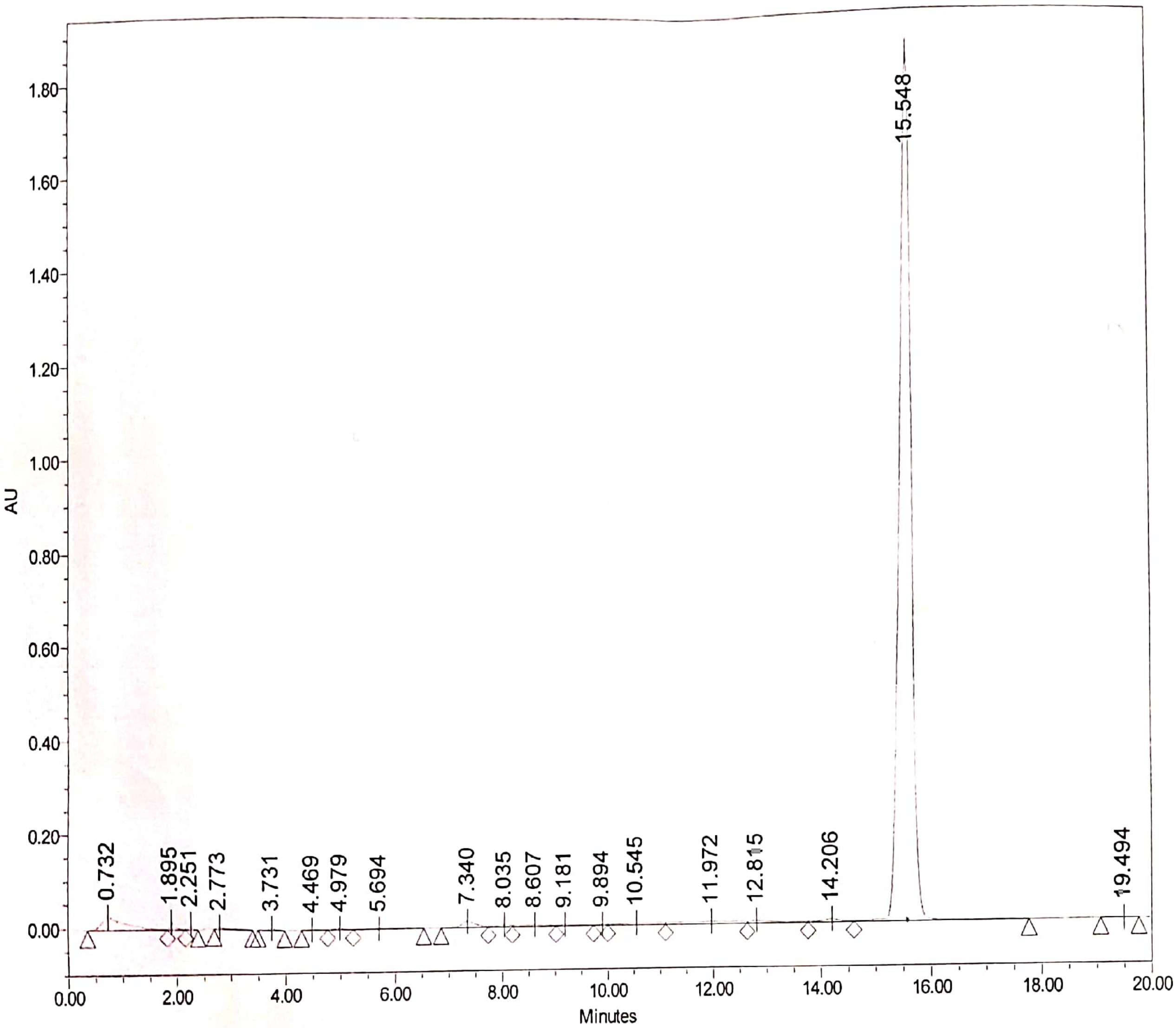

Sample Name: AK18; Date Acquired: 20-Dec-21 5:35:09 PM PKT; Vial: 11; Injection: ʼ

Peak Summary with Statistics
Name:

	Sample Name	Vial	Inj	Retention Time (min)	Area	% Area	Height
1	AK18	11	1	0.732	892907	2.96	28833
2	AK18	11	1	19.494	4314	0.01	278
3	AK18	11	1	2.251	53147	0.18	13889
4	AK18	11	1	2.773	73682	0.24	4747

Reported by User: System
Report Method: Peak Summary Report
Report Method ID14357

Project Name: Neurotransmiter\NT Analysis
Date Printed:
21-Dec-21
3:11:31 PM Asia/Karachi

Scanned with CamScanner
