# Supplementary material for: Synthesis, Carbonic Anhydrase II/IX/XII Inhibition, DFT, and Molecular Docking Studies of Hydrazide-Sulfonamide Hybrids of 4-Methylsalicyl- and Acyl-Substituted Hydrazide
Source: Biomed Res Int. 2022 Feb 24;2022:5293349. doi: 10.1155/2022/5293349 (PMC8894010; doi:10.1155/2022/5293349)
Supplement: Supplementary Materials — The supplementary material files contain the 1HNMR spectra and DFT tables of all the hydrazide-sulfonamide hybrids while 13CNMR, EIMS spectra, and HPLC graphs of the selected compounds. [file 5293349.f1.zip › Supporting Informations NMR spectra and DFT tables.pdf]

Synthesis, carbonic anhydrase II/IX/XII inhibition, DFT and molecular docking studies of hydrazide-sulfonamide hybrids of 4-methylsalicyl and acyl substituted hydrazide

Tables

Table 3. Comparison of Experimental and Computed NMR data for compound **19.**

Compound **19**				
**Carbon No.**	**Carbon Type**	**^{1}H-NMR (Experimental) δ, ppm**	**^{1}H-NMR (Computed) δ, ppm**	**Δδ, ppm**
3	CH	6.92	7.06	-0.14
5	CH	6.78	7.08	-0.3
6	CH	7.26	8.42	-1.16
2'	CH	8.39	8.22	0.17
3'	CH	8.09	8.56	-0.47
5'	CH	8.09	8.7	-0.61
6'	CH	8.39	10.01	-1.62
4-Me	CH_3	2.32	2.24	0.08
2-OMe	CH_3	3.80	3.74	0.06
Mean Absolute Error (MAE) = 0.19<br>Root Mean Square Error (RMSE) = 0.44				

Table 3a. Comparison of Experimental and Computed NMR data for compound **20.**

Compound **20**				
**Carbon No.**	**Carbon Type**	**^{1}H-NMR (Experimental) δ, ppm**	**^{1}H-NMR (Computed) δ, ppm**	**Δδ, ppm**
3	CH	6.81	7.16	-0.35
5	CH	6.86	7.26	-0.4
6	CH	7.72	8.3	-0.58
2'	CH	7.77	8.03	-0.26
3'	CH	7.59	7.6	-0.01

5'	CH	7.59	7.79	-0.2
6'	CH	7.77	9.56	-1.79
4-Me	CH_3	2.41	2.23	0.18
2-OMe	CH_3	4.03	3.77	0.26
Mean Absolute Error (MAE) = 0.37<br>Root Mean Square Error (RMSE) = 0.40				

Table 3b. Comparison of Experimental and Computed NMR data for compound **21.**

Compound **21**				
**Carbon No.**	**Carbon Type**	**^{1}H-NMR (Experimental) δ, ppm**	**^{1}H-NMR (Computed) δ, ppm**	**Δδ, ppm**
3	CH	6.80	7.14	-0.34
5	CH	6.83	7.25	-0.42
6	CH	7.72	8.31	-0.59
2'	CH	7.83	8.08	-0.25
3'	CH	6.90	6.91	-0.01
5'	CH	6.90	7.18	-0.28
6'	CH	7.83	9.30	-1.47
4-Me	CH_3	2.40	2.25	0.15
2-OMe	CH_3	3.83	3.80	0.03
4'-OMe	CH_3	4.02	3.62	0.4
Mean Absolute Error (MAE) = 0.16<br>Root Mean Square Error (RMSE) = 0.36				

Table 3c. Comparison of Experimental and Computed NMR data for compound **22.**

Compound **22**				
**Carbon No.**	**Carbon Type**	**^{1}H-NMR (Experimental) δ, ppm**	**^{1}H-NMR (Computed) δ, ppm**	**Δδ, ppm**
3	CH	6.78	7.16	-0.38
5	CH	6.75	7.25	-0.5
6	CH	7.37	8.31	-0.94
2'	CH	7.87	7.98	-0.11
3'	CH	7.40	7.38	0.02
5'	CH	7.40	7.65	-0.25
6'	CH	7.87	9.35	-1.48
4-Me	CH_3	2.44	2.25	0.19
4'-Me	CH_3	2.30	2.31	-0.01
2-OMe	CH_3	3.88	3.79	0.09
Mean Absolute Error (MAE) = 0.17<br>Root Mean Square Error (RMSE) = 0.38				

Table 3d. Comparison of Experimental and Computed NMR data for compound **23.**

Compound 23				
**Carbon No.**	**Carbon Type**	**^{1}H-NMR (Experimental) δ, ppm**	**^{1}H-NMR (Computed) δ, ppm**	**Δδ, ppm**
3	CH	8.48	6.84	1.64
5	CH	6.74	7.03	-0.29
6	CH	7.63	8.44	-0.81
2'	CH	6.78	8.81	-2.03
4'	CH	7.87	8.4	-0.53
5'	CH	7.88	7.97	-0.09
6'	CH	7.89	8.04	-0.15
7'	CH	7.91	8.35	-0.44
9'	CH	7.56	8.43	-0.87
10'	CH	7.56	9.55	-1.99
4-Me	CH_3	2.37	2.35	0.02
2-OMe	CH_3	4.01	3.85	0.16
Mean Absolute Error (MAE) = 0.38<br>Root Mean Square Error (RMSE) = 0.73				

Table 3e. Comparison of Experimental and Computed NMR data for compound **24.**

Compound **24**				
**Carbon No.**	**Carbon Type**	**^{1}H-NMR (Experimental) δ, ppm**	**^{1}H-NMR (Computed) δ, ppm**	**Δδ, ppm**
3	CH	6.71	6.77	-0.06
5	CH	6.71	7.05	-0.34
6	CH	7.58	8.37	-0.79
2'	CH	8.39	8.22	0.17
3'	CH	8.08	8.57	-0.49
5'	CH	8.08	8.71	-0.63
6'	CH	8.39	10.01	-1.62
4-Me	CH_3	2.25	2.17	0.08
Mean Absolute Error (MAE) = 0.17<br>Root Mean Square Error (RMSE) = 0.41				

Table 3f. Comparison of Experimental and Computed NMR data for compound **25.**

Compound **25**				
**Carbon No.**	**Carbon Type**	**^{1}H-NMR (Experimental) δ, ppm**	**^{1}H-NMR (Computed) δ, ppm**	**Δδ, ppm**
3	CH	6.78	6.62	0.16
5	CH	6.72	7.04	-0.32
6	CH	7.33	8.47	-1.14
2'	CH	7.78	8.03	-0.25
3'	CH	7.62	7.59	0.03
5'	CH	7.62	7.79	-0.17
6'	CH	7.78	9.71	-1.93
4-Me	CH_3	2.19	2.2	-0.01
Mean Absolute Error (MAE) = 0.17<br>Root Mean Square Error (RMSE) = 0.46				

Table 3g. Comparison of Experimental and Computed NMR data for compound **26.**

Compound **26**				
**Carbon No.**	**Carbon Type**	**^{1}H-NMR (Experimental) δ, ppm**	**^{1}H-NMR (Computed) δ, ppm**	**Δδ, ppm**
3	CH	6.79	6.61	0.18
5	CH	6.87	7.04	-0.17
6	CH	7.74	8.48	-0.74
2'	CH	7.85	8.07	-0.22
3'	CH	6.93	6.91	0.02
5'	CH	6.93	7.17	-0.24
6'	CH	7.85	9.42	-1.57
4-Me	CH_3	2.42	2.2	0.22
4'-OMe	CH_3	3.86	3.61	0.25
Mean Absolute Error (MAE) = 0.15<br>Root Mean Square Error (RMSE) = 0.37				

Table 3h. Comparison of Experimental and Computed NMR data for compound **27.**

Compound **27**				
**Carbon No.**	**Carbon Type**	**^{1}H-NMR (Experimental) δ, ppm**	**^{1}H-NMR (Computed) δ, ppm**	**Δδ, ppm**
3	CH	6.76	6.62	0.14
5	CH	6.73	7.04	-0.31
6	CH	7.32	8.48	-1.16
2'	CH	7.81	7.97	-0.16
3'	CH	7.43	7.37	0.06
5'	CH	7.43	7.65	-0.22
6'	CH	7.81	9.45	-1.64
4-Me	CH_3	2.42	2.2	0.22
4'-Me	CH_3	2.34	2.31	0.03
Mean Absolute Error (MAE) = 0.16<br>Root Mean Square Error (RMSE) = 0.42				

Table 3i. Comparison of Experimental and Computed NMR data for compound **28.**

Compound **28**				
**Carbon No.**	**Carbon Type**	**^{1}H-NMR (Experimental) δ, ppm**	**^{1}H-NMR (Computed) δ, ppm**	**Δδ, ppm**
2	CH	7.06	7.34	-0.28
3	CH	6.97	7.35	-0.38
5	CH	6.97	7.44	-0.47
6	CH	7.06	7.42	-0.36
7	CH_2	3.22	3.52	-0.3
2'	CH	8.18	8.13	0.05
3'	CH	7.90	8.52	-0.62
5'	CH	7.90	8.65	-0.75
6'	CH	8.18	9.85	-1.67
4-Me	CH_3	2.28	2.17	0.11
Mean Absolute Error (MAE) = 0.21<br>Root Mean Square Error (RMSE) = 0.42				

Table 3j. Comparison of Experimental and Computed NMR data for compound **29.**

Compound **29**				
**Carbon No.**	**Carbon Type**	**^{1}H-NMR (Experimental) δ, ppm**	**^{1}H-NMR (Computed) δ, ppm**	**Δδ, ppm**
2	CH	7.50	7.42	0.08
3	CH	7.17	7.45	-0.28
5	CH	7.17	7.54	-0.37
6	CH	7.50	7.52	-0.02
7	CH_2	3.37	3.59	-0.22
2'	CH	7.99	7.99	0
3'	CH	7.62	7.68	-0.06
5'	CH	7.62	7.82	-0.2
6'	CH	7.99	9.57	-1.58
4-Me	CH_3	2.40	2.27	0.13
Mean Absolute Error (MAE) = 0.12<br>Root Mean Square Error (RMSE) = 0.34				

Table 3k. Comparison of Experimental and Computed NMR data for compound **30.**

Compound **30**				
**Carbon No.**	**Carbon Type**	**^{1}H-NMR (Experimental) δ, ppm**	**^{1}H-NMR (Computed) δ, ppm**	**Δδ, ppm**
2	CH	7.00	7.29	-0.29
3	CH	6.89	7.33	-0.44
5	CH	6.89	7.49	-0.6
6	CH	7.00	7.54	-0.54
7	CH_2	3.37	3.55	-0.18
2'	CH	7.73	7.99	-0.26
3'	CH	7.15	6.88	0.27
5'	CH	7.15	7.13	0.02
6'	CH	7.73	9.2	-1.47
4-Me	CH_3	2.37	2.24	0.13
4'-OME	CH_3	3.87	3.59	0.28
Mean Absolute Error (MAE) = 0.19<br>Root Mean Square Error (RMSE) = 0.37				

Table 3l. Comparison of Experimental and Computed NMR data for compound **31.**

Compound **31**				
**Carbon No.**	**Carbon Type**	**^{1}H-NMR (Experimental) δ, ppm**	**^{1}H-NMR (Computed) δ, ppm**	**Δδ, ppm**
2	CH	7.15	7.3	-0.15
3	CH	7.00	7.36	-0.36
5	CH	7.00	7.48	-0.48
6	CH	7.15	7.53	-0.38
7	CH_2	3.36	3.56	-0.2
2'	CH	7.69	7.89	-0.2
3'	CH	7.22	7.33	-0.11
5'	CH	7.22	7.61	-0.39
6'	CH	7.69	9.25	-1.56
4-Me	CH_3	2.42	2.24	0.18
4'-Me	CH_3	2.38	2.28	0.1
Mean Absolute Error (MAE) = 0.17<br>Root Mean Square Error (RMSE) = 0.36				

Table 3m. Comparison of Experimental and Computed NMR data for compound **32.**

Compound **32**				
**Carbon No.**	**Carbon Type**	**^{1}H-NMR (Experimental) δ, ppm**	**^{1}H-NMR (Computed) δ, ppm**	**Δδ, ppm**
2	CH	6.64	6.75	-0.11
4	CH	6.80	6.89	-0.09
5	CH	7.18	7.5	-0.32
6	CH	6.69	7.08	-0.39
7	CH_2	3.26	3.375	-0.115
2'	CH	8.21	8.19	0.02
3'	CH	7.90	8.54	-0.64
5'	CH	7.90	8.6	-0.7
6'	CH	8.21	9.77	-1.56
3-OMe	CH_3	3.71	3.47	0.24
Mean Absolute Error (MAE) = 0.17<br>Root Mean Square Error (RMSE) = 0.39				

Table 3n. Comparison of Experimental and Computed NMR data for compound **33.**

Compound **33**				
**Carbon No.**	**Carbon Type**	**^{1}H-NMR (Experimental) δ, ppm**	**^{1}H-NMR (Computed) δ, ppm**	**Δδ, ppm**
2	CH	6.66	7.13	-0.47
4	CH	6.70	6.85	-0.15
5	CH	7.63	7.36	0.27
6	CH	6.90	6.74	0.16
7	CH_2	3.38	3.33	0.05
2'	CH	7.62	9.44	-1.82
3'	CH	7.53	7.73	-0.2
5'	CH	7.53	7.58	-0.05
6'	CH	7.62	8.01	-0.39
3-OMe	CH_3	3.84	3.56	0.28
Mean Absolute Error (MAE) = 0.16<br>Root Mean Square Error (RMSE) = 0.40				

Table 3o. Comparison of Experimental and Computed NMR data for compound **34.**

Compound **34**				
**Carbon No.**	**Carbon Type**	**^{1}H-NMR (Experimental) δ, ppm**	**^{1}H-NMR (Computed) δ, ppm**	**Δδ, ppm**
2	CH	6.67	7.15	-0.48
4	CH	6.87	6.84	0.03
5	CH	6.87	7.36	-0.49
6	CH	6.87	6.74	0.13
7	CH_2	3.38	3.34	0.04
2'	CH	7.73	9.15	-1.42
3'	CH	6.71	7.11	-0.4
5'	CH	6.71	6.89	-0.18
6'	CH	7.73	8.06	-0.33
3-OMe	CH_3	3.88	3.55	0.33
4'-OMe	CH_3	3.82	3.59	0.23
Mean Absolute Error (MAE) = 0.17<br>Root Mean Square Error (RMSE) = 0.35				

Table 3p. Comparison of Experimental and Computed NMR data for compound **35.**

Compound **35**				
**Carbon No.**	**Carbon Type**	**^{1}H-NMR (Experimental) δ, ppm**	**^{1}H-NMR (Computed) δ, ppm**	**Δδ, ppm**
2	CH	6.67	7.38	-0.71
4	CH	6.70	6.88	-0.18
5	CH	7.23	7.29	-0.06
6	CH	6.87	6.74	0.13
7	CH_2	3.37	3.38	-0.01
2'	CH	7.79	9.26	-1.47
3'	CH	7.68	7.62	0.06
5'	CH	7.68	7.36	0.32
6'	CH	7.79	7.97	-0.18
4'-Me	CH_3	2.42	2.3	0.12
3-OMe	CH_3	3.82	3.65	0.17
Mean Absolute Error (MAE) = 0.14<br>Root Mean Square Error (RMSE) = 0.34				

Table 18. Comparison of Experimental and Computed NMR data for compound **36.**

Compound **36**				
**Carbon No.**	**Carbon Type**	**^{1}H-NMR (Experimental) δ, ppm**	**^{1}H-NMR (Computed) δ, ppm**	**Δδ, ppm**
2	CH	6.59	7.21	-0.62
4	CH	6.81	6.95	-0.14
5	CH	7.15	7.42	-0.27
6	CH	7.39	6.9	0.49
7	CH_2	3.31	3.58	-0.27
2'	CH	8.41	8.79	-0.38
4'	CH	7.80	8.38	-0.58
5'	CH	7.92	7.95	-0.03
6'	CH	7.92	8.01	-0.09
7'	CH	7.92	8.31	-0.39
9'	CH	7.66	8.35	-0.69
10'	CH	7.66	9.38	-1.72
3-OMe	CH_3	3.75	3.68	0.07
Mean Absolute Error (MAE) = 0.24<br>Root Mean Square Error (RMSE) = 0.45				
